# Assessing the Pragmatic Nature of Mobile Health Interventions Promoting Physical Activity: Systematic Review and Meta-analysis

**DOI:** 10.2196/43162

**Published:** 2023-05-04

**Authors:** Chad Stecher, Bjorn Pfisterer, Samantha M Harden, Dana Epstein, Jakob M Hirschmann, Kathrin Wunsch, Matthew P Buman

**Affiliations:** 1 College of Health Solutions Arizona State University Phoenix, AZ United States; 2 Institute of Sports and Sports Science Karlsruhe Institute of Technology Karlsruhe Germany; 3 Department of Human Nutrition, Foods, and Exercise Virginia Tech Blacksburg, VA United States; 4 Institute of Sport Sciences Goethe University Frankfurt Germany

**Keywords:** physical activity, mobile health, mHealth, Reach, Effectiveness, Adoption, Implementation, Maintenance, RE-AIM, Pragmatic-Explanatory Continuum Indicator Summary-2, PRECIS-2, systematic review, meta-analysis, digital health, mobile phone

## Abstract

**Background:**

Mobile health (mHealth) apps can promote physical activity; however, the pragmatic nature (ie, how well research translates into real-world settings) of these studies is unknown. The impact of study design choices, for example, intervention duration, on intervention effect sizes is also understudied.

**Objective:**

This review and meta-analysis aims to describe the pragmatic nature of recent mHealth interventions for promoting physical activity and examine the associations between study effect size and pragmatic study design choices.

**Methods:**

The PubMed, Scopus, Web of Science, and PsycINFO databases were searched until April 2020. Studies were eligible if they incorporated apps as the primary intervention, were conducted in health promotion or preventive care settings, included a device-based physical activity outcome, and used randomized study designs. Studies were assessed using the Reach, Effectiveness, Adoption, Implementation, Maintenance (RE-AIM) and Pragmatic-Explanatory Continuum Indicator Summary-2 (PRECIS-2) frameworks. Study effect sizes were summarized using random effect models, and meta-regression was used to examine treatment effect heterogeneity by study characteristics.

**Results:**

Overall, 3555 participants were included across 22 interventions, with sample sizes ranging from 27 to 833 (mean 161.6, SD 193.9, median 93) participants. The study populations’ mean age ranged from 10.6 to 61.5 (mean 39.6, SD 6.5) years, and the proportion of males included across all studies was 42.8% (1521/3555). Additionally, intervention lengths varied from 2 weeks to 6 months (mean 60.9, SD 34.9 days). The primary app- or device-based physical activity outcome differed among interventions: most interventions (17/22, 77%) used activity monitors or fitness trackers, whereas the rest (5/22, 23%) used app-based accelerometry measures. Data reporting across the RE-AIM framework was low (5.64/31, 18%) and varied within specific dimensions (Reach=44%; Effectiveness=52%; Adoption=3%; Implementation=10%; Maintenance=12.4%). PRECIS-2 results indicated that most study designs (14/22, 63%) were *equally explanatory and pragmatic*, with an overall PRECIS-2 score across all interventions of 2.93/5 (SD 0.54). The most pragmatic dimension was *flexibility (adherence)*, with an average score of 3.73 (SD 0.92), whereas *follow-up*, *organization*, and *flexibility (delivery)* appeared more explanatory with means of 2.18 (SD 0.75), 2.36 (SD 1.07), and 2.41 (SD 0.72), respectively. An overall positive treatment effect was observed (Cohen *d*=0.29, 95% CI 0.13-0.46). Meta-regression analyses revealed that more pragmatic studies (−0.81, 95% CI −1.36 to −0.25) were associated with smaller increases in physical activity. Treatment effect sizes were homogenous across study duration, participants’ age and gender, and RE-AIM scores.

**Conclusions:**

App-based mHealth physical activity studies continue to underreport several key study characteristics and have limited pragmatic use and generalizability. In addition, more pragmatic interventions observe smaller treatment effects, whereas study duration appears to be unrelated to the effect size. Future app-based studies should more comprehensively report real-world applicability, and more pragmatic approaches are needed for maximal population health impacts.

**Trial Registration:**

PROSPERO CRD42020169102; https://www.crd.york.ac.uk/prospero/display_record.php?RecordID=169102

## Introduction

### Background

Regular physical activity can combat numerous chronic conditions and is associated with reduced premature mortality [1,2]. Despite these benefits, behavioral interventions and public policy have been largely unsuccessful in promoting higher physical activity among the general population. Worldwide, 28% of individuals are currently classified as insufficiently active [3], and physical inactivity has an estimated annual health care cost of >US $50 billion globally [4]. Thus, increasing physical activity across the world is an important economic and public health objective that requires scalable and pragmatic strategies [5].

Mobile health (mHealth) tools are one promising approach for improving health care delivery and scaling behavioral interventions worldwide [6,7]. Mobile app–based platforms can be particularly effective at increasing intervention accessibility and cost-effectiveness, and they offer the ability to tailor intervention methods to individuals’ unique needs [8-10]. Accordingly, the use of app-based mHealth tools in health care has rapidly increased since 2008 [10,11], and several review papers have recently highlighted the important potential role of app-based interventions for improving global physical activity levels [12-14]. In addition, app-based interventions saw a large relative increase in publications compared with SMS text messaging, telehealth, or web-based interventions [14], making app-based interventions one of the most popular new clinical tools [15] and an important intervention approach to review to inform current and future researchers, as well as health care providers (eg, general practitioners).

Despite the growth of research using app-based tools to promote physical activity, there is limited evidence that app-based interventions for increasing physical activity have been widely adopted by policy makers or integrated into clinical or other practice settings [16,17]. One potential explanation for this lack of real-world application is that this research has generally centered on internal validity (ie, reliability or accuracy of the outcomes) over external validity (ie, generalizability or applicability of results) [18,19]. In other words, the existing research has emphasized explanatory approaches rather than more pragmatic study designs [20]. Explanatory studies measure whether an intervention has a beneficial effect under ideal and thoroughly controlled circumstances and, therefore, substantially differ from real-world conditions (eg, restrictive selection of study sample and control of intervention delivery). Pragmatic study designs can determine the effect of an intervention under more realistic conditions by maximizing external validity (eg, broad and inclusive eligibility criteria and flexibility in intervention delivery) [20-23]. Studies are not strictly dichotomous in their design; instead, they are situated along the explanatory-pragmatic continuum [21,22,24]. Essentially, the challenge is to strike a balance between a highly effective program and whether it can be integrated into practice settings. mHealth interventions have the unique advantages of leveraging automation, data-informed decision-making, and other technological components that might aid in adherence to the core elements (eg, key ingredients or mechanism of change) while scaling out [25].

Existing systematic reviews of mHealth studies have broadly called for increased pragmatism [18,26,27]; however, only one research review has specifically explored the generalizability and applicability of app-based physical activity interventions [16]. However, the results were limited by the insufficient reporting of external validity factors within the included studies. Thus, the review authors were not able to determine the generalizability of the findings and recommended that future mHealth researchers better report all study characteristics [16]. Specific study design characteristics, such as the study sample’s demographics (eg, average age and gender) and the duration of the intervention, are important dimensions to evaluate when determining the generalizability of a study’s findings to the full population.

Given the continued growth of app-based physical activity interventions [14] and the lack of clarity surrounding the pragmatic nature of these approaches, we conducted a systematic review and meta-analysis of mHealth apps for physical activity promotion.

### Objective

Our primary aim was to analyze the degree to which these interventions reported the study characteristics necessary to inform generalizability and applicability and to assess the explanatory versus pragmatic nature of these studies. Our secondary aim was to explore the association between study design characteristics (eg, explanatory vs pragmatic, intervention duration, and participant demographics) and the observed effect sizes on participants’ physical activity.

## Methods

### Protocol and Registration

This review followed the PRISMA (Preferred Reporting Items for Systematic Reviews and Meta-Analyses) guidelines (Multimedia Appendix 1) [28,29].

### Search Strategy and Study Selection

We conducted a systematic search in 4 electronic databases on April 4, 2020: PubMed, Scopus, Web of Science, and PsycINFO. The search combined synonyms and keywords related to an app-based mHealth intervention for promoting physical activity (Table 1; Multimedia Appendix 2). We attempted to control for language bias by using a search strategy without language restriction (ie, no selective inclusion of trials published in English) [30]. In addition to these databases, the list of papers discussed by relevant systematic reviews [8,31-40] was examined to identify any further eligible studies.

**Table 1:** Search strategy used in PubMed on April 4, 2020.

Search category	Search term
mHealth^a^	mHealth OR mobile health OR m-health OR activity tracker OR fitness tracker OR wearable OR tablet OR personal digital assistant OR pda OR short message service OR sms OR text message OR android OR iphone OR iOS OR mobile phone OR cellphone OR cell phone OR cellular phone OR cellular telephone OR mobile telephone OR smart-phone OR smartphone OR mobile application OR mobile app
Physical activity	physical activity OR leisure activity OR active living OR exercise OR sport OR fitness OR motor activity OR sedentary behavior OR sedentary lifestyle OR sitting OR physical inactivity
Intervention	Intervention OR trial OR program
Study design	clinical trial OR controlled trial OR controlled study OR double blind OR RCT^b^ OR pragmatic trial OR practical trial OR PCT^c^ OR ecological trial OR dynamic trial OR real-world OR real world
Combined	mHealth AND Physical activity AND Intervention AND Study design

^a^mHealth: mobile health.

^b^RCT: randomized controlled trial.

^c^PCT: practical clinical trial.

The included studies were limited to app-based physical activity interventions that were published in a peer-reviewed journal between January 2012 and April 2020 that primarily targeted physical activity and at most one other behavioral outcome and that presented quantitative outcome data. We further restricted our review to studies that collected device-based physical activity measures, as opposed to self-reported measures because device-based measures are frequently observed to be more reliable [41,42] and the use of physical activity–monitoring devices has become more commonplace in the real world [43], demonstrating the feasibility, acceptability, and pragmatism of these intervention tools. A complete list of the eligibility criteria is presented in Table 2. We obtained additional data sources (when available) such as the study protocol, the CONSORT (Consolidated Standards of Reporting Trials) checklist, or any other publicly available information from the corresponding authors provided via an email invitation to assess the Reach, Effectiveness, Adoption, Implementation, Maintenance (RE-AIM) framework for internal and external validity factors [44,45] and the Pragmatic-Explanatory Continuum Indicator Summary-2 (PRECIS-2) tool for evaluating interventions’ pragmatism [24]. Specifically, this email contained a brief description of our study, and then asked, “In order to comprehensively evaluate the reporting of RE-AIM and PRECIS-2 criteria, we are also extracting data from study protocols and companion articles (eg, qualitative or quantitative methods measuring implementation). Would you be willing to help us by providing these additional resources?”

All records from the databases and supplementary searches were managed using the Microsoft EndNote X9 (Clarivate) reference manager software. After removing duplicates, we exported the records to Abstrackr (Brown University) for semiautomatic citation screening [46]. The relevance of the titles and abstracts was independently assessed by 2 authors (BP and JMH). Each eligible full text was independently reviewed by 2 researchers (SMH and MPB). Discrepancies were resolved through discussion between the screening authors. Any remaining conflicts were discussed among the other authors (CS, DE, KW, and BP) until consensus was reached.

**Table 2:** Eligibility criteria.

Data type	Eligibility criteria
Population	Participants of any age participating in physical activity programs in the context of health promotion or preventive care settings were included. Studies focusing on special populations (eg, pregnant women) or studies including participants with physical or psychological morbidities preventing them from participating in physical activity were excluded.
Intervention	Stand-alone mobile apps and web apps exclusively designed for mobile devices; multicomponent interventions (eg, supported through brief counseling sessions or paired with other mHealth^a^ technologies) were included as long as the app was the primary component to the intervention; interventions that targeted ≥2 health behaviors in addition to physical activity (eg, diet, sleep, and SB^b^) were excluded; apps solely used for data collection purposes or as an appointment reminder service only were not eligible.
Comparator	Active or inactive comparator arms were included; single-subject design trials were excluded.
Outcome	Device-based measures of physical activity.
Study design	RCTs^c^ and randomized ecologically valid research designs (ie, practical clinical trials, RCTs); randomized pilot and feasibility studies were included.

^a^mHealth: mobile health.

^b^SB: sedentary behavior.

^c^RCT: randomized controlled trial.

### Data Collection Process

#### General Study Characteristics

We adapted an existing extraction template [32] to collect and summarize the general study characteristics. Specifically, we collected information about the study setting and design, study population, intervention components, outcome measures, key findings, and statistical analyses performed (Multimedia Appendix 3). Two authors (BP and JMH) separately extracted additional quantitative data for the meta-analyses; discrepancies were resolved through discussion and consultation with a third author (SMH).

#### RE-AIM Evaluation and PRECIS-2 Assessment

We used the RE-AIM framework to describe the degree of reporting of study characteristics across 5 dimensions (ie, reach, effectiveness, adoption, implementation, and maintenance). The evaluation was assisted by a 31-item RE-AIM coding system used in a previous study [47]. We then applied the PRECIS-2 tool to compare the interventions with usual care and to identify the pragmatic versus explanatory nature of each study. Following the guidance of Loudon et al [24] and the PRECIS-2 toolkit published on the web, usual care was defined as the primary care that patients usually received for medical advice and treatment. The PRECIS-2 tool comprises 9 domains (ie, eligibility criteria, recruitment, setting, flexibility [delivery], flexibility [adherence], follow-up, primary outcome, and primary analysis), each of which is assigned a score from 1 to 5 (1 is *very explanatory* and 5 is *very pragmatic*) [24]. In accordance with previous research [47], mean scores of >3.5 were deemed *primarily pragmatic*. Values between 2.5 and 3.5 were considered *equally pragmatic and explanatory*, and scores <2.5 were rated as *primarily explanatory*.

Although both frameworks can be applied regardless of the study setting, additional modifications to these frameworks are recommended for a given setting [48]. Thus, we adapted the RE-AIM and PRECIS-2 coding sheets [49] for our setting (Multimedia Appendix 4 presents these adapted coding sheets). The final scoring by the study is presented in Multimedia Appendix 5.

### Quality Assessment

For each study, we also assessed quality of the study using the revised Cochrane risk-of-bias (RoB 2.0) tool for randomized controlled trials [50]. Two authors (BP and JMH) independently performed these assessments, and any disagreements were resolved through discussion with a third author (SMH, DE, and MPB). The studies were classified as having a *low risk of bias* if all the 5 assessment domains were considered low risk. Otherwise, the studies were classified as having s*ome concerns* when concerns were raised in at least 1 of the 5 domains, or they were classified as having *high risk of bias* when at least one of the domains was judged to be at high risk. These categories were drawn from the original Cochrane RoB 2.0 tool [50].

### Statistical Analyses

We used counts and percentages to summarize the general study characteristics and RE-AIM and PRECIS-2 scores for each study.

Meta-analyses were performed by using *meta* commands in Stata 16 (StataCorp) [51]. We used the standardized average treatment effect in each study’s primary app- or device-based physical activity outcome (ie, minutes of moderate to vigorous physical activity or step count) to compare treatment effects across studies with different outcomes. The standardized average treatment effect (or Cohen *d*) was calculated as the difference in the mean change in primary physical activity outcome between the intervention group and the control group divided by the pooled SD of the physical activity outcome in both the intervention and control groups, with a priori interpretations [52] of trivial (<0.2), small (0.2-0.5), moderate (0.5-0.8), and large (>0.8) effects.

In addition, we tested for heterogeneous treatment effects using random-effects models estimated through restricted maximum likelihood. All the following moderating variables were log transformed to better compare the effect sizes: baseline physical activity, sample size, participants’ age, participants’ gender, intervention duration, RoB score, RE-AIM score, and PRECIS-2 score. Bubble plots were used to graphically examine the relationships between treatment effect size and the continuous moderating variables.

We assessed the statistical significance of treatment effect heterogeneity by using Cochran Q test and calculating the Higgins *I*^²^ statistic [53]. The following thresholds for the interpretation of the *I*^²^ statistic were used:0%-40%, 30%-60%, 50%-90%, or 75%-100%; these were interpreted as *not likely important*, *moderate*, *substantial*, and *considerable* heterogeneity, respectively [53].

Finally, the combined impact of small-study effects and publication bias was assessed by using the trim-and-fill method and performing the Egger test using the *metafor* package [54] in R (version 3.6.3; R Foundation for Statistical Computing) [55]. The results are reported with 95% CI, and a *P* value of <.05 was considered statistically significant.

## Results

### Study Selection

**Figure 1:**
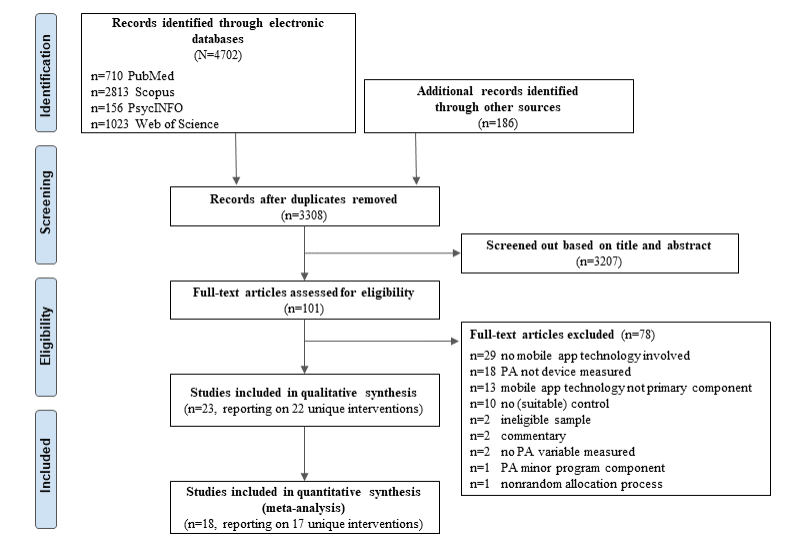
Flowchart of study selection. PA: physical activity.

The search yielded 3308 unique studies after duplicates were removed. Of the 3308 studies, we screened 3207 (96.95%) studies based on title and abstract, leaving 101 (3.05%) potentially relevant studies. After additional content reviews, 23 studies reporting 22 unique interventions met the eligibility criteria for inclusion in the RE-AIM and PRECIS-2 analyses. We emailed the corresponding authors of all 23 studies to request additional study information. We received responses from 52% (12/23) of the studies, and these responses either contained more information on the study (7/12, 58%) or simply stated that there was no additional information available (5/12, 42%). In total, only 74% (17/23) of these studies presented sufficient quantitative detail for inclusion in the meta-analyses. The detailed study selection process is visualized in the PRISMA flowchart (Figure 1).

### Study Characteristics

All interventions were published in English between 2012 and 2020 and were conducted in 10 countries, with most interventions (10/22, 45%) having based in the United States [56-65]. Of the 22 interventions, 21 (95%) used a randomized controlled trial design, of which 19 (90%) interventions randomized participants on an individual level and 3 (14%) interventions were randomized in clusters [66-68]. One study explicitly used a pragmatic study design [69]; 6 studies identified their trials as pilot studies [56,61,62,64,70,71], and 1 was classified as a feasibility study [72]. One study used a factorial design between multiple intervention components as part of a multiphase optimization strategy [57]. An overview of these study characteristics for each study is presented in detail in Multimedia Appendix 6.

A total of 3555 participants were included across all 22 interventions, with sample sizes ranging from 27 to 833 (mean 161.6, SD 193.9, median 93) participants. All studies were conducted in a health promotion or preventive care setting, and the most common study settings were the local community (10/22, 45%), a university or other type of school (7/22, 32%), or a clinical care setting (3/22, 14%). In addition, 10 interventions exclusively targeted insufficiently active individuals. Study populations varied in age and gender, with mean ages ranging from 10.6 to 61.5 (mean 39.6, SD 6.5) years, and the proportion of males included across all studies was 42.8% (1521/3555). Moreover, 2 studies exclusively targeted men, and 2 studies included women only.

Intervention length varied from 2 weeks to 6 months (mean 60.9, SD 34.9 days). The primary app- or device-based physical activity outcomes differed between interventions, with most interventions (17/22, 77%) using activity monitors or fitness trackers and the rest (5/22, 23%) using app-based accelerometry measures. All studies reported either moderate to vigorous physical activity, daily steps, or both measures. The comparator groups received either no intervention (10/22, 45%); a minimal intervention such as generic physical activity information (6/22, 27%); a basic app version targeting physical activity (3/22, 14%); a control app unrelated to physical activity (1/22, 5%); or a wearable activity monitor with access to its corresponding generic tracking app (2/22, 9%).

A total of 27% (6/22) of studies targeted physical activity, and 5% (1/22) of studies targeted additional health behavior outcome (ie, diet or sedentary behavior). With regard to the physical activity intervention strategies used in all studies, 27% (6/22) of studies provided brief in-person expert consultations (eg, goal setting or generic physical activity information), and 5% (1/22) of interventions included weekly telephone counseling. Most studies (19/22, 83%) also used emails and text messages as physical activity reminders or to provide participants with an activity summary.

The interventions’ apps varied greatly between the studies and consisted of both commercial products and apps designed solely for research purposes. The apps included features such as physical activity tracking and self-monitoring, feedback, goal setting, social interaction, and gamification features (Multimedia Appendix 6 provides the full list of app features by intervention).

### RoB Assessment

Table 3 shows the RoB in the included studies. Overall, 17% (4/23) of studies showed a *low risk*; 43% (10/23) of studies raised *some concerns*; and 39% (9/23) of studies were rated *high risk*. A lack of balance across randomized study groups in terms of baseline physical activity and gender contributed to a *high risk of bias* classification for 3 studies, and 2 other studies were considered to have a *high risk of bias* for deviating from their intended intervention design, which the authors attributed to a lack of participant engagement with the intervention’s physical activity app and the intended intervention. In addition, most studies (14/22, 64%) did not provide enough information to determine whether the data were analyzed according to their prespecified data analysis plan, which resulted in them being classified as having *some concerns*.

**Table 3:** Risk-of-bias (RoB) assessment based on the revised Cochrane RoB tool for randomized trials (RoB 2.0).^a^

Study, year	Randomization bias^b^	Deviation bias^c^	Missing data bias^d^	Measurement bias^e^	Selection bias^f^	Overall
Direito et al [69], 2015	+	+	+	+	+	+
Edney et al [66], 2020	+	+	+	+	+	+
Fanning et al [57], 2017	?	?	?	+	?	−
Fukuoka et al [58], 2019	+	+	+	+	+	+
Garcia-Ortiz et al [73], 2018	+	?	?	+	+	?
Garde et al [74], 2018	+	?	?	+	?	−
Glynn et al [75], 2014	?	+	+	+	+	?
Gremaud et al [59], 2018	+	?	+	+	?	?
Harries et al [76], 2016	?	?	+	+	?	−
Hurkmans et al [77], 2018	−	?	+	+	+	−
King et al [60], 2016	+	+	+	+	?	?
Kitagawa et al [70], 2020	?	+	+	+	?	?
Leinonen et al [72], 2017	+	−	−	+	+	−
Lyons et al [61], 2017	+	+	+	+	+	+
Martin et al [56], 2015	+	+	+	+	?	?
Pope and Gao [62], 2020	−	?	+	+	?	−
Recio-Rodriguez et al [78], 2016	+	?	?	+	+	?
Robertson et al [67], 2018	+	−	−	+	?	−
Schade et al [63], 2020	?	?	−	+	?	−
Simons et al [68], 2018	−	?	+	+	?	−
Walsh et al [71], 2016	+	+	+	+	?	?
Zhang, and Jemmott [64], 2019	+	?	+	+	?	?
Zhou et al [65], 2018	+	+	+	+	?	?

^a^+ = low risk of bias; ?=some concerns; −=high risk of bias.

^b^Bias arising from the randomization process.

^c^Bias because of deviations from the intended intervention.

^d^Bias because of missing outcome data.

^e^Bias because of measurement tools used to collect outcome data.

^f^Bias in selection of the reported result.

### RE-AIM Evaluation

#### Overview

The overall rating of sufficiently reported individual RE-AIM items across all interventions was 18% (5.64/31, SD 2.30%; Table 4). Reporting ranged from 2 to 11 of the 31 RE-AIM items. The most commonly reported items were those in the Effectiveness (2.6/5, 52%) and Reach (1.8/4, 45%) dimensions. Reported data within the Maintenance categories were observed in only 12% (1.1/9) of the interventions, and the reporting of items in the Adoption and the Implementation dimensions were found in 4% (0.3/8) and 10% (0.5/5) of the interventions, respectively. A summary of the key findings of the factors within each dimension is presented in the subsequent section.

**Table 4:** Inclusion of Reach, Effectiveness, Adoption, Implementation, Maintenance (RE-AIM) items across all interventions (N=22).^a,b^

RE-AIM dimension and items			Values, n (%)
**Reach (44.3%)**			
	Exclusion criteria	17 (77)	
	Participation rate	16 (73)	
	Representativeness	6 (27)	
	Use of qualitative methods to understand reach and recruitment	0 (0)	
**Effectiveness (52.7%)**			
	Measure of primary outcome	22 (100)	
	Measure of broader outcomes (ie, QoL^c^, negative outcomes)	11 (50)	
	Measure of robustness across subgroups	4 (18)	
	Measure of short-term attrition	14 (64)	
	Use of qualitative methods or data to understand outcomes	7 (32)	
**Adoption-setting (3.4%)**			
	Setting exclusions	2 (9)	
	Setting adoption rate	1 (4)	
	Setting representativeness	0 (0)	
	Use of qualitative methods to understand adoption at setting level	0 (0)	
**Adoption-staff (0%)**			
	Staff exclusions	0 (0)	
	Staff participation rate	0 (0)	
	Staff representativeness	0 (0)	
	Use of qualitative methods to understand staff participation	0 (0)	
**Implementation (10%)**			
	Delivered as intended	5 (23)	
	Adaptations to intervention	4 (18)	
	Cost of intervention (time or money)	0 (0)	
	Consistency of implementation across staff or time or settings subgroups	2 (9)	
	Use of qualitative methods to understand implementation	0 (0)	
**Maintenance-individual (9%)**			
	Measure of primary outcome at ≥6-mo follow-up	3 (14)	
	Measure of broader outcomes (ie, QoL, negative outcomes) at follow-up	2 (9)	
	Measure of long-term robustness across subgroups	2 (9)	
	Measure of long-term attrition	3 (14)	
	Use of qualitative methods to understand long-term effects	0 (0)	
**Maintenance-setting (3.4%)**			
	Program ongoing (≥6-mo poststudy funding)	1 (4)	
	Long-term program adaptations	2 (9.1)	
	Some discussion of sustainability of business model	0 (0)	
	Use of qualitative methods to understand setting-level institutionalization	0 (0)	

^a^The table formatting was adapted from Burke et al [47].

^b^Overall RE-AIM was 18.2%.

^c^QoL: quality of life.

#### Reach

*Exclusion criteria* commonly included health contraindications for participating in physical activity or comprised mHealth-specific requirements (eg, specifications around technical devices). Most studies provided accurate information (ie, either n and valid denominator or percentage) on the *participation rate* (16/22, 73%) [56-60,62-66,68,69,71,72,75,78]; however, only a few (3/22, 14%) reported the sample size in relation to the total number exposed to recruitment [65,68,72], and the remaining trials reported only on the relation of the sample size to potentially eligible participants [56-60,62-64,66,69,71,75,78]. A few interventions (6/22, 27%) adequately reported the *representativeness* of the study sample. One intervention compared their sample to eligible individuals who declined participation [72], and 5 compared their sample and their target audience [58,62,66,70,71]. Comparisons were made on physical activity variables and anthropometry and fitness measures.

#### Effectiveness

All studies (23/23, 100%) reported a *measure of primary outcome* related to physical activity (per review eligibility criteria), and half of the interventions (11/22, 50%) addressed a *measure of broader outcomes* [56,57,60,61,65-67,69,70,72,75]. Moreover, 45% (10/22) of studies compared their physical activity–related findings to a public health goal (ie, physical activity guidelines) [56,58,62-64,71,74-76]; however, only a few studies (4/22, 18%) analyzed the *robustness across study subgroups* (eg, gender and age groups) [56,58,64,76]. Potential explanations for physical activity–related findings were explored using *qualitative research methods* in several interventions (7/22, 32%) [57,62,67-69,72,76].

#### Adoption

Both nonresearch and research staff participation were considered, and more participation of either nonresearch or research staff would result in a study being less pragmatic if it exceeded the usual standard of care. However, no items were reported within the dimension “Adoption-staff.” Regarding “Adoption-setting,” 2 studies specified *setting* *exclusions* (eg, unqualified staff and irregular physical education classes) [67,68]. One intervention presented a valid *setting adoption rate* [68].

#### Implementation

The *delivered as intended* and the *adaptations to intervention* items were infrequently addressed and were mainly of technical nature (eg, app bug or app appearance). None of the studies sufficiently reported the *cost of* intervention, meaning that costs were not addressed across all levels of the intervention or were not detailed enough (eg, app development, technical equipment, and support). The *consistency of implementation* was outlined in 2 trials (eg, fidelity checks) [58,78].

#### Maintenance

A few interventions (3/22, 14%) assessed a *≥6-month follow-up* measure; 2 studies reported a 6-month follow-up phase [58,66]; 1 implemented a 9-month follow-up measure [73]; and all these studies reported an accurate *long-term attrition rate*. Two studies analyzed the *long-term robustness* (eg, age and weight status) [58,73]. A *measure of broader outcomes* was reported in 2 interventions, assessing the quality of life using the 12-Item Short-Form Health Survey [58,66].

Items within the Maintenance-Setting dimension were only addressed by 3 interventions, including potential *long-term adaptations* (eg, implementing an educational app component) [56,72,74]. The sustainability of the program in the RE-AIM context was not discussed at all.

### PRECIS-2 Assessment

The overall PRECIS-2 score across all interventions was 2.93/5 (SD 0.54). Of the 22 assessed interventions, 14 (64%) interventions were categorized as *equally pragmatic* *and explanatory* (range 2.56-3.44) [57,59,62–67,69–71,73,74,76]; 5 (23%) studies were identified as being *primarily explanatory* (range 2.00-2.44) [58,60,61,68,77]; and 3 (14%) studies were *primarily pragmatic* (range 3.56-4.44) [56,72,74].

The most pragmatic dimension across all interventions was *flexibility (adherence)*, with an average score of 3.73 (SD 0.92), as demonstrated by letting the participants use the app at their convenience or lacking any measures to improve adherence. *Follow-up*, *organization*, and *flexibility (delivery)* appeared to be more explanatory, with means of 2.18 (SD 0.75), 2.36 (SD 1.07), and 2.41 (SD 0.72), respectively. For example, delivery flexibility was considered more explanatory based on in-person requirements, clinician oversight, or specific app use or compliance requirements. Domains considered equally explanatory and pragmatic were *eligibility criteria*, *recruitment*, *setting*, *primary outcome*, and *primary analysis* (range 2.95-3.45). Overall, the studies in this review were equally pragmatic and explanatory in terms of the eligibility criteria.

### Meta-analysis

#### Overall Treatment Effect

**Figure 2:**
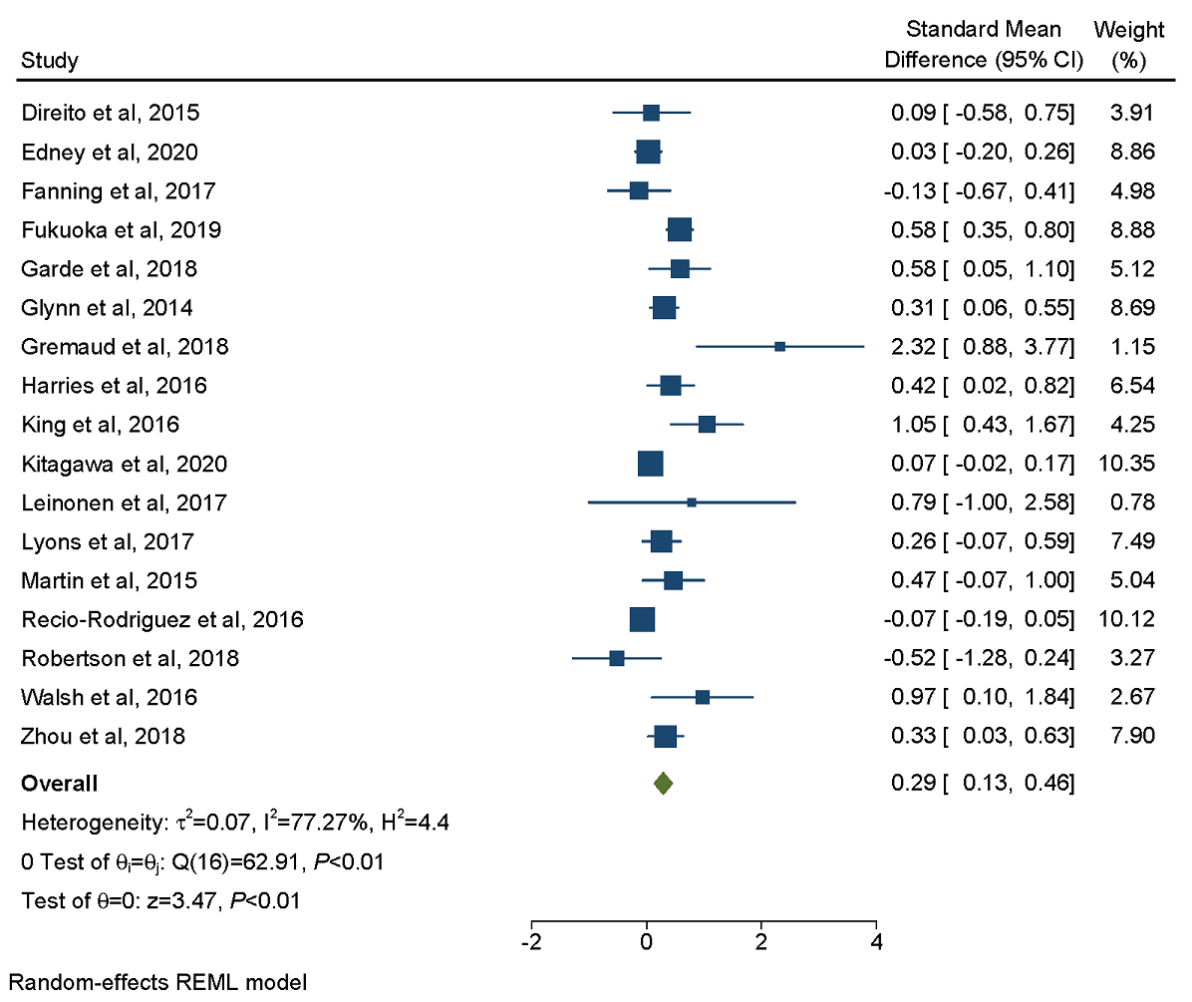
Forest plot of standardized treatment effects on physical activity with studies weighted by the inverse of the SE of the estimated treatment effect. REML: restricted maximum likelihood.

Data from only 17 interventions were extracted for this meta-analysis because 5 interventions did not present complete outcome data (ie, they did not report SE or 95% CI). Overall, these 17 mHealth interventions significantly improved the participants’ physical activity (Cohen *d*=0.29, 95% CI 0.13-0.46; Figure 2).

#### Meta-regression Analyses

Meta-regression analyses revealed a statistically significant negative association between the standardized treatment effect and the study’s sample size (*P*=.01), PRECIS-2 score (*P*<.001), and study participants’ baseline physical activity (*P*<.001; Table 5), that is, a larger sample size, higher PRECIS-2 score (ie, more pragmatic), and higher observed baseline physical activity levels were all associated with smaller treatment effect sizes on participants’ physical activity. None of the other covariate measures (ie, intervention duration, participants’ age, participants’ gender, and RE-AIM score) were significantly related to changes in participants’ physical activity.

**Figure 3:**
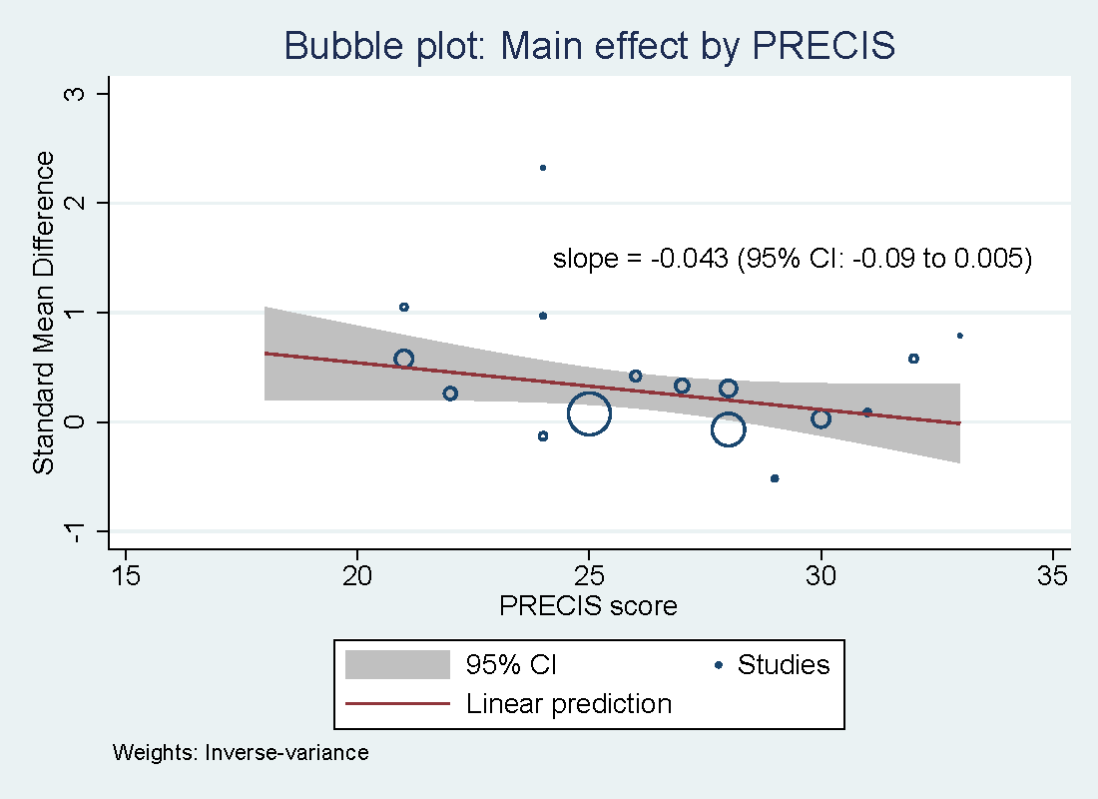
Bubble plot of standardized treatment effect on Pragmatic-Explanatory Continuum Indicator Summary-2 (PRECIS-2) score (a single outlier was removed).

To graphically depict the interaction between the treatment effect size and the continuous measure of a study’s PRECIS-2 score, we created a bubble plot with studies represented by circles sized by the inverse of the SE of the estimated treatment effect (Figure 3). The plot also shows the weighted linear relationship between these study characteristics and the 95% CI for this estimated relationship.

**Table 5:** Meta-regression results showing the interaction between study characteristics and the standardized treatment effect on physical activity.^a^

Covariate	Standardized mean difference (95% CI)	*P* value
Log (intervention duration [days])	0.0171 (−0.0338 to 0.0680)	.51
Log (participant mean age [years])	−0.00296 (−0.224 to 0.218)	.98
Log (sample size)	−0.0616^b^ (−0.111 to −0.0123)	.01
Log (percentage male)	−0.0615 (−0.266 to 0.143)	.56
Log (baseline step count)	−0.420^c^ (−0.637 to −0.202)	<.001
Log (baseline MVPA^d^ [minutes])	−0.199^c^ (−0.288 to −0.109)	<.001
Log (PRECIS-2^e^ score)	−0.805^f^ (−1.361 to −0.249)	<.001
Log (RE-AIM^g^ score)	−0.0277 (−0.177 to 0.122)	.72
Log (risk-of-bias score)	−0.199 (−0.406 to 0.0690)	.06

^a^All covariates were log transformed; therefore, the coefficients measure the associated change in the standardized treatment effect size from a 1% increase in the indicated variable.

^b^*P*<.05.

^c^*P*<.001.

^d^MVPA: moderate to vigorous physical activity.

^e^RE-AIM: Reach, Effectiveness, Adoption, Implementation, Maintenance.

^f^*P*<.01.

^g^PRECIS-2: Pragmatic-Explanatory Continuum Indicator Summary-2.

#### Overall Treatment Effect Heterogeneity

The meta-analysis showed considerable heterogeneity between the studies, with an *I*^2^ value of 77.27%. The *I*^2^ value represents the estimated percentage of variability in the results because of heterogeneity rather than chance [53]. Cochran Q test for treatment effect heterogeneity across these studies was Q_16_=62.91, which demonstrates a statistically significant degree of heterogeneity (*P*<.001).

#### Analysis of Publication Bias and Small-Study Effects

**Figure 4:**
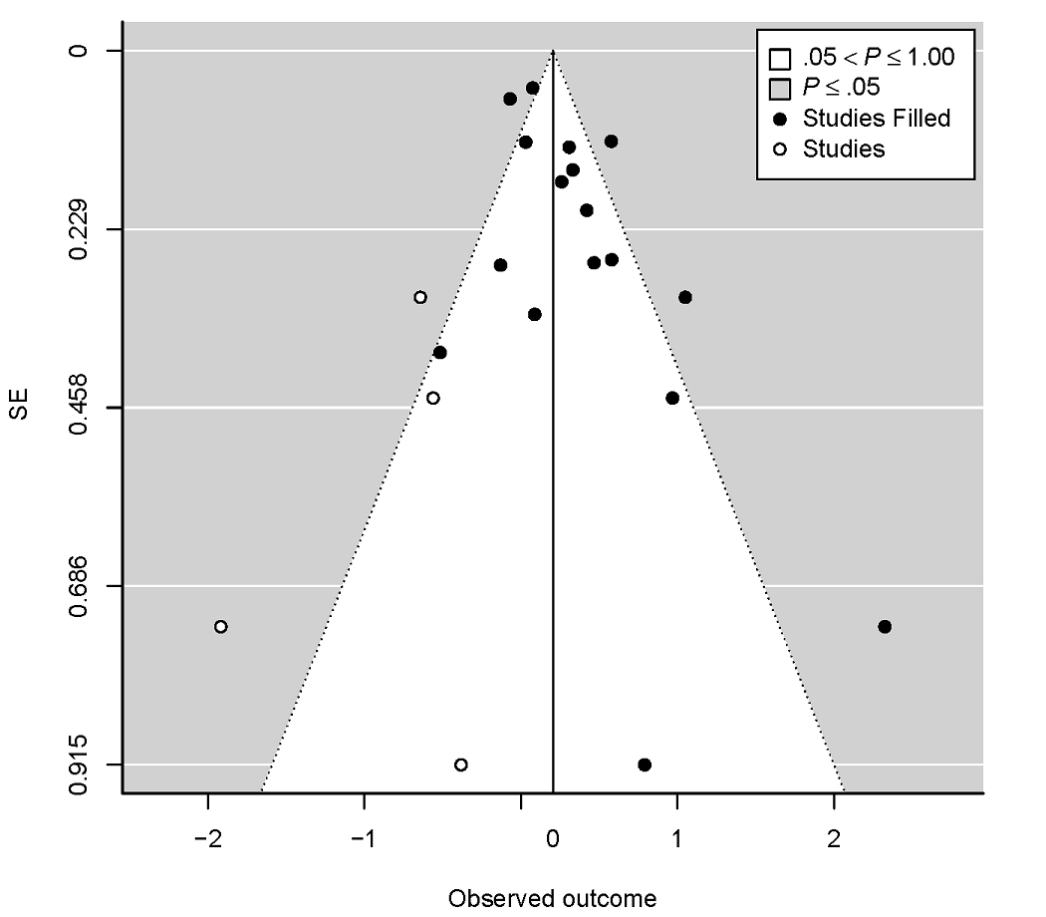
Trim-and-fill funnel plot for included studies in this meta-analysis.

We used the trim-and-fill method to explore the potential impact of publication bias in this literature, which estimated the number of studies missing from this literature to be 4 (SE 2.80; Figure 4). After imputing these missing studies, the overall standardized treatment effect size was slightly reduced from 0.29 (95% CI 0.13-0.46) to 0.20 (95% CI 0.01-0.40) but remained statistically significant. A high *I*^²^ value of 83.8% indicated that the heterogeneity between studies remained at a considerable level after imputing these potentially missing studies. We then carried out the Egger test for small-study effects, which reached statistical significance under most specifications (Multimedia Appendix 7).

## Discussion

### Principal Findings

Among recent studies using app-based interventions to promote physical activity, we observed a significant degree of underreporting on several RE-AIM dimensions, which limits researchers’ and policy makers’ ability to assess the generalizability of the research results. In addition, the interventions in this literature, in general, had more explanatory rather than pragmatic designs, which further limits our ability to forecast how successful these interventions would be in promoting physical activity if implemented among the general population. Finally, the aggregate study results showed a small but significant improvement in participants’ physical activity. However, treatment effect sizes varied according to the PRECIS-2 classification, where the more pragmatic trials produced smaller treatment effects on physical activity. Taken together, these findings suggest that app-based physical activity interventions would have limited efficacy in promoting physical activity if more widely scaled and adopted among the general population, suggesting that more pragmatic study designs are needed to increase the transferability from research to practice. The recommendations provided by Blackman et al [16] should be used more widely by researchers in this literature when designing and reporting study findings.

### RE-AIM Evaluation and PRECIS-2 Assessment

#### RE-AIM Evaluation

Our findings build on a prior review of mHealth physical activity interventions that also observed a lack of reporting on study characteristics and research findings in this literature [16]. Without sufficient information on these important study dimensions, the previous review was unable to determine the generalizability of the research findings at that time. Our more detailed and updated review demonstrates that only small improvements in transparency and the reporting of study characteristics have been achieved in mHealth physical activity research since then.

Our finding that recent mHealth physical activity studies lack transparency builds on similar observations reported in reviews of physical activity interventions using both mHealth and other intervention tools [47,49,79]. Specifically, the review by Blackman et al [16] on the mHealth physical activity literature found that few studies reported on the maintenance of intervention effects and the degree of implementation fidelity. In addition, the review by Harden et al [49] on group-based physical activity interventions showed that external validity factors were consistently underreported, and the review by Burke et al [47] on physical activity interventions for adults with spinal cord injuries found that several items within the Adoption and Maintenance dimensions of RE-AIM were not reported in any study, limiting the generalizability of these studies.

Two specific areas of underreporting in the mHealth physical activity studies that we reviewed were in the Adoption and Maintenance dimensions. The lack of reported information on the ability of health care providers to adopt these app-based physical activity intervention tool or tools significantly limits the willingness of clinicians and organizations to implement these new intervention approaches [16,47,49]. More pragmatic study designs with greater reporting of the Adoption and Maintenance dimensions are needed to increase the implementation of these mHealth tools in real-world settings. In addition, none of the studies reported sufficiently the cost of the intervention (in terms of either time or money), making it difficult to assess the benefit versus cost of these tools. Rubin et al [80] noted that prior complications experienced when integrating mHealth technologies into clinical practice have likely increased providers’ hesitancy to adopt new mHealth strategies. Therefore, we believe that increased reporting of interventions’ organizational requirements and costs (eg, required staff qualifications, equipment for delivery and analysis, cost of acquiring the intervention tools, and maintenance) would increase the applicability of this research.

#### PRECIS-2 Assessment

With regard to the PRECIS-2 results, our domain-specific assessments suggest that these recent studies testing app-based physical activity interventions tend to be primarily explanatory in nature. To combat a lack of app engagement, many studies used additional text messages or email reminders to reengage participants with the interventions’ app. These additional intervention components lowered our assessment of pragmatism, as it is not clear how well these methods can be widely implemented in usual care practices. Although the apps were considered relatively pragmatic in terms of their ease of accessibility, many studies also used frequent assessments and in-person intervention components or brought participants into research-specific facilities, limiting their overall level of pragmatism. Importantly, the use of device-based physical activity measures did not influence PRECIS-2 scores, as these devices are increasingly available and integrated into usual care.

#### Challenges and Adaptations of RE-AIM and PRECIS-2

To address the underreporting of study characteristics, we combined the main intervention report with additional documents available on the web but found few additional study details through these additional sources; thus, we want to emphasize that a greater “consensus around the use of frameworks and checklists across scientific fields and journals” is still needed [47]. We also expanded the original RE-AIM framework to include a third scoring category (*inadequately/insufficiently reported)* but found that assessing this added nuance in reporting adds substantially more work to the review process. Therefore, we refer readers and future reviewers to the ongoing creation of domain-specific review tools [81], which will hopefully be able to strike a better balance between researcher burden and improved accuracy.

### Meta-analysis

Overall, these recent app-based physical activity interventions produced small but significant increases in participants’ physical activity. This finding is in line with the results of previous reviews that also found a small and significant effect of app-based interventions on promoting physical activity [31-33,82]. In addition, our meta-analysis found that study effect sizes were not significantly different between interventions with durations longer than 8 weeks compared with those with shorter durations (Multimedia Appendix 7), which suggests that duration alone is not a predictor of a successful physical activity intervention and that additional approaches and intervention tools are still needed to change and maintain physical activity increases. Finally, a few of these studies were able to demonstrate, or even assess, the maintenance of physical activity after the interventions were withdrawn. This finding emphasizes the need for an improved understanding of physical activity habits and the maintenance of initial behavioral change.

The lack of evidence for an optimal physical activity intervention duration and for the maintenance of physical activity increases has been noted in previous reviews of the mHealth literature. Contrary to our findings, Romeo et al [32] found that the most effective physical activity interventions had durations longer than 8 weeks. In addition, the review by Schoeppe et al [33] on app-based health interventions showed the greatest effects among interventions for up to 3 months in duration. The discrepancy between our results and those of previous studies demonstrates the need for more evidence on the optimal intervention duration. With regard to the maintenance of intervention effects, a recent systematic review by Pradal-Cano et al [82] described the need for longer-term studies to observe the maintenance of intervention effects after the intervention components are withdrawn. Among the studies reviewed by Pradal-Cano et al [58,66,73], only 3 reported on the maintenance of intervention effects at least 6 months after the intervention was withdrawn, and there were fixed findings on maintenance among these studies.

### Strengths and Limitations

Adapting 2 complementary implementation science tools to better understand the generalizability and applicability of app-based physical activity intervention findings is a key strength of this review; however, this review is not without limitations. First, our literature search identified a relatively small number of unique interventions, which limited the power of our statistical methods. Second, the included studies significantly varied in terms of design parameters (eg, sampling frame and intervention components) and methodological parameters (eg, outcome measures). This considerable heterogeneity was identified in the meta-analyses and indicated the difficulties in synthesizing this literature. Although we focused only on app-based physical activity interventions, most interventions incorporated additional intervention components, precluding us from isolating the individual effect of the app on physical activity. Third, our literature search was performed in 2020, and more studies using mobile apps to increase physical activity have been published since then [83-85]. Although it is beyond the scope of this paper to incorporate these studies into our complete analyses, they all provide additional evidence that mobile apps can improve physical activity. In addition, one of these recent studies reported long-term behavioral maintenance outcomes [85], which is an important step in the mHealth app literature. Another important limitation is that most included studies targeted adults (19/22, 86%), which limits the generalizability of our findings to physical activity interventions among younger and older populations. Fourth, the significant degree of underreporting of study characteristics limited our ability to assess treatment moderation by individual RE-AIM dimensions, which is an important area for future research. Finally, our statistical analyses indicated the presence of a publication bias, potentially compromising the robustness of our findings. However, subsequent trim-and-fill analyses suggested that the overall treatment effect was only slightly reduced when attempting to account for these missing studies.

### Conclusions

This review highlights important limitations in the mHealth literature that uses app-based interventions to promote physical activity. Specifically, studies continue to underreport several key study characteristics that are necessary to determine the generalizability and scalability of these intervention approaches. Importantly, more pragmatic study designs are needed to help researchers and policy makers confidently implement app-based tools in standard care practice. In addition, studies with different intervention durations were equally effective in increasing physical activity, suggesting that additional intervention methods and approaches are necessary to improve the maintenance and growth of initial physical activity improvements.

## Appendix

Multimedia Appendix 1 PRISMA (Preferred Reporting Items for Systematic Reviews and Meta-Analyses) guidelines.

Multimedia Appendix 2 Search strategy for all electronic databases.

Multimedia Appendix 3 Data extraction form (general study characteristics).

Multimedia Appendix 4 Combined coding sheet (adapted Reach, Effectiveness, Adoption, Implementation, Maintenance [RE-AIM] and Pragmatic-Explanatory Continuum Indicator Summary-2 [PRECIS-2]).

Multimedia Appendix 5 Reach, Effectiveness, Adoption, Implementation, Maintenance (RE-AIM) and Pragmatic-Explanatory Continuum Indicator Summary-2 (PRECIS-2) scoring.

Multimedia Appendix 6 Extracted study characteristics.

Multimedia Appendix 7 Additional meta-analysis results.

## Abbreviations

CONSORT: Consolidated Standards of Reporting Trials
mHealth: mobile health
PRECIS-2: Pragmatic-Explanatory Continuum Indicator Summary-2
PRISMA: Preferred Reporting Items for Systematic Reviews and Meta-Analyses
RE-AIM: Reach, Effectiveness, Adoption, Implementation, Maintenance
RoB: risk-of-bias
