# Supplementary material for: Assessing the Pragmatic Nature of Mobile Health Interventions Promoting Physical Activity: Systematic Review and Meta-analysis
Source: JMIR Mhealth Uhealth. 2023 May 4;11:e43162. doi: 10.2196/43162 (PMC10196895; doi:10.2196/43162)
Supplement: Multimedia Appendix 2 [file mhealth_v11i1e43162_app2.pdf]

Search Strategy for all Electronic Databases

A systematic search was conducted in four electronic databases on April 4, 2020: PubMed, Scopus, Web of Science, and PsycINFO. The search results in each database were limited to the year of publication from 2012 to present.

Pubmed:

(((((((((((((((((((((((((((mHealth[Title/Abstract]) OR (mobile health[Title/Abstract])) OR (m-health[Title/Abstract])) OR (activity tracker[Title/Abstract])) OR (fitness tracker[Title/Abstract])) OR (wearable[Title/Abstract])) OR (tablet[Title/Abstract])) OR (personal digital assistant[Title/Abstract])) OR (pda[Title/Abstract])) OR (short message service[Title/Abstract])) OR (sms[Title/Abstract])) OR (text message[Title/Abstract])) OR (android[Title/Abstract])) OR (iphone[Title/Abstract])) OR (iOS[Title/Abstract])) OR (mobile phone[Title/Abstract])) OR (cellphone[Title/Abstract])) OR (cell phone[Title/Abstract])) OR (cellular phone[Title/Abstract])) OR (cellular telephone[Title/Abstract])) OR (mobile telephone[Title/Abstract])) OR (smart-phone[Title/Abstract])) OR (smartphone[Title/Abstract])) OR (mobile application[Title/Abstract])) OR (mobile app[Title/Abstract])) AND ((((((((physical activity[Title/Abstract]) OR (leisure activity[Title/Abstract])) OR (active living[Title/Abstract])) OR (exercise[Title/Abstract])) OR (sport[Title/Abstract])) OR (fitness[Title/Abstract])) OR (motor activity[Title/Abstract])) OR (((((sedentary behavior[Title/Abstract]) OR (sedentary behaviour[Title/Abstract])) OR (sedentary lifestyle[Title/Abstract])) OR (sitting[Title/Abstract])) OR (physical inactivity[Title/Abstract])))) AND (((intervention[Title/Abstract]) OR (trial[Title/Abstract])) OR (program[Title/Abstract]))) AND ((((((clinical trial[Title/Abstract]) OR (controlled trial[Title/Abstract])) OR (controlled study[Title/Abstract])) OR (double blind[Title/Abstract])) OR (RCT[Title/Abstract])) OR (((((((pragmatic trial[Title/Abstract]) OR (practical trial[Title/Abstract])) OR (PCT[Title/Abstract])) OR (ecological trial[Title/Abstract])) OR (dynamic trial[Title/Abstract])) OR (real-world[Title/Abstract])) OR (real world[Title/Abstract])))

PsycINFO:

(TI,AB,IF(mhealth) OR TI,AB,IF(m-health) OR TI,AB,IF("mobile health") OR TI,AB,IF(iOS) OR TI,AB,IF("activity tracker") OR TI,AB,IF("personal digital assistant") OR TI,AB,IF(tablet) OR TI,AB,IF(PDA) OR TI,AB,IF("short message service") OR TI,AB,IF(SMS) OR TI,AB,IF("text message") OR TI,AB,IF(android) OR TI,AB,IF(iphone) OR TI,AB,IF("mobile telephone") OR TI,AB,IF("mobile phone") OR TI,AB,IF("cell phone") OR TI,AB,IF(cellphone) OR TI,AB,IF(smartphone) OR TI,AB,IF(smart-phone) OR TI,AB,IF("mobile device") OR TI,AB,IF(wearable) OR TI,AB,IF("mobile app") OR TI,AB,IF("mobile application") OR TI,AB,IF("cellular phone") OR TI,AB,IF("cellular telephone")) AND ((TI,AB,IF("physical Activity") OR TI,AB,IF(fitness) OR TI,AB,IF(sport) OR OR TI,AB,IF(exercise) OR TI,AB,IF("active living") OR TI,AB,IF("leisure activity") OR TI,AB,IF("motor activity")) OR (TI,AB,IF("physical inactivity") OR TI,AB,IF("sedentary behavior") OR TI,AB,IF("sedentary behaviour") OR TI,AB,IF(sitting) OR TI,AB,IF("sedentary lifestyle"))) AND (TI,AB,IF(intervention) OR TI,AB,IF(trial) OR TI,AB,IF(program)) AND ((TI,AB,IF("clinical trial") OR TI,AB,IF("controlled trial") OR TI,AB,IF("controlled study") OR TI,AB,IF(RCT) OR TI,AB,IF("double blind")) OR (TI,AB,IF("pragmatic trial") OR TI,AB,IF("practical trial") OR TI,AB,IF(PCT) OR

TI,AB,IF("ecological trial") OR TI,AB,IF("dynamic trial") OR TI,AB,IF("real-world") OR TI,AB,IF("real world")))

Scopus:

(TITLE-ABS-KEY (mhealth OR m-health OR ios OR "mobile health" OR "activity tracker" OR wearable OR tablet OR "personal digital assistant" OR pda OR "short message service" OR sms OR "text message" OR android OR iphone OR cellphone OR "mobile telephone" OR "cell phone" OR smart-phone OR smartphone OR "mobile device" OR "mobile phone" OR "mobile app" OR "mobile application" OR "cellular phone" OR "cellular telephone")) AND ((TITLE-ABS-KEY ("physical inactivity" OR "sedentary behavior" OR "sedentary behaviour" OR sitting OR "sedentary lifestyle")) OR (TITLE-ABS-KEY ("physical activity" OR fitness OR sport OR exercise OR "active living" OR "leisure activity" OR "motor activity"))) AND (TITLE-ABS-KEY (intervention OR trial OR program)) AND ((TITLE-ABS-KEY ("clinical trial" OR "controlled trial" OR "controlled study" OR rct OR "double blind")) OR (TITLE-ABS-KEY ("pragmatic trial" OR "practical trial" OR pct OR "ecological trial" OR "dynamic trial" OR “real-world” OR “real world”)))

Web of Science:

TS=(mhealth OR m-health OR iOS OR "mobile health" OR "activity tracker" OR wearable OR tablet OR "personal digital assistant" OR PDA OR "short message service" OR SMS OR "text message" OR android OR iphone OR cellphone OR "mobile telephone" OR "cell phone" OR smartphone OR smart-phone OR "mobile device" OR "mobile phone" OR “mobile app” OR “mobile application” OR “cellular phone” OR “cellular telephone”) AND TS=(("physical activity" OR fitness OR exercise OR sport OR "active living" OR "leisure activity" OR “motor activity”) OR ("physical inactivity" OR "sedentary behavior" OR "sedentary behaviour" OR sitting OR “sedentary lifestyle”)) AND TS=(intervention OR trial OR program) AND TS= (("clinical trial" OR "controlled trial" OR "controlled study" OR RCT OR "double blind") OR ("pragmatic trial" OR "practical trial" OR PCT OR "ecological trial" OR "dynamic trial" OR "real-world" OR "real world"))
