# Supplementary material for: Assessing the Pragmatic Nature of Mobile Health Interventions Promoting Physical Activity: Systematic Review and Meta-analysis
Source: JMIR Mhealth Uhealth. 2023 May 4;11:e43162. doi: 10.2196/43162 (PMC10196895; doi:10.2196/43162)
Supplement: Multimedia Appendix 3 [file mhealth_v11i1e43162_app3.pdf]

Article ID
Study title
Author
**Year**
**Country**
**Continent**
**Journal**
primary purpose
study design
Period of data collection
Target population (e.g., inactive
Age (i.e., mean, range)
Gender of population (i.e., n, %)
Sample size (i.e., overall,
Description of intervention (i.e.,
Was the app the sole intervention or
Did the intervention incorporate
Did the program target any other
Description of comparator (i.e.,
Description of app features (e.g., step
Physical activity outcome measured
Objective physical activity measuring
Outcome measure time points (e.g.,
Secondary outcome measures (e.g.,
App engagement (i.e., adherence
Results intervention group (i.e.,
Results comparator group (i.e.,
Satistical analyses performed (e.g.,
Lost to Follow Up
Distributional Assumptions
