# Supplementary material for: Assessing the Pragmatic Nature of Mobile Health Interventions Promoting Physical Activity: Systematic Review and Meta-analysis
Source: JMIR Mhealth Uhealth. 2023 May 4;11:e43162. doi: 10.2196/43162 (PMC10196895; doi:10.2196/43162)
Supplement: Multimedia Appendix 4 [file mhealth_v11i1e43162_app4.pdf]

**ABOUT THE ARTICLE/STUDY**	**Article ID**	Located in the assignment sheet
	**Source**	If original research does not report all needed information or provides inaccurate/insufficient information, potential companion articles may be used in a separate row to complete extraction. (original research = 1; companion article = 2)
	**Author, Year**	Self explanatory
	**Country**	Self explanatory
	**Study Design**	Study Design<br>RCT, CC, Observational, etc.
	**Objective**	What is the purpose of the manuscript; provide details that will give us highlights of the paper (e.g., the purpose of this paper was to report on the short term effectiveness of a dietary program, with special attention on the feasibility (adoption) of the program in a clinical setting).
	**Companion Article (Y/N)**	Is there a companion article to this particular intervention? (Y=1; N=0)
	**Type**	Provide further information on companion article (e.g. study protocol, CONSORT checklist, etc) (N/A=999)
	**Reviewer**	Self explanatory
**REACH VARIABLES**	**Recruitment protocol (Y/N/I)**	(Y=1; N=0; inaccurate/insufficient=2)
	**Recruitment protocol (list)**	Recruitment protocol (list)
	**Exclusion criteria (Y/N/I)**	(Y=1; N=0; inaccurate/insufficient=2)
	**Exclusion criteria (list)**	Explicit statement of characteristics that would prevent a potential participant from being eligible to participate.
	**Inclusion criteria (Y/N/I)**	(Y=1; N=0; inaccurate/insufficient=2)
	**Inclusion criteria (rationale)**	Explicit statement of characteristics of the target population that were used to determine if a potential participant is eligible to participate.
	**Denominator (Y/N/I)**	(Y=1; N=0; inaccurate/insufficient=2)
	**Denominator (rationale)**	different levels of denominators:<br><br>Level 1: total number of target audience exposed to recruitment<br>Level 2: total number of individuals assessed for eligibility and found eligible.<br><br>inaccurate = only reported number of individuals assessed for eligibility but not reported how many found eligible / declined participation
	**Denominator number (n)**	list denominator number or if not stated, the total number assessed for eligibility (N/A=999)
	**Participation rate (authors) (Y/N/I)**	(Y=1; N=0; inaccurate=2)
	**Participation rate (authors) (rationale)**	report if participation rate is explicitly stated by authors (N/A=999)
	**Participation rate (authors) (%)**	provide participation rate (%) explicitly stated by authors (N/A=999)
	**Participation rate (reviewers) (rationale)**	provide rationale on participation rate calculated by reviewer (if authors did not state or inaccurately reported) (N/A=999)<br><br>different levels of study reach<br>Level 1 (broadest): proportion of sample size / number of people exposed to recruitment<br><br>Level 2: proportion of sample size / individuals found eligible (found eligible = assessed for eligibility - not eligible/unclear eligibilty)

	**Participation rate (reviewers) (%)**	list calculated participation rate (%) (N/A=999)
	**Demo comparison non-participants (Y/N/I)**	(Y=1; N=0; inaccurate/insufficient=2)
	**Demo comparison non-participants (list)**	Total number of comparisons made between target population and study sample with a description of what those comparisons were<br>Example: Participants were compared to non-participants for: activity level, gender, age... (N/A=999)<br><br>different levels of representativeness:<br><br>Indicator 1: comparsion between sample size and eligible idividuals that declined participation<br><br>Indicator 2: comparsion between sample size and target audience<br><br>Indicator 3: comparsion between eligible (but declined participation) and target audience
	**Demo comparison non-participants (sig diff) (list)**	Total number of statistically significant comparisons made between target population and study sample with a description of what those comparisons were. (N/A=999)
	**Use qualitative methods to understand reach/recruitment (Y/N/I)**	(Y=1; N=0; inaccurate/insufficient=2)
	**Use qualitative methods to understand reach/recruitment (rationale)**	Reporting on non-quantitative aspects of reach. Observations in words, sentences, descriptions or codes. Some common methods include key informant interviews, focus groups, or even field notes that provide information on perceptions, feelings, opinions, experiences, etc. (N/A=999)
	**Measure of primary outcome**	report author stated measure of primary outcome
	**Primary outcome results**	report primary outcome results as stated in original 'results' section of data.
	**Primary outcome comparison to public health goal (Y/N/I)**	(Y=1; N=0; inaccurate/insufficient=2)<br><br>1=comparsion made to objectives listed in public health guidelines
**EFFECTIVENESS VARIABLES**	**Primary outcome comparison to public health goal (rationale)**	provide information (N/A=999)
	**QoL (Y/N/I)**	(Y=1; N=0; inaccurate/insufficient=2)
	**QoL (rationale)**	Includes a measure of quality of life with some latitude for coding articles that refer to well-being or satisfaction with life. The QOL variable includes validated QOL scales as well, it is expanded to include other variables that are self-reported health or quality (i.e., quality of sleep, flourishing) (N/A=999)
	**Negative outcome (Y/N/I)**	(Y=1; N=0; inaccurate/insufficient=2)
	**Negative outcome (rationale)**	To evaluate unanticipated consequences and results that may be a product of the intervention and may have caused unintended harm.<br>Example: In a PA promotion program, female participants had an increased rate of injury. (N/A=999)
	**Attrition (reported/reviewers can calculate) (Y/N/I)**	(Y=1; N=0; inaccurate/insufficient=2)
	**Attrition (n)**	list (n) (N/A=999)
	**Attrition (%)**	list (%) (N/A=999)

	**Attrition (rationale)**	The proportion that was lost to follow-up or dropped out of the intervention. This is calculated by dividing the number of participants who did not complete the intervention by the number of participants who began the intervention. (N/A=999)
	**Any Short-Term Attrition info (Y/N/I)**	(Y=1; N=0; inaccurate/insufficient=2)
	**Any Short-Term Attrition info (rationale)**	list provided information (e.g. dropout reasons; differences in participant characteristics; etc) (N/A=999)
	**Use qualitative methods to understand outcomes (Y/N/I)**	(Y=1; N=0; inaccurate/insufficient=2)
	**Use qualitative methods to understand outcomes (rationale)**	Obtaining qualitative feedback from participants on the degree to which they felt the intervention was efficacious/effective. Some common methods include focus groups, interviews, diaries (text/pictures). (N/A=999)
	**Efficacy vs. effectiveness study (Author stated) (Y/N/I)**	(Y=1; N=0; inaccurate/insufficient=2)
	**Efficacy vs. effectiveness study (Author stated) (list)**	explicitly author stated information (N/A=999)
	**Efficacy vs. effectiveness study (Reviewer opinion if not stated by authors)**	Reviewer opinion if author did not state or may be incorrect (N/A=999)
	**Type of follow-up (authors) (Y/N/I)**	(Y=1; N=0; inaccurate/insufficient=2)
	**Type of follow-up (authors)**	Intent to treat analysis: when participants in trials are analyzed in the groups to which they were randomized, regardless of whether they received or adhered to the allocated intervention. Example, will typically use the term intent to treat or will describe an imputation that was used to account for missing data in the analysis.<br><br>Present at Follow-up/modified ITT analysis: when only participants who completed the follow-up assessment are included in the analysis of efficacy/effectiveness.<br>Example: Only those participants who completed both the baseline and follow-up measures were included in the analysis.<br><br>(N/A=999)
	**Type of follow-up (Reviewer)**	provide rationale if type of follow up was not stated or information may be incorrect (N/A=999)
	**Imputation (Y/N/I)**	(Y=1; N=0; inaccurate/insufficient=2)
	**Imputation (rationale)**	Substitution of some value for missing data.<br>Example: Multiple imputation methods were used to impute missing minutes of PA data at 3 months… (N/A=999)
	**Mediation analysis (Y/N/I)**	(Y=1; N=0; inaccurate/insufficient/intended but didn't do/ didn't reported=2)
	**Mediation variables (list)**	Variables that explain the extent to which the particular variable accounts for the relationship between the predictor and the criterion.<br><br>insufficient= no true mediation analysis performed (N/A=999)
	**Moderation analysis (Y/N/I)**	(Y=1; N=0; inaccurate/insufficient/intended but didn't do/ didn't report=2)
	**Moderation variables (list)**	List of variables: qualitative (e.g., sex, race, class) or quantitative (e.g., level of reward) that influence the direction and/or strength of the relationship between the treatment and the outcome.<br><br>insufficient= no true moderation analysis performed (N/A=999)

	**Robustness across subgroups (Y/N/I)**	(Y=1; N=0; inaccurate/insufficient/intended but didn't do/ didn't report=2)
	**Robustness across subgroups (list)**	list of subgroups being analysed<br><br>(N/A=999)
**ADOPTION VARIABLES - SETTING**	**Study setting IX**	The explicit statement of characteristics of the location of the intervention.<br><br>(recruitment; run-in; delivery; assessment)
	**Study setting Control**	The explicit statement of characteristics of the location of the intervention.<br><br>(recruitment; run-in; delivery; assessment)
	**Setting exclusions (Y/N)**	(Y=1; N=0; inaccurate/insufficient=2)
	**Setting exclusions (list)**	The explicit statement of characteristics of the setting that were used to determine if a potential setting is eligible to participate. (N/A=999)
	**Setting comparison (Y/N)**	(Y=1; N=0; inaccurate/insufficient=2)
	**Setting comparison (rationale)**	Total number and type of comparisons of targeted intervention sites and those that participated, including a list: size, location, etc. (N/A=999)
	**Setting sample size (n)**	list setting sample size (n) (N/A=999)
	**Setting demonimator (Y/N)**	(Y=1; N=0; inaccurate/insufficient=2)
	**Setting demonimator (n)**	provide denominator (n) (N/A=999)
	**Setting adoption rate (%)**	provide rate (%) (N/A=999)
	**Setting participation rate (%)**	provide rate (%) (N/A=999)
	**Use qualitative methods to understand setting adoption (Y/N)**	(Y=1; N=0; inaccurate/insufficient=2)
	**Use qualitative methods to understand setting adoption (rationale)**	Used qualitative methods to understand the process of adoption. (N/A=999)
	**Adoption cost (Y/N)**	(Y=1; N=0; inaccurate/insufficient=2)
	**Adoption cost (rationale)**	list one time (start up) cost of adoption across all levels of the intervention<br>(e.g. recruitment; software (app development); hardware (intervention & assessment devices); intervention/assessment location; participant compensation etc.)<br><br>(N/A=999)
**ADOPTION VARIABLES - STAFF**	**Staff Description (i.e. intervention delivery agent) (Y/N)**	(Y=1; N=0; inaccurate/insufficient=2)
	**Staff description (List)**	Training or educational background in relevant area; Degrees, certifications of delivery agents (such as PhD, Masters, Registered Dietitian, etc.) (N/A=999)
	**Other Personnel info**	characteristic of delivery agent (e.g. gender; age) (N/A=999)
	**Staff exclusions (Y/N)**	(Y=1; N=0; inaccurate/insufficient=2)
	**Staff exclusions (list)**	The explicit statement of characteristics of the delivery agents that were used to determine if a potential delivery agent is eligible to participate. (N/A=999)
	**Staff comparison (Y/N)**	(Y=1; N=0; inaccurate/insufficient=2)
	**Staff comparison (rationale)**	Total number and type of comparisons of targeted staff members and those that participated, including a list: age, BMI, education (N/A=999)
	**Staff sample size (n)**	The total staff members that agreed to participate. (N/A=999)
	**Staff demonimator (Y/N)**	(Y=1; N=0; inaccurate/insufficient=2)
	**Staff demonimator (n)**	list denominator (n) (N/A=999)

	**Staff adoption rate (Y/N)**	(Y=1; N=0; inaccurate/insufficient=2)
	**Staff adoption rate (%)**	list adoption rate (N/A=999)
	**Staff participation rate (Y/N)**	(Y=1; N=0; inaccurate/insufficient=2)
	**Staff participation rate (%)**	The proportion of the staff that was contacted and participated. (N/A=999)
	**Use qualitative methods to understand staff adoption (Y/N)**	(Y=1; N=0; inaccurate/insufficient=2)
	**Use qualitative methods to understand staff adoption (rationale)**	Used qualitative methods to understand the process of adoption. (N/A=999)
	**Delivered as intended (Y/N)**	(Y=1; N=0; inaccurate/insufficient=2)
**IMPLEMENTATION VARIABLES**	**Delivered as intended (rationale)**	Description of fidelity to the intervention protocol. Example: a checklist of program components assessed by delivery agent(s) (N/A=999)
	**Adaptations (Y/N)**	(Y=1; N=0; inaccurate/insufficient=2)
	**Adaptations (list)**	list information (N/A=999)
	**Cost (time) (Y/N)**	(Y=1; N=0; inaccurate/insufficient=2)
	**Cost (time) (rationale)**	list time cost across all levels of the intervention (N/A=999)
	**Cost (money) (Y/N)**	(Y=1; N=0; inaccurate/insufficient=2)
	**Cost (money) (rationale)**	The ongoing cost of delivery across all levels of the intervention (N/A=999)
	**Consistency across groups (Y/N)**	(Y=1; N=0; inaccurate/insufficient=2)
	**Consistency across groups (rationale)**	Description of the degree of similarities between multiple settings sites & delivery agents (N/A=999)
	**Participant received IX componets (Y/N)**	(Y=1; N=0; inaccurate/insufficient=2)
	**Participant received IX componets (rationale)**	describes the intervention and its strategies/components
	**Use qualitative methods to understand implementation (Y/N)**	(Y=1; N=0; inaccurate/insufficient=2)
	**Use qualitative methods to understand implementation (rationale)**	Used qualitative methods to understand the process of implementation. (N/A=999)
	**Maintenance? (Y/N)**	(Y=1; N=0; inaccurate/insufficient=2)
**MAINTENANCE (INDIVIDUAL) VARIABLES**	**Maintenance measure of primary outcome**	report author stated measure of primary outcome (N/A=999)
	**Maintenance Primary outcome type of data**	quantitative and/or qualitative (N/A=999)
	**Maintenance Primary outcome results**	Maintenance Primary outcome results
	**Maintenance measure secondary outcome**	report author stated measure of secondaty outcome (N/A=999)
	**Maintenance Secondary outcome type of data**	quantitative and/or qualitative (N/A=999)
	**Maintenance Secondary outcome results**	report secondary outcome results as stated in original 'results' section of data. (N/A=999)
	**Maintenance of primary outcome comparison to public health goal (Y/N)**	(Y=1; N=0; inaccurate/insufficient=2)

	**Maintenance of primary outcome comparison to public health goal (rationale)**	provide information (N/A=999)
	**Maintenance QoL (Y/N)**	(Y=1; N=0; inaccurate/insufficient=2)
	**Maintenance QoL (rationale)**	Includes a measure of quality of life with some latitude for coding articles that refer to well-being or satisfaction with life. (N/A=999)
	**Maintenance Negative outcome (Y/N)**	(Y=1; N=0; inaccurate/insufficient=2)
	**Maintenance Negative outcome (rationale)**	To evaluate unanticipated consequences and results that may be a product of the intervention and may have caused unintended harm.<br>Example: In a PA promotion program, female participants had an increased rate of injury.<br><br>(N/A=999)
	**Maintenance robustness across subgroups (Y/N/I)**	(Y=1; N=0; inaccurate/insufficient/intended but didn't do/ didn't report=2)
	**Maintenance robustness across subgroups (list)**	list of subgroups being analysed<br><br>(N/A=999)
	**Long term Percent attrition (reported/reviewers can calculate) (Y/N)**	(Y=1; N=0; inaccurate/insufficient=2)
	**Long term Percent attrition (n)**	list (n) (N/A=999)
	**Long term Percent attrition (%)**	Describe the degree to which participants were lost to follow-up (and the reasons) during the period in time from the interventions completion to the follow-up (%) (N/A=999)
	**Use qualitative methods to understand maintenance (Y/N)**	(Y=1; N=0; inaccurate/insufficient=2)
	**Use qualitative methods to understand maintenance (rationale)**	Used qualitative methods to understand the process of individual level maintenance of changes to the primary outcome.
	**Program ongoing (Y/N)**	(Y=1; N=0; inaccurate/insufficient=2)
**MAINTENANCE (SETTING) VARIABLES**	**Program ongoing (rationale)**	Description of program continuation after completion of the research study.<br>(N/A=999)
	**Long-term adaptations (Y/N)**	(Y=1; N=0; inaccurate/insufficient=2)
	**Long-term adaptations (rationale)**	Description of any changes that were made to the original program (N/A=999)
	**Sustainable system (Y/N)**	(Y=1; N=0; inaccurate/insufficient=2)
	**Sustainable system (rationale)**	Description of the how the intervention was integrated into the delivery system through methods such as policy changes, job description changes.
	**Use qualitative methods to undertand setting-level institutionalization (Y/N)**	(Y=1; N=0; inaccurate/insufficient=2)
	**Use qualitative methods to undertand setting-level institutionalization (rationale)**	Used qualitative methods to understand the process of intervention sustainability at the organizational level
**PRECIS-2 VARIABLES (Code each as 1-5)**	**Eligibility**	1-5
	**Recruitment**	1-5
	**Setting**	1-5
	**Organization**	1-5
	**Flexibility: delivery**	1-5
	**Flexibility: adherence**	1-5
	**Follow-up**	1-5
	**Primary outcome**	1-5
	**Primary analysis Unsure-imputation/missing data not addressed**	1-5
	**Score**	9-45

**PRECIS EXPLANATIONS**	**Eligibility**	provide rationale:<br><br>to what extent are the participants in the trial similar to those who would receive this intervention if it was part of usual care? For example, score 5 for very pragmatic criteria essentially identical to those in usual care; score 1 for a very explanatory approach with lots of exclusions (e.g. those who don't comply, respond to treatment, or are not at high risk for primary outcome, are children or elderly), or uses many selection tests not used in usual care.
	**Recruitment**	provide rationale:<br><br>how much extra effort is made to recruit participants over and above what that would be used in the usual care setting to engage with patients? For example, score 5 for very pragmatic recruitment through usual appointments or clinic; score 1 for a very explanatory approach with targeted invitation letters, advertising in newspapers, radio plus incentives and other routes that would not be used in usual care.
	**Setting**	provide rationale:<br><br>how different is the setting of the trial and the usual care setting? For example, score 5 for a very pragmatic choice using identical settings to usual care; score 1, for a very explanatory approach with only a single centre, or only specialised trial or academic centres.
	**Organization**	provide rationale:<br><br>how different are the resources, provider expertise and the organisation of care delivery in the intervention arm of the trial and those available in usual care? For example, score 5 for a very pragmatic choice that uses identical organisation to usual care; score 1 for a very explanatory approach if the trial increases staff levels, gives additional training, require more than usual experience or certification and increase resources.
	**Flexibility: delivery**	provide rationale:<br><br>how different is the flexibility in how the intervention is delivered and the flexibility likely in usual care? For example, score 5 for a very pragmatic choice with identical flexibility to usual care; score 1 for a very explanatory approach if there is a strict protocol, monitoring and measures to improve compliance, with specific advice on allowed co-interventions and complications.
	**Flexibility: adherence**	provide rationale:<br><br>how different is the flexibility in how participants must adhere to the intervention and the flexibility likely in usual care? For example, score 5 for a very pragmatic choice involving no more than usual encouragement to adhere to the intervention; score 1 for a very explanatory approach that involves exclusion based on adherence, and measures to improve adherence if found wanting. In some trials eg surgical trials where patients are being operated on or Intensive Care Unit trials where patients are being given IV drug therapy, this domain is not applicable as there is no compliance issue after consent has been given, so this score should be left

	**Follow-up**	provide rationale:<br><br>how different is the intensity of measurement and follow-up of participants in the trial and the likely follow-up in usual care? For example, score 5 for a very pragmatic approach with no more than usual follow up; score 1 for a very explanatory approach with more frequent, longer visits, unscheduled visits triggered by primary outcome event or intervening event, and more extensive data collection
	**Primary outcome**	provide rationale:<br><br>to what extent is the trial's primary outcome relevant to participants? For example, score 5 for a very pragmatic choice where the outcome is of obvious importance to participants; score 1 for a very explanatory approach using a surrogate, physiological outcome, central adjudication or use assessment expertise that is not available in usual care, or the outcome is measured at an earlier time than in usual care.
	**Primary analysis**	provide rationale:<br><br>to what extent are all data included in the analysis of the primary outcome? For example, score 5 for a very pragmatic approach using intention to treat with all available data; score 1 for a very explanatory analysis that excludes ineligible post-randomisation participants, includes only completers or those following the treatment protocol
