# Supplementary material for: Assessing the Pragmatic Nature of Mobile Health Interventions Promoting Physical Activity: Systematic Review and Meta-analysis
Source: JMIR Mhealth Uhealth. 2023 May 4;11:e43162. doi: 10.2196/43162 (PMC10196895; doi:10.2196/43162)
Supplement: Multimedia Appendix 5 [file mhealth_v11i1e43162_app5.pdf]

1st Autor (Year)	Domain	Signalling Question	Elaboration	Response
Direito (2015)	Risk of bias arising from the randomization process	Was the allocation sequence random?	"Stratified block randomization in variable blocks was used to maintain balance across gender, an important prognostic factor. A biostatistician prepared the randomization scheme in advance by using a computer-generated randomization table."	Yes
		Was the allocation sequence concealed until participants were enrolled and assigned to interventions?	"Based on the randomization scheme, a research assistant prepared opaque-sealed envelopes containing group referral so that the researchercould not identify group assignment. The envelope was opened by each participant after completion of baseline assessment. Given the nature of the intervention, it was not possible to blind participants. However, allocation concealment was maintained up to the point of randomization."	Yes
		Did baseline differences between intervention groups suggest a problem with the randomization process?	No useful baseline information available (study reported only baseline characteristics of participants in the final analysis).	No information
	Risk-of- bias judgement			**Low risk of bias**
	Risk of bias due to deviations from the intended interventions	Were participants aware of their assigned intervention during the trial?	"Given the nature of the intervention, it was not possible to blind participants."	Yes
		Were carers and people delivering the interventions aware of	Masking: Open (masking not used)	Yes

		participants' assigned intervention during the trial?		
		Were there deviations from the intended intervention that arose because of the trial context?	"There were no deviations to methods after trial commencement."	No
		Were these deviations likely to have affected the outcome?	NA	NA
		Were these deviations from intended intervention balanced between groups?	NA	NA
		Was an appropriate analysis used to estimate the effect of assignment to intervention?	"Intention-to-treat analysis was performed" (51/51 analyzed)	Yes
		Was there potential for a substantial impact (on the result) of the failure to analyse participants in the group to which they were randomized?	NA	NA
	Risk-of- bias judgement			**Low risk of bias**
	Risk of bias due to missing outcome data	Were data for this outcome available for all, or nearly all, participants randomized?	"Follow-up assessments were completed for 96% of participants. For accelerometry, 48 of 51 (94%) participants provided valid data for analysis at baseline, whereas compliance with wearing the device slightly decreased at post intervention (46/51, 90%)"	Yes

		Is there evidence that the result was not biased by missing outcome data?	NA	NA
		Could missingness in the outcome depend on its true value?	NA	NA
		Is it likely that missingness in the outcome depended on its true value?	NA	NA
	Risk-of- bias judgement			**Low risk of bias**
	Risk of bias in measurement of the outcome	Was the method of measuring the outcome inappropriate?	"Objectively measured physical activity is assessed using the GT1M Actigraph, a motion sensor shown to be a valid and reliable instrument for assessing physical activity in young people."	No
		Could measurement or ascertainment of the outcome have differed between intervention groups?	Same method of measurement in all conditions	No
		Were outcome assessors aware of the intervention received by study participants?	"The outcome assessor will not be blinded to the treatment allocation…"	Yes
		Could assessment of the outcome have been influenced by knowledge of intervention received?	Assessment of outcome utilize objective measures, involving little to no judgement	No

		Is it likely that assessment of the outcome was influenced by knowledge of intervention received?	NA	NA
	Risk-of- bias judgement			**Low risk of bias**
	Risk of bias in selection of the reported result	Were the data that produced this result analysed in accordance with a pre-specified analysis plan that was finalized before unblinded outcome data were available for analysis?	Study protocol available.<br><br>"There were no deviations to methods after trial commencement."	Yes
		Is the numerical result being assessed likely to have been selected, on the basis of the results, from multiple eligible outcome measurements (e.g. scales, definitions, time points) within the outcome domain?	Mean (SD) for MVPA presented at all time-points of measurement (Pre/Post). Adjusted difference (95% CI) presented.	No
		Is the numerical result being assessed likely to have been selected, on the basis of the results, from multiple eligible analyses of the data?	only adjusted mean differences presented (does not raise concerns as unadjusted differences were not mentioned in protocol)	PN
	Risk-of- bias judgement			**Low risk of bias**
	**Overall risk of bias score**			**Low risk of bias**

Edney (2020)	Risk of bias arising from the randomization process	Was the allocation sequence random?	"using permuted blocks with block sizes of 9, 12 or 15, to ensure a balance of clusters across experimental conditions throughout the trial."	Yes
		Was the allocation sequence concealed until participants were enrolled and assigned to interventions?	"An independent allocation officer will determine block size and allocation schedule using a random number generator and a computer generated randomisation schedule. Block size and block allocation sequence will be concealed from the person enrolling the participants."<br><br>"Researchers will not have direct access to the allocation schedule."	Yes
		Did baseline differences between intervention groups suggest a problem with the randomization process?	"There were no statistically significant differences in demographic characteristics between the 3 groups at baseline, or between study completers and non completers."	No
	Risk-of- bias judgement			**Low risk of bias**
	Risk of bias due to deviations from the intended interventions	Were participants aware of their assigned intervention during the trial?	"masking: single (participant)"	PN
		Were carers and people delivering the interventions aware of	"masking: single (participant)"	PY

		participants' assigned intervention during the trial?		
		Were there deviations from the intended intervention that arose because of the trial context?	"There were no deviations from the prespecified protocol during the trial"	No
		Were these deviations likely to have affected the outcome?	NA	NA
		Were these deviations from intended intervention balanced between groups?	NA	NA
		Was an appropriate analysis used to estimate the effect of assignment to intervention?	"Intention-to-treat analysis and random effects mixed modelling will be used to assess whether there are significant differences in changes to the primary outcome measure." (444/444 analysed)	Yes
		Was there potential for a substantial impact (on the result) of the failure to analyse participants in the group to which they were randomized?	NA	NA
	Risk-of- bias judgement			**Low risk of bias**
	Risk of bias due to missing outcome data	Were data for this outcome available for all, or nearly all, participants randomized?	"Retention was high, with 402 (91%) and 383 (86%) participants providing outcome assessments at 3- and 9-month follow up, respectively."	PN

		Is there evidence that the result was not biased by missing outcome data?	“The most appropriate procedure for handling missing data will be selected after inspecting the amount and pattern of missingness.” Maximum likelihood estimation was used.	Yes
		Could missingness in the outcome depend on its true value?	NA	NA
		Is it likely that missingness in the outcome depended on its true value?	NA	NA
	Risk-of- bias judgement			**Low risk of bias**
	Risk of bias in measurement of the outcome	Was the method of measuring the outcome inappropriate?	GENEActiv accelerometers: “excellent overall intra- and inter-instrument reliability for activity count against a mechanical shaker. r=0.89 (95% CI, 0.84-0.94) when compared to indirect calorimetry”	No
		Could measurement or ascertainment of the outcome have differed between intervention groups?	Same method of measurement in all conditions	No
		Were outcome assessors aware of the intervention received by study participants?	“Given the nature of the study, study personnel were aware of the allocated group once each team was formally enrolled.”	Yes

			“masking: single (participant)”	
		Could assessment of the outcome have been influenced by knowledge of intervention received?	Assessment of outcome utilize objective measures, involving little to no judgement	No
		Is it likely that assessment of the outcome was influenced by knowledge of intervention received?	NA	NA
	Risk-of- bias judgement			**Low risk of bias**
	Risk of bias in selection of the reported result	Were the data that produced this result analysed in accordance with a pre-specified analysis plan that was finalized before unblinded outcome data were available for analysis?	Study protocol available.<br><br>“There were no deviations from the prespecified protocol during the trial.”	Yes
		Is the numerical result being assessed likely to have been selected, on the basis of the results, from multiple eligible outcome measurements (e.g. scales, definitions, time points) within the outcome domain?	Mean (SD) + Effect size for MVPA presented at all time-points of measurement (Pre/Post/Follow-up)	No
		Is the numerical result being assessed likely to have been selected, on the basis of the results, from multiple eligible analyses of the data?	only adjusted models presented (but stated that no difference to unadjusted models)	No

	Risk-of- bias judgement			**Low risk of bias**
	**Overall risk of bias score**			**Low risk of bias**
Fanning (2017)	Risk of bias arising from the randomization process	Was the allocation sequence random?	"Following the call, they were also assigned by study staff to one of four intervention conditions based upon the order in which they were screened such that two individuals were assigned to each group in a rolling fashion."<br><br>"Additionally, participants were assigned to treatment conditions in pairs and in a rolling fashion based on order of telephone call. This opens the possibility of bias, and future iterations of this research would benefit from a true randomization procedure."	No
		Was the allocation sequence concealed until participants were enrolled and assigned to interventions?	"Randomization status was concealed from participants throughout the study period, and they were debriefed on each the four versions of the application and remaining app modules after completing all study procedures."	Yes
		Did baseline differences between intervention groups suggest a problem with the randomization process?	"Initial linear regression analyses revealed education level was significantly related to activity at baseline and was entered as a covariate into the factorial RM-ANOVA. Additionally, the RM-	Yes

			ANOVA for MVPA revealed a significant effect for those with access to points, and so baseline MVPA was included as a covariate in all analyses”	
	Risk-of- bias judgement			**Some concerns**
	Risk of bias due to deviations from the intended interventions	Were participants aware of their assigned intervention during the trial?	“masking: single (participant)“	PN
		Were carers and people delivering the interventions aware of participants' assigned intervention during the trial?	“masking: single (participant)“	PY
		Were there deviations from the intended intervention that arose because of the trial context?	NI	NI
		Were these deviations likely to have affected the outcome?	NA	NA
		Were these deviations from intended intervention balanced between groups?	NA	NA
		Was an appropriate analysis used to estimate the effect of assignment to intervention?	mITT<br><br>"96 individuals returned activity monitors with sufficient data for inclusion in analyses"<br>(96/116 analysed)	Yes

		Was there potential for a substantial impact (on the result) of the failure to analyse participants in the group to which they were randomized?	NA	NA
	Risk-of- bias judgement			**Some concerns**
	Risk of bias due to missing outcome data	Were data for this outcome available for all, or nearly all, participants randomized?	"Of the 116 eligible participants who completed baseline requirements (i.e., 88% of the initial recruitment goal), 103 completed follow-up questionnaires, 96 individuals returned activity monitors with sufficient data for inclusion in analyses, and 97 completed feedback forms" (96/116=82.76%)	No
		Is there evidence that the result was not biased by missing outcome data?	"First, missing values were imputed using the multiple imputation feature in SPSS version 22 (IBM Corp., Armonk, NY), and these data were checked for extreme outliers and Windsorized to three standard deviations when necessary."	PN
		Could missingness in the outcome depend on its true value?	Reported reasons for lost to follow-up indicate that missing data might depend on its true value (e.g. uninterested in continuing / not able to contact)	PY

		Is it likely that missingness in the outcome depended on its true value?	Differences between participant groups in the proportions of missing outcome data (based on drop outs only) is similar	PN
	Risk-of- bias judgement			**Some concerns**
	Risk of bias in measurement of the outcome	Was the method of measuring the outcome inappropriate?	"Actigraph accelerometers (Modelo GT1M or newer)"	No
		Could measurement or ascertainment of the outcome have differed between intervention groups?	Same method of measurement in all conditions	No
		Were outcome assessors aware of the intervention received by study participants?	"masking: single (participant)"	Yes
		Could assessment of the outcome have been influenced by knowledge of intervention received?	Assessment of outcome utilize objective measures, involving little to no judgement	No
		Is it likely that assessment of the outcome was influenced by knowledge of intervention received?	NA	NA
	Risk-of- bias judgement			**Low risk of bias**

	Risk of bias in selection of the reported result	Were the data that produced this result analysed in accordance with a pre-specified analysis plan that was finalized before unblinded outcome data were available for analysis?	no study protocol available.	NI
		Is the numerical result being assessed likely to have been selected, on the basis of the results, from multiple eligible outcome measurements (e.g. scales, definitions, time points) within the outcome domain?	Mean (SD) for MVPA presented at all time-points of measurement (Pre/Post) / MVPA Effect sizes (d (95% CI)) baseline to follow up presented	No
		Is the numerical result being assessed likely to have been selected, on the basis of the results, from multiple eligible analyses of the data?	All eligible reported results for the outcome measurement correspond to all intended analyses.	No
	Risk-of- bias judgement			**Some concerns**
	**Overall risk of bias score**			**High risk of bias**
Fukuoka (2019)	Risk of bias arising from the randomization process	Was the allocation sequence random?	"Permuted-blocked randomization will be used to ensure that the number of participants in the three treatment groups is close to our goal of an exact 1:1:1 ratio. Block size will vary randomly from three to nine in a schedule that is not known to investigators and research staff."	Yes

		Was the allocation sequence concealed until participants were enrolled and assigned to interventions?	"The data management staff will prepare a set of sealed, opaque envelopes, numbered consecutively, and containing the study group assignment."	Yes
		Did baseline differences between intervention groups suggest a problem with the randomization process?	"No significant differences in baseline characteristics were observed among the 3 groups"	No
	Risk-of- bias judgement			**Low risk of bias**
	Risk of bias due to deviations from the intended interventions	Were participants aware of their assigned intervention during the trial?	"Due to the nature of the mobile phone intervention, blinding the participant, research staff, and investigator is not possible. In order to minimize expectation bias, participants are informed only that we are testing two different physical activity interventions (pedometer intervention versus pedometer plus mobile phone application intervention). Participants who are randomized into the PLUS and REGULAR groups are blinded to their maintenance intervention (PLUS or REGULAR) assignment until they complete the 3-month mobile phone and pedometer based intervention."	Yes

		Were carers and people delivering the interventions aware of participants' assigned intervention during the trial?	"The mPED study was an unblinded, parallel randomized clinical trial conducted with 3 groups."	Yes
		Were there deviations from the intended intervention that arose because of the trial context?	All participants received assigned intervention. Procedures did not deviate from study protocol.<br><br>"No differences in hospital admissions, emergency department or urgent care facility visits, or other adverse events were observed between the control, and regular, and plus groups during the 3-month intervention period and among the 3 groups during the 6-month maintenance period"	No
		Were these deviations likely to have affected the outcome?	NA	NA
		Were these deviations from intended intervention balanced between groups?	NA	NA
		Was an appropriate analysis used to estimate the effect of assignment to intervention?	"Data were analyzed using intention to treat" (210/210 analysed)	Yes
		Was there potential for a substantial impact (on the result) of the failure to analyse participants in the group to which they were randomized?	NA	NA
	Risk-of- bias judgement			**Low risk of bias**

	Risk of bias due to missing outcome data	Were data for this outcome available for all, or nearly all, participants randomized?	"Physical activity outcomes measured using the accelerometer, including daily step counts and MVPA, were missing for approximately 12% of follow-up days during the 9-month trial period." (Data only needed for 3-month trial period, likely that dropout was not greater than 10% during this period)	PY
		Is there evidence that the result was not biased by missing outcome data?	"If dropout is substantial, sensitivity analyses will be conducted in which we multiply impute missing outcome data under the conservative assumption that exercise levels fall back to their baseline levels after study dropout"	Yes
		Could missingness in the outcome depend on its true value?	NA	NA
		Is it likely that missingness in the outcome depended on its true value?	NA	NA
	Risk-of- bias judgement			**Low risk of bias**
	Risk of bias in measurement of the outcome	Was the method of measuring the outcome inappropriate?	Omron Active Style Pro HJA-350IT. "This accelerometer has	No

			been validated before, and a detailed description was published previously”	
		Could measurement or ascertainment of the outcome have differed between intervention groups?	Same method of measurement in all conditions	No
		Were outcome assessors aware of the intervention received by study participants?	“Due to the nature of the mobile phone intervention, blinding the participant, research staff, and investigator is not possible.”	Yes
		Could assessment of the outcome have been influenced by knowledge of intervention received?	Assessment of outcome utilize objective measures, involving little to no judgement	No
		Is it likely that assessment of the outcome was influenced by knowledge of intervention received?	NA	NA
	Risk-of- bias judgement			**Low risk of bias**
	Risk of bias in selection of the reported result	Were the data that produced this result analysed in accordance with a pre-specified analysis plan that was finalized before unblinded outcome data were available for analysis?	Study protocol available.	Yes
		Is the numerical result being assessed likely to have been	Mean (SD) for MVPA presented only at baseline (daily points of	No

		selected, on the basis of the results, from multiple eligible outcome measurements (e.g. scales, definitions, time points) within the outcome domain?	measure for 9-months visualized in a graph though) / mean difference presented	
		Is the numerical result being assessed likely to have been selected, on the basis of the results, from multiple eligible analyses of the data?	"Results for total daily steps were similar in a per-protocol analysis restricted to observations with valid accelerometer data." (ITT presented and mentioned that per-protocol analysis were similar)	PN
	Risk-of- bias judgement			**Low risk of bias**
	**Overall risk of bias score**			**Low risk of bias**
Garcia-Ortiz (2018)	Risk of bias arising from the randomization process	Was the allocation sequence random?	"The activity monitors' serial identification numbers were used to randomize the participants into two study arms. Each monitor's serial identification number was assigned to either Game or Control group, matching the number of enrolled children."<br><br>Not clear if the sequence was truly random.	NI
		Was the allocation sequence concealed until participants were enrolled and assigned to interventions?	"A research assistant, blinded to the randomization, concealed the serial identification numbers in opaque envelopes, in randomized blocks of four. The randomization was performed separately for each class."	Yes

		Did baseline differences between intervention groups suggest a problem with the randomization process?	"A two-way t-test showed no significant difference between the Game and Control groups' demographic features and baseline physical activity"	No
	Risk-of- bias judgement			**Low risk of bias**
	Risk of bias due to deviations from the intended interventions	Were participants aware of their assigned intervention during the trial?	No information	NI
		Were carers and people delivering the interventions aware of participants' assigned intervention during the trial?	No information	NI
		Were there deviations from the intended intervention that arose because of the trial context?	No information	NI
		Were these deviations likely to have affected the outcome?	NA	NA
		Were these deviations from intended intervention balanced between groups?	NA	NA
		Was an appropriate analysis used to estimate the effect of assignment to intervention?	mITT<br><br>"As recommended by adolescent physical activity guidelines, a period of 60 consecutive minutes with zero steps was considered	Yes

			‘‘non-wear time’’. Only days with over 10 hours of ‘‘wear time’’ were included in analyses, and a minimum of 3 ‘‘wear days’’ per week were required to estimate the average number of steps and active minutes per day within each study week.” (37/42 analyzed)	
		Was there potential for a substantial impact (on the result) of the failure to analyse participants in the group to which they were randomized?	NA	NA
	Risk-of- bias judgement			**Some concerns**
	Risk of bias due to missing outcome data	Were data for this outcome available for all, or nearly all, participants randomized?	“One participant withdrew from the study due to illness and four did not meet the minimum ‘‘wear-time’’ criteria across the entire study. Thus, the physical activity of 37 participants out of the 42-participant cohort was studied.” (37/42 = 88.1%)	PN
		Is there evidence that the result was not biased by missing outcome data?		No
		Could missingness in the outcome depend on its true value?	Missing outcome of four participants that did not meet the	PY

			minimum “wear-time” could depend on its true value	
		Is it likely that missingness in the outcome depended on its true value?	Differences between participant groups in the proportions of missing outcome data is similar	PN
	Risk-of- bias judgement			**Some concerns**
	Risk of bias in measurement of the outcome	Was the method of measuring the outcome inappropriate?	“Tractivity is a triaxial accelerometer-based wearable device that measures steps and active minutes in 1-minute epochs. It has been previously validated in several studies and is suitable for measuring indoor and outdoor activity and, while not waterproof, can tolerate sweat and rain.”	No
		Could measurement or ascertainment of the outcome have differed between intervention groups?	Same method of measurement in all conditions	No
		Were outcome assessors aware of the intervention received by study participants?	No information	NI
		Could assessment of the outcome have been influenced by knowledge of intervention received?	Assessment of outcome utilize objective measures, involving little to no judgement	No
		Is it likely that assessment of the outcome was influenced by	NA	NA

		knowledge of intervention received?		
	Risk-of- bias judgement			**Low risk of bias**
	Risk of bias in selection of the reported result	Were the data that produced this result analysed in accordance with a pre-specified analysis plan that was finalized before unblinded outcome data were available for analysis?	Study protocol not available.	NI
		Is the numerical result being assessed likely to have been selected, on the basis of the results, from multiple eligible outcome measurements (e.g. scales, definitions, time points) within the outcome domain?	Mean (SD) for steps only presented at baseline (PA across weeks relative to baseline presented in boxplot though). Mean difference presented.	No
		Is the numerical result being assessed likely to have been selected, on the basis of the results, from multiple eligible analyses of the data?	all eligible reported results for the outcome domain correspond to all intended outcome measurements.	No
	Risk-of- bias judgement			**Some concerns**
	**Overall risk of bias score**			**High risk of bias**
Garde (2018)	Risk of bias arising from the randomization process	Was the allocation sequence random?	"The activity monitors' serial identification numbers were used to	NI

			randomize the participants into two study arms. Each monitor's serial identification number was assigned to either Game or Control group, matching the number of enrolled children."<br><br>Not clear if the sequence was truly random.	
		Was the allocation sequence concealed until participants were enrolled and assigned to interventions?	"A research assistant, blinded to the randomization, concealed the serial identification numbers in opaque envelopes, in randomized blocks of four. The randomization was performed separately for each class."	Yes
		Did baseline differences between intervention groups suggest a problem with the randomization process?	"A two-way t-test showed no significant difference between the Game and Control groups' demographic features and baseline physical activity"	No
	Risk-of- bias judgement			**Low risk of bias**
	Risk of bias due to deviations from the intended interventions	Were participants aware of their assigned intervention during the trial?	No information	NI
		Were carers and people delivering the interventions aware of participants' assigned intervention during the trial?	No information	NI

		Were there deviations from the intended intervention that arose because of the trial context?	No information	NI
		Were these deviations likely to have affected the outcome?	NA	NA
		Were these deviations from intended intervention balanced between groups?	NA	NA
		Was an appropriate analysis used to estimate the effect of assignment to intervention?	mITT<br><br>"As recommended by adolescent physical activity guidelines, a period of 60 consecutive minutes with zero steps was considered ''non-wear time''. Only days with over 10 hours of ''wear time'' were included in analyses, and a minimum of 3 ''wear days'' per week were required to estimate the average number of steps and active minutes per day within each study week." (37/42 analyzed)	Yes
		Was there potential for a substantial impact (on the result) of the failure to analyse participants in the group to which they were randomized?	NA	NA
	Risk-of- bias judgement			**Some concerns**

	Risk of bias due to missing outcome data	Were data for this outcome available for all, or nearly all, participants randomized?	“One participant withdrew from the study due to illness and four did not meet the minimum ‘‘wear-time’’ criteria across the entire study. Thus, the physical activity of 37 participants out of the 42-participant cohort was studied.” (37/42 = 88.1%)	PN
		Is there evidence that the result was not biased by missing outcome data?		No
		Could missingness in the outcome depend on its true value?	Missing outcome of four participants that did not meet the minimum “wear-time” could depend on its true value	PY
		Is it likely that missingness in the outcome depended on its true value?	Differences between participant groups in the proportions of missing outcome data is similar	PN
	Risk-of- bias judgement			**Some concerns**
	Risk of bias in measurement of the outcome	Was the method of measuring the outcome inappropriate?	“Tractivity is a triaxial accelerometer-based wearable device that measures steps and active minutes in 1-minute epochs. It has been previously validated in several studies and is suitable for measuring indoor and outdoor	No

			activity and, while not waterproof, can tolerate sweat and rain."	
		Could measurement or ascertainment of the outcome have differed between intervention groups?	Same method of measurement in all conditions	No
		Were outcome assessors aware of the intervention received by study participants?	No information	NI
		Could assessment of the outcome have been influenced by knowledge of intervention received?	Assessment of outcome utilize objective measures, involving little to no judgement	No
		Is it likely that assessment of the outcome was influenced by knowledge of intervention received?	NA	NA
	Risk-of- bias judgement			**Low risk of bias**
	Risk of bias in selection of the reported result	Were the data that produced this result analysed in accordance with a pre-specified analysis plan that was finalized before unblinded outcome data were available for analysis?	Study protocol not available.	NI
		Is the numerical result being assessed likely to have been selected, on the basis of the results, from multiple eligible outcome	Mean (SD) for steps only presented at baseline (PA across weeks relative to baseline presented in	No

		measurements (e.g. scales, definitions, time points) within the outcome domain?	boxplot though). Mean difference presented.	
		Is the numerical result being assessed likely to have been selected, on the basis of the results, from multiple eligible analyses of the data?	all eligible reported results for the outcome domain correspond to all intended outcome measurements.	No
	Risk-of- bias judgement			**Some concerns**
	**Overall risk of bias score**			**High risk of bias**
Glynn (2014)	Risk of bias arising from the randomization process	Was the allocation sequence random?	"Randomisation occurred using random permuted blocks to ensure there were similar numbers of participants in the intervention and control groups. An independent investigator was responsible or generating the allocation sequence using the Research Randomizer computer software program (available at www.randomizer.org/form.htm) The same independent investigator was responsible for assigning participants to the intervention and control groups after being called at a central site."	Yes
		Was the allocation sequence concealed until participants were enrolled and assigned to interventions?	"In this way, the allocation sequence was concealed from all study investigators and participants until all codes were assigned and week 1 was completed."	Yes

		Did baseline differences between intervention groups suggest a problem with the randomization process?	"There were no significant differences between control and intervention groups at baseline except for sex (P= 0.008) and one measure of quality of life (EQ-5D, P= 0.03)."<br><br>"In addition, there was a difference in baseline step count between control and intervention groups. This was not statistically significant but, nonetheless, this potential difference was recognised a priori and adjusted for in the final statistical model"	PY
	Risk-of- bias judgement			**Some concerns**
	Risk of bias due to deviations from the intended interventions	Were participants aware of their assigned intervention during the trial?	"During week 1, the smartphone app display was not visible for either group and the investigators remained blinded. At the end of week 1, the randomisation code was broken by the investigators."<br><br>Blinding probably only during Baseline period	PY
		Were carers and people delivering the interventions aware of participants' assigned intervention during the trial?	"During week 1, the smartphone app display was not visible for either group and the investigators	PY

			remained blinded. At the end of week 1, the randomisation code was broken by the investigators."<br><br>Blinding probably only during Baseline period	
		Were there deviations from the intended intervention that arose because of the trial context?	"The full study protocol has been published elsewhere and the study was carried out without any deviations from the protocol."	No
		Were these deviations likely to have affected the outcome?	NA	NA
		Were these deviations from intended intervention balanced between groups?	NA	NA
		Was an appropriate analysis used to estimate the effect of assignment to intervention?	"All analyses will be conducted according to the intention-to-treat principle" (77/90 = 86% analysed) mITT performed.	Yes
		Was there potential for a substantial impact (on the result) of the failure to analyse participants in the group to which they were randomized?	NA	NA
	Risk-of- bias judgement			**Low risk of bias**
	Risk of bias due to missing outcome data	Were data for this outcome available for all, or nearly all, participants randomized?	"Finally, due to the 'sleep' function on certain smartphone models, which forced the app to pause,	PN

			some step-count data were not recorded' this is why such data were not available for all participants at follow-up. However, this was similar for both groups over the course of the trial and was accounted for in the statistical modelling."	
		Is there evidence that the result was not biased by missing outcome data?	"Analysis of missing data suggested they were missing at random and were, therefore, accounted for in the mixed model; the validity of this assumption was investigated by looking at the missing-data patterns and by modelling the probability of missing data based on the explanatory variables available."	Yes
		Could missingness in the outcome depend on its true value?	NA	NA
		Is it likely that missingness in the outcome depended on its true value?	NA	NA
	Risk-of- bias judgement			**Low risk of bias**
	Risk of bias in measurement of the outcome	Was the method of measuring the outcome inappropriate?	Accupedo-Pro Pedometer app; "Pedometers containing accelerometers are well established as an acceptable form of	PN

			monitoring and have been shown to be successful in promoting physical activity”	
		Could measurement or ascertainment of the outcome have differed between intervention groups?	Same method of measurement in all conditions	No
		Were outcome assessors aware of the intervention received by study participants?	“Step-count data were recorded automatically, beyond the control of investigators and participants, and stored by the app on the telephones of all trial participants.”<br><br>No information about outcome assessor	NI
		Could assessment of the outcome have been influenced by knowledge of intervention received?	Assessment of outcome utilize objective measures, involving little to no judgement	No
		Is it likely that assessment of the outcome was influenced by knowledge of intervention received?	NA	NA
	Risk-of- bias judgement			**Low risk of bias**
	Risk of bias in selection of the reported result	Were the data that produced this result analysed in accordance with a pre-specified analysis plan that was finalized before unblinded	“The full study protocol has been published elsewhere and the study was carried out without any deviations from the protocol.”	Yes

		outcome data were available for analysis?		
		Is the numerical result being assessed likely to have been selected, on the basis of the results, from multiple eligible outcome measurements (e.g. scales, definitions, time points) within the outcome domain?	Mean (SD) for steps presented at all time points of measurement (Pre/Post). Adjusted / Unadjusted difference (95% CI) presented.	No
		Is the numerical result being assessed likely to have been selected, on the basis of the results, from multiple eligible analyses of the data?	There is clear evidence (examination of a trial protocol) that all eligible reported results for the outcome domain correspond to all intended outcome measurements.	No
	Risk-of- bias judgement			**Low risk of bias**
	**Overall risk of bias score**			**Some concerns**
Gremaud (2018)	Risk of bias arising from the randomization process	Was the allocation sequence random?	"The investigators used sequentially numbered envelopes to implement the random allocation sequence. Two research assistants who enrolled all participants asked participants to select 1 of 2 sealed envelopes, giving them an equal chance (50%) of being selected for the intervention or control group."	Yes
		Was the allocation sequence concealed until participants were enrolled and assigned to interventions?	"The investigators used sequentially numbered envelopes to implement the random allocation sequence. Two research assistants	Yes

			who enrolled all participants asked participants to select 1 of 2 sealed envelopes, giving them an equal chance (50%) of being selected for the intervention or control group.”	
		Did baseline differences between intervention groups suggest a problem with the randomization process?	“No significant between-group differences were observed for baseline characteristics”	No
	Risk-of- bias judgement			**Low risk of bias**
	Risk of bias due to deviations from the intended interventions	Were participants aware of their assigned intervention during the trial?	“Masking: none (open label)”	Yes
		Were carers and people delivering the interventions aware of participants' assigned intervention during the trial?	“Masking: none (open label)”	Yes
		Were there deviations from the intended intervention that arose because of the trial context?	“During week 6 of the intervention, a bug with our platform occurred that resulted in MT participants receiving a high volume of text messages on a single day (due to the change from daylight savings to standard time). Subsequently, we stopped sending messages for 1 week and then resumed. This had a significant impact on participants’ Fitbit compliance rates during week 6 (see Figure 4). However, once the	Yes

			intervention resumed, compliance rates appear to have returned to prebug levels.	
		Were these deviations likely to have affected the outcome?	“In a sensitivity analysis considering only the data collected before the bug, the effect of MapTrek was +2071 steps per day (95% CI, 917–3216), a difference of 112 steps per day from the model using all of the data. These estimates (2071 steps per day versus 2183 steps per day) are clinically identical, and their 95% CIs both exclude 0 and include the alternative estimate. As a result, the interpretation of the data collected until the bug occurred and the entire data set are functionally identical.”	No
		Were these deviations from intended intervention balanced between groups?	NA	NA
		Was an appropriate analysis used to estimate the effect of assignment to intervention?	“A total of 144 participants were randomized to 1 of 2 groups: FB (N=72); or MT (N=72). Final analyses were completed on all 144 participants.”<br><br>ITT stated but actually mITT performed (excluded from analysis as not provided data or didn't want their data to be used)<br>(144/146 analysed)	Yes

		Was there potential for a substantial impact (on the result) of the failure to analyse participants in the group to which they were randomized?	NA	NA
	Risk-of- bias judgement			**Some concerns**
	Risk of bias due to missing outcome data	Were data for this outcome available for all, or nearly all, participants randomized?	"At baseline, after accounting for within-subject clustering, there were data for 88.7% (95% CI, 84.1–93.4) of the subject days in the FB arm. Compliance was 5.0 percentage points higher in MT, although this effect was not significant (95% CI, 1.4 to 11.5). After the start of the intervention, compliance was only 64.6% (95% CI, 58.2–71.0) among those in the FB arm, and the MT arm compliance was 16.5 percentage points higher (95% CI, 7.6–25.4)."	No
		Is there evidence that the result was not biased by missing outcome data?	"when we accounted for percent compliance within the daily step counts analysis, the effect of MapTrek slightly decreased from 2182.6 steps per day to 2119 steps per day (95% CI, 1038.0–3368.5). Therefore, because compliance did not have a large effect on MapTrek effectiveness, we believe this provides more confidence that the	PY

			MapTrek intervention was effective at increasing physical activity"	
		Could missingness in the outcome depend on its true value?	NA	NA
		Is it likely that missingness in the outcome depended on its true value?	NA	NA
	Risk-of- bias judgement			**Low risk of bias**
	Risk of bias in measurement of the outcome	Was the method of measuring the outcome inappropriate?	"The Fitbit Zip has been demonstrated as a valid measure of daily steps when compared to the Actigraph GT3X+ (r=0.99; intraclass correlation=0.98) in free living conditions."	PN
		Could measurement or ascertainment of the outcome have differed between intervention groups?	Same method of measurement in all conditions	No
		Were outcome assessors aware of the intervention received by study participants?	"Masking: none (open label)"	Yes
		Could assessment of the outcome have been influenced by knowledge of intervention received?	Assessment of outcome utilize objective measures, involving little to no judgement	No

		Is it likely that assessment of the outcome was influenced by knowledge of intervention received?	NA	NA
	Risk-of- bias judgement			**Low risk of bias**
	Risk of bias in selection of the reported result	Were the data that produced this result analysed in accordance with a pre-specified analysis plan that was finalized before unblinded outcome data were available for analysis?	No Study protocol available.<br><br>Proclaimed method of analysis in IRB directions: ANCOVA.<br><br>Performed method of analysis: mixed-effect model	PN
		Is the numerical result being assessed likely to have been selected, on the basis of the results, from multiple eligible outcome measurements (e.g. scales, definitions, time points) within the outcome domain?	Mean (SD) for MVPA presented at all time-points of measurement (Pre/Post). Mean difference (95% CI) presented.	No
		Is the numerical result being assessed likely to have been selected, on the basis of the results, from multiple eligible analyses of the data?	All eligible reported results for the outcome measurement correspond to all intended analyses stated in the original research paper. (analysis differs from intended analysis stated IRB direction though)	PN
	Risk-of- bias judgement			**Some concerns**

	**Overall risk of bias score**			**Some concerns**
Harries (2016)	Risk of bias arising from the randomization process	Was the allocation sequence random?	"To ensure random allocation of participants, they were listed in the order in which they had been recruited and each third participant in the list was allocated to one of the three groups. This process was undertaken manually by a research-team member who had not had any contact with the participants, and was therefore blinded in relation to other details of the participants."<br><br>No random element was used in generating the allocation sequence / the sequence is predictable.	PN
		Was the allocation sequence concealed until participants were enrolled and assigned to interventions?	"This process was undertaken manually by a research-team member who had not had any contact with the participants, and was therefore blinded in relation to other details of the participants."	Yes
		Did baseline differences between intervention groups suggest a problem with the randomization process?	no useful baseline information available (study reported only baseline characteristics of participants in the final analysis)	No information
	Risk-of- bias judgement			**Some concerns**

	Risk of bias due to deviations from the intended interventions	Were participants aware of their assigned intervention during the trial?	“Participants were blinded in that all three groups had a similar looking icon on their phone, although access to the data in the app was not visible to participants in the control group.”	PY
		Were carers and people delivering the interventions aware of participants' assigned intervention during the trial?	no information	NI
		Were there deviations from the intended intervention that arose because of the trial context?	no information	NI
		Were these deviations likely to have affected the outcome?	NA	NA
		Were these deviations from intended intervention balanced between groups?	NA	NA
		Was an appropriate analysis used to estimate the effect of assignment to intervention?	mITT<br><br>“Of the 165 original recruits, 161 participants completed the study in its entirety. No participants were excluded from the study for non-compliance. Nine participants were excluded from the statistical analysis because of missing demographic data. Two were unable to complete because their phone was damaged or stolen, one withdrew without giving a reason	PY

			and one gave data costs as the reason for withdrawal.” (152/165 = 92.12 % analyzed)	
		Was there potential for a substantial impact (on the result) of the failure to analyse participants in the group to which they were randomized?	NA	NA
	Risk-of- bias judgement			**Some concerns**
	Risk of bias due to missing outcome data	Were data for this outcome available for all, or nearly all, participants randomized?	“A total of 6214 observations were recorded over 42 days across the 152 subjects, with 92 % of the subjects (i.e., all but 13) providing observations on at least 40 days.”	Yes
		Is there evidence that the result was not biased by missing outcome data?	NA	NA
		Could missingness in the outcome depend on its true value?	NA	NA
		Is it likely that missingness in the outcome depended on its true value?	NA	NA
	Risk-of- bias judgement			**Low risk of bias**

	Risk of bias in measurement of the outcome	Was the method of measuring the outcome inappropriate?	bActive app: "For such apps, measurement accuracy is now considered less important than previously and the emphasis, instead, is on the design features of the app. Early research into pedometers emphasised the importance of measurement validation using gold standard methods such as calorie expenditure and oxygen consumption. Now, however, a lower standard of accuracy is generally accepted for apps aimed at influencing behaviour and emphasis is placed on interactive features such as goal-setting, behavioural feedback loops and features that combine motivation with enjoyment."	PN
		Could measurement or ascertainment of the outcome have differed between intervention groups?	Same method of measurement in all conditions	No
		Were outcome assessors aware of the intervention received by study participants?	No information	NI
		Could assessment of the outcome have been influenced by knowledge of intervention received?	Assessment of outcome utilize objective measures, involving little to no judgement	No

		Is it likely that assessment of the outcome was influenced by knowledge of intervention received?	NA	NA
	Risk-of- bias judgement			**Low risk of bias**
	Risk of bias in selection of the reported result	Were the data that produced this result analysed in accordance with a pre-specified analysis plan that was finalized before unblinded outcome data were available for analysis?	No study protocol available.<br><br>"The study was not registered with a research register, although we recognise that doing so is increasingly emerging as best practice."	NI
		Is the numerical result being assessed likely to have been selected, on the basis of the results, from multiple eligible outcome measurements (e.g. scales, definitions, time points) within the outcome domain?	Mean (SD) not presented at all time-points of measurement (daily step counts presented in figure though)/ Effect size for steps presented.	No
		Is the numerical result being assessed likely to have been selected, on the basis of the results, from multiple eligible analyses of the data?	All eligible reported results for the outcome measurement correspond to all intended analyses.	No
	Risk-of- bias judgement			**Some concerns**

	**Overall risk of bias score**			**High risk of bias**
Hurkmans (2018)	Risk of bias arising from the randomization process	Was the allocation sequence random?	"The principle investigator allocated the participants in the different groups by means of random number allocation in Excel"	Yes
		Was the allocation sequence concealed until participants were enrolled and assigned to interventions?	NI	NI
		Did baseline differences between intervention groups suggest a problem with the randomization process?	"There were no baseline differences between the four groups, except for gender (P=.02). In the combi group, significant more men were included."	Yes
	Risk-of- bias judgement			**High risk of bias**
	Risk of bias due to deviations from the intended interventions	Were participants aware of their assigned intervention during the trial?	"masking: single (participant)"	PN
		Were carers and people delivering the interventions aware of participants' assigned intervention during the trial?	"masking: single (participant)"	PY
		Were there deviations from the	NI	NI

		intended intervention that arose because of the trial context?		
		Were these deviations likely to have affected the outcome?	NA	NA
		Were these deviations from intended intervention balanced between groups?	NA	NA
		Was an appropriate analysis used to estimate the effect of assignment to intervention?	ITT (102/102 analyzed)	Yes
		Was there potential for a substantial impact (on the result) of the failure to analyse participants in the group to which they were randomized?	NA	NA
	Risk-of- bias judgement			**Some concerns**
	Risk of bias due to missing outcome data	Were data for this outcome available for all, or nearly all, participants randomized?	attrition n=21 (20.59%)<br><br>81/102 (79.41%).	PN
		Is there evidence that the result was not biased by missing outcome data?	"However, when using the data of our combi group, a sample size of 15 would have been required. Furthermore, to see whether the data of these dropouts affected our results, a per-protocol and an ITT analyses was performed,	Yes

			which showed no differences on the main outcomes”	
		Could missingness in the outcome depend on its true value?	NA	NA
		Is it likely that missingness in the outcome depended on its true value?	NA	NA
	Risk-of- bias judgement			**Low risk of bias**
	Risk of bias in measurement of the outcome	Was the method of measuring the outcome inappropriate?	ActiGraph, model wGT3X-BT.	No
		Could measurement or ascertainment of the outcome have differed between intervention groups?	Same method of measurement in all conditions	No
		Were outcome assessors aware of the intervention received by study participants?	“All measurements were taken by a blinded assessor”	No
		Could assessment of the outcome have been influenced by knowledge of intervention received?	NA	NA
		Is it likely that assessment of the outcome was influenced by knowledge of intervention received?	NA	NA

	Risk-of- bias judgement			**Low risk of bias**
	Risk of bias in selection of the reported result	Were the data that produced this result analysed in accordance with a pre-specified analysis plan that was finalized before unblinded outcome data were available for analysis?	Study protocol not available.	NI
		Is the numerical result being assessed likely to have been selected, on the basis of the results, from multiple eligible outcome measurements (e.g. scales, definitions, time points) within the outcome domain?	Mean (SD) for MVPA presented at baseline. Post mean difference presented	No
		Is the numerical result being assessed likely to have been selected, on the basis of the results, from multiple eligible analyses of the data?	"Furthermore, to see whether the data of these dropouts affected our results, a per-protocol and an ITT analyses was performed, which showed no differences on the main outcomes"	No
	Risk-of- bias judgement			**Some concerns**
	**Overall risk of bias score**			**High risk of bias**
King (2016)	Risk of bias arising from the randomization process	Was the allocation sequence random?	"Random assignment occurred through use of a computerized version of the Efron procedure by the study statistical analyst, who	Yes

			was blinded to participant allocation assignment. The Efron procedure, while not guaranteeing identical final participant allocation numbers across study arms, allows for reasonably balanced subject allocation throughout the entire recruitment and randomization period, which is particularly advantageous for studies with smaller sample sizes and multiple study arms”	
		Was the allocation sequence concealed until participants were enrolled and assigned to interventions?	“Random assignment occurred through use of a computerized version of the Efron procedure by the study statistical analyst, who was blinded to participant allocation assignment. The Efron procedure, while not guaranteeing identical final participant allocation numbers across study arms, allows for reasonably balanced subject allocation throughout the entire recruitment and randomization period, which is particularly advantageous for studies with smaller sample sizes and multiple study arms”	Yes
		Did baseline differences between intervention groups suggest a problem with the randomization process?	“No significant between-group baseline differences were found for the demographic variables, physical activity, or sedentary behavior variables measured via accelerometry or ecological	No

			momentary assessment (P values.15)."	
	Risk-of- bias judgement			**Low risk of bias**
	Risk of bias due to deviations from the intended interventions	Were participants aware of their assigned intervention during the trial?	"Masking: None (Open Label)"	Yes
		Were carers and people delivering the interventions aware of participants' assigned intervention during the trial?	"Masking: None (Open Label)"	Yes
		Were there deviations from the intended intervention that arose because of the trial context?	"All analyses were conducted using original group assignment. No adverse events or harms were found across the study period."	PN
		Were these deviations likely to have affected the outcome?	NA	NA
		Were these deviations from intended intervention balanced between groups?	NA	NA
		Was an appropriate analysis used to estimate the effect of assignment to intervention?	mITT (89/95 analysed)	Yes
		Was there potential for a substantial impact (on the result) of the failure to analyse participants in the group to which they were randomized?	NA	NA

	Risk-of- bias judgement			**Low risk of bias**
	Risk of bias due to missing outcome data	Were data for this outcome available for all, or nearly all, participants randomized?	n=4 dropped out due to insufficient data. 89/95 analysed (94%)	Yes
		Is there evidence that the result was not biased by missing outcome data?		NA
		Could missingness in the outcome depend on its true value?	NA	NA
		Is it likely that missingness in the outcome depended on its true value?	NA	NA
	Risk-of- bias judgement			**Low risk of bias**
	Risk of bias in measurement of the outcome	Was the method of measuring the outcome inappropriate?	"(....) ,measured via smartphone-based accelerometry, which was validated against Actigraph accelerometry"	No
		Could measurement or ascertainment of the outcome have	Same method of measurement in all conditions	No

		differed between intervention groups?		
		Were outcome assessors aware of the intervention received by study participants?	"study statistical analyst, who was blinded to participant allocation assignment"	No
		Could assessment of the outcome have been influenced by knowledge of intervention received?	NA	NA
		Is it likely that assessment of the outcome was influenced by knowledge of intervention received?	NA	NA
	Risk-of- bias judgement			**Low risk of bias**
	Risk of bias in selection of the reported result	Were the data that produced this result analysed in accordance with a pre-specified analysis plan that was finalized before unblinded outcome data were available for analysis?	Available study protocol not stating analysis plan.	NI
		Is the numerical result being assessed likely to have been selected, on the basis of the results, from multiple eligible outcome measurements (e.g. scales, definitions, time points) within the outcome domain?	Mean (SD) for MVPA presented only at baseline (changes in accelerometer-derived MVPA by Study Arm presented in figure). Mean difference presented	No

		Is the numerical result being assessed likely to have been selected, on the basis of the results, from multiple eligible analyses of the data?	All eligible reported results for the outcome measurement correspond to all intended analyses.	No
	Risk-of- bias judgement			**Some concerns**
	Overall risk of bias score			**Some concerns**
Kitagawa (2020)	Risk of bias arising from the randomization process	Was the allocation sequence random?	"Using a computer-generated random numbers table, the participants were randomly assigned to one of three groups"	Yes
		Was the allocation sequence concealed until participants were enrolled and assigned to interventions?	NI	NI
		Did baseline differences between intervention groups suggest a problem with the randomization process?	"No significant differences were found in the age and BMI among the three groups before the intervention."	PN
	Risk-of- bias judgement			**Some concerns**
	Risk of bias due to deviations from the intended interventions	Were participants aware of their assigned intervention during the trial?	masking: single (staff)	PY

		Were carers and people delivering the interventions aware of participants' assigned intervention during the trial?	“The staff who performed assessments or interventions were masked as to participants’ group assignments.”	No
		Were there deviations from the intended intervention that arose because of the trial context?	“None of the participants dropped out during the intervention, and all participants completed the measurements during the first and second weeks”	PN
		Were these deviations likely to have affected the outcome?	NA	NA
		Were these deviations from intended intervention balanced between groups?	NA	NA
		Was an appropriate analysis used to estimate the effect of assignment to intervention?	“All data were analyzed according to participants’ group allocation, i.e., on intention-to-treat basis.”	Yes
		Was there potential for a substantial impact (on the result) of the failure to analyse participants in the group to which they were randomized?	NA	NA
	Risk-of- bias judgement			**Low risk of bias**
	Risk of bias due to missing outcome data	Were data for this outcome available for all, or nearly all, participants randomized?	“None of the participants dropped out during the intervention, and all participants completed the measurements during the first and second weeks”	Yes

		Is there evidence that the result was not biased by missing outcome data?	NA	NA
		Could missingness in the outcome depend on its true value?	NA	NA
		Is it likely that missingness in the outcome depended on its true value?	NA	NA
	Risk-of- bias judgement			**Low risk of bias**
	Risk of bias in measurement of the outcome	Was the method of measuring the outcome inappropriate?	wristband-type accelerometer (UP24 by Jawbone)	PN
		Could measurement or ascertainment of the outcome have differed between intervention groups?	Same method of measurement in all conditions	No
		Were outcome assessors aware of the intervention received by study participants?	"The staff who performed assessments or interventions were masked as to participants' group assignments."	No
		Could assessment of the outcome have been influenced by knowledge of intervention received?	NA	NA
		Is it likely that assessment of the outcome was influenced by	NA	NA

		knowledge of intervention received?		
	Risk-of- bias judgement			**Low risk of bias**
	Risk of bias in selection of the reported result	Were the data that produced this result analysed in accordance with a pre-specified analysis plan that was finalized before unblinded outcome data were available for analysis?	Study protocol not available.	NI
		Is the numerical result being assessed likely to have been selected, on the basis of the results, from multiple eligible outcome measurements (e.g. scales, definitions, time points) within the outcome domain?	Mean (SD) for MVPA presented at all time-points of measurement. Cohen's f reported (time effect & group x time)	No
		Is the numerical result being assessed likely to have been selected, on the basis of the results, from multiple eligible analyses of the data?	All eligible reported results for the outcome measurement correspond to all intended analyses.	No
	Risk-of- bias judgement			**Some concerns**
	**Overall risk of bias score**			**Some concerns**
Leinonen (2017)	Risk of bias arising from the randomization process	Was the allocation sequence random?	"Blinded randomization was performed by an assistant who was	Yes

			neither involved in the trial nor in the data collection and analysis. Randomization was conducted based on a list of computer-generated random numbers in blocks of 10. Each participant received sequentially the next random assignment in the list."	
		Was the allocation sequence concealed until participants were enrolled and assigned to interventions?	"Blinded randomization was performed by an assistant who was neither involved in the trial nor in the data collection and analysis. Randomization was conducted based on a list of computer-generated random numbers in blocks of 10. Each participant received sequentially the next random assignment in the list."	Yes
		Did baseline differences between intervention groups suggest a problem with the randomization process?	"The baseline characteristics of the study participants were similar between the intervention and control groups."	No
	Risk-of- bias judgement			**Low risk of bias**
	Risk of bias due to deviations from the intended interventions	Were participants aware of their assigned intervention during the trial?	"masking: single (outcome assessor)"	Yes
		Were carers and people delivering the interventions aware of	"masking: single (outcome assessor)"	Yes

		participants' assigned intervention during the trial?		
		Were there deviations from the intended intervention that arose because of the trial context?	high non-adherence rate (n=5 withdrew (no reasons stated), n=137 did not participate in final measurements)<br><br>"Technical problems, in some degree immature user interface design, and fragmented functionalities were recognized as challenges for the perceived ease of use."<br><br>These technical deviations could have possibly affected user engagement (intervention not fully successfully implemented).	PY
		Were these deviations likely to have affected the outcome?		PY
		Were these deviations from intended intervention balanced between groups?	only IG confronted with technical problems (CG no app use)<br>n=79 (CG) n=63 (IG). Dropout in IG higher (possibily due to technical difficulties of app)	PN
		Was an appropriate analysis used to estimate the effect of assignment to intervention?	"Data will be analysed using both intention to treat and per protocol basis."<br><br>mITT performed<br>"Among completers with valid physical activity data (n=167) (...)"<br>167/496 analyzed	Yes

		Was there potential for a substantial impact (on the result) of the failure to analyse participants in the group to which they were randomized?	NA	NA
	Risk-of- bias judgement			**High risk of bias**
	Risk of bias due to missing outcome data	Were data for this outcome available for all, or nearly all, participants randomized?	"Among completers with valid physical activity data (n=167) (...)"<br>(167/496 = 33.67%)<br><br>"One major limitation of this study was the missing physical activity data, the amount of which increased toward the end of the trial. Physical activity data needed to be uploaded to the database at least once every 3 weeks by the study participants, otherwise older data were overwritten by new data. Hence, it may be that some participants in the intervention group considered the feedback given by the PA monitor itself to be enough, and thus, they probably did not see a need for providing their physical activity data to the service. The stored data showed that those participants who did not use the	No

			MOPOrtal service, did not upload the physical activity data at all during the trial.”	
		Is there evidence that the result was not biased by missing outcome data?		No
		Could missingness in the outcome depend on its true value?	“The stored data showed that those participants who did not use the MOPOrtal service, did not upload the physical activity data at all during the trial.”	PY
		Is it likely that missingness in the outcome depended on its true value?	Lost to Follow-Up differed between groups (no judgment possible on otherwise missing outcome data as it is not reported detailed enough)	PY
	Risk-of- bias judgement			**High risk of bias**
	Risk of bias in measurement of the outcome	Was the method of measuring the outcome inappropriate?	physical activity monitor, Polar Active (uniaxial accelerometer). Not mentioned which model? “wrist-worn watch-like monitor”<br><br>“While assessing energy expenditure, a high correlation has been found between Polar Active and the doubly labeled water technique (R=.86), as well as	PN

			between Polar Active prototype and indirect calorimetry (R=.987)”	
		Could measurement or ascertainment of the outcome have differed between intervention groups?	Same method of measurement in all conditions	No
		Were outcome assessors aware of the intervention received by study participants?	“Masking: Single (Outcomes Assessor)”	No
		Could assessment of the outcome have been influenced by knowledge of intervention received?	NA	NA
		Is it likely that assessment of the outcome was influenced by knowledge of intervention received?	NA	NA
	Risk-of- bias judgement			**Low risk of bias**
	Risk of bias in selection of the reported result	Were the data that produced this result analysed in accordance with a pre-specified analysis plan that was finalized before unblinded outcome data were available for analysis?	Study protocol available. No deviations	Yes
		Is the numerical result being assessed likely to have been selected, on the basis of the results, from multiple eligible outcome	Mean (SD) for MVPA only presented for baseline (graph presented though). mean change provided	No

		measurements (e.g. scales, definitions, time points) within the outcome domain?		
		Is the numerical result being assessed likely to have been selected, on the basis of the results, from multiple eligible analyses of the data?	mITT and per-protocol performed and reported	No
	Risk-of- bias judgement			**Low risk of bias**
	**Overall risk of bias score**			**High risk of bias**
Lyons (2017)	Risk of bias arising from the randomization process	Was the allocation sequence random?	"Randomization was carried out using sequentially numbered opaque sealed envelopes according to standard protocols"	Yes
		Was the allocation sequence concealed until participants were enrolled and assigned to interventions?	"Randomization was conducted using standard opaque envelopes with foil (to prevent seeing the group assignment inside the envelope) and carbon paper (to provide an audit trail). The envelopes were randomly sorted by an individual not involved with the randomization visit process,then numbered sequentially. As interventionists opened each envelope, they signed and dated each envelope and saved the inner paper with original printed allocation and carbon-copied sequence number, ID number of the participant,	Yes

			signature of interventionist, and date of opening."	
		Did baseline differences between intervention groups suggest a problem with the randomization process?	no useful baseline information available (study reported only baseline characteristics of participants in the final analysis)	NI
	Risk-of- bias judgement			**Low risk of bias**
	Risk of bias due to deviations from the intended interventions	Were participants aware of their assigned intervention during the trial?	"Participants could not be blinded to their group"	Yes
		Were carers and people delivering the interventions aware of participants' assigned intervention during the trial?	"Masking: Single (Outcomes Assessor)"	Yes
		Were there deviations from the intended intervention that arose because of the trial context?	"No discussion of changes were included because no substantive changes were made"	No
		Were these deviations likely to have affected the outcome?	NA	NA
		Were these deviations from intended intervention balanced between groups?	NA	NA
		Was an appropriate analysis used to estimate the effect of assignment to intervention?	"All the analyses used the intent-to-treat principle, bringing the last observation forward for the ones who dropped out."	Yes

		Was there potential for a substantial impact (on the result) of the failure to analyse participants in the group to which they were randomized?	NA	NA
	Risk-of- bias judgement			**Low risk of bias**
	Risk of bias due to missing outcome data	Were data for this outcome available for all, or nearly all, participants randomized?	NI. retention 38/40 (95%) but no missing outcomes reported	NI
		Is there evidence that the result was not biased by missing outcome data?	"Analyses conducted with only the study completers with complete data did not produce substantially different results"	Yes
		Could missingness in the outcome depend on its true value?	NA	NA
		Is it likely that missingness in the outcome depended on its true value?	NA	NA
	Risk-of- bias judgement			**Low risk of bias**

	Risk of bias in measurement of the outcome	Was the method of measuring the outcome inappropriate?	"The ActivPAL is well-validated for use in measuring physical activity as well as sedentary behavior"	No
		Could measurement or ascertainment of the outcome have differed between intervention groups?	Same method of measurement in all conditions	No
		Were outcome assessors aware of the intervention received by study participants?	"Unfortunately, resource limitations precluded using blinded assessors for all participants."	PY
		Could assessment of the outcome have been influenced by knowledge of intervention received?	Assessment of outcome utilize objective measures, involving little to no judgement	No
		Is it likely that assessment of the outcome was influenced by knowledge of intervention received?	NA	NA
	Risk-of- bias judgement			**Low risk of bias**
	Risk of bias in selection of the reported result	Were the data that produced this result analysed in accordance with a pre-specified analysis plan that was finalized before unblinded outcome data were available for analysis?	Study protocol available.<br><br>Study protocol:<br><br>"Minutes of moderate to vigorous intensity physical activity in a seven-day period is the primary outcome measure."	PY

			not reported in the study article (only active minutes). Explanation: Tool of measurement not capable of reporting MVPA. Changes to the analysis plan are likely to be made before unblinded data was available and therefore do not raise concerns.	
		Is the numerical result being assessed likely to have been selected, on the basis of the results, from multiple eligible outcome measurements (e.g. scales, definitions, time points) within the outcome domain?	Mean (SD) presented for pre and post. Cohen's d presented	No
		Is the numerical result being assessed likely to have been selected, on the basis of the results, from multiple eligible analyses of the data?	"Analyses conducted with only the study completers with complete data did not produce substantially different results (eg, effect sizes based on only complete data were 0.40 for minutes and 0.31 for steps as compared with 0.35 and 0.26, respectively, in the intent-to-treat analysis). Therefore, we have presented the results from the intent-to-treat analysis."	No
	Risk-of- bias judgement			**Low risk**
	**Overall risk of bias score**			**Low risk**
Martin (2015)	Risk of bias arising from the randomization process	Was the allocation sequence random?	"Generated random sequences in Stata 11.1 (College Station, TX)"	Yes

		Was the allocation sequence concealed until participants were enrolled and assigned to interventions?	“Allocation was concealed so it could not be foreseen in advance of, or during, enrollment.”	Yes
		Did baseline differences between intervention groups suggest a problem with the randomization process?	“There were no significant baseline differences between groups”	No
	Risk-of- bias judgement			**Low risk of bias**
	Risk of bias due to deviations from the intended interventions	Were participants aware of their assigned intervention during the trial?	“As described in the manuscript, all participants were blinded during the 1-week blinded run-in and the 16 participants in the blinded group were blinded throughout the trial.”	PY
		Were carers and people delivering the interventions aware of participants' assigned intervention during the trial?	“Care providers were also blinded during the trial and physical activity outcomes were objectively assessed by the activity tracker.”	No
		Were there deviations from the intended intervention that arose because of the trial context?	“There were no important changes to methods after trial commencement.”<br><br>“To the best of our knowledge, there were no critical secular events during the trial period.”	No
		Were these deviations likely to have affected the outcome?	NA	NA

		Were these deviations from intended intervention balanced between groups?	NA	NA
		Was an appropriate analysis used to estimate the effect of assignment to intervention?	”All outcomes were compared between treatment arms by intention to treat.”	Yes
		Was there potential for a substantial impact (on the result) of the failure to analyse participants in the group to which they were randomized?	NA	NA
	Risk-of- bias judgement			**Low risk of bias**
	Risk of bias due to missing outcome data	Were data for this outcome available for all, or nearly all, participants randomized?	“We monitored daily activity data capture, which throughout the study was 97.4%.”	Yes
		Is there evidence that the result was not biased by missing outcome data?	NA	NA
		Could missingness in the outcome depend on its true value?	NA	NA
		Is it likely that missingness in the outcome depended on its true value?	NA	NA

	Risk-of- bias judgement			**Low risk of bias**
	Risk of bias in measurement of the outcome	Was the method of measuring the outcome inappropriate?	Fitbug Orb<br><br>“To promote accuracy, we set each participants' individual stride length and selected stride extender to account for the longer stride length with jogging or running relative to walking. We conducted accuracy testing prior to trial enrollment, which showed the Fitbug Orb to be comparable to traditional pedometers, including the higherend Accusplit AH120MAG (n=40; r=0.99, p<0.001; mean 12,748 steps, CV 10.3%, mean± difference of Fitbug – Accusplit: -319±1316 steps).”	PN
		Could measurement or ascertainment of the outcome have differed between intervention groups?	Same method of measurement in all conditions	No
		Were outcome assessors aware of the intervention received by study participants?	“Masking: Double (Participant, Outcomes Assessor)”	No
		Could assessment of the outcome have been influenced by knowledge of intervention received?	NA	NA

		Is it likely that assessment of the outcome was influenced by knowledge of intervention received?	NA	NA
	Risk-of- bias judgement			**Low risk of bias**
	Risk of bias in selection of the reported result	Were the data that produced this result analysed in accordance with a pre-specified analysis plan that was finalized before unblinded outcome data were available for analysis?	Study protocol not available.<br><br>claimed that protocol was published on trial registry (no analysis methods reported though)	NI
		Is the numerical result being assessed likely to have been selected, on the basis of the results, from multiple eligible outcome measurements (e.g. scales, definitions, time points) within the outcome domain?	Mean (SD) for steps not presented for pre and post (percentage of participants with ≥10 000 steps/day presented though). Adjusted mean difference (95% CI) presented	No
		Is the numerical result being assessed likely to have been selected, on the basis of the results, from multiple eligible analyses of the data?	All eligible reported results for the outcome measurement correspond to all intended analyses.	No
	Risk-of- bias judgement			**Some concerns**
	**Overall risk of bias score**			**Some concerns**

Pope (2020)	Risk of bias arising from the randomization process	Was the allocation sequence random?	"(...) determined using a random numbers table with a 1:1 allocation ratio (...)"	Yes
		Was the allocation sequence concealed until participants were enrolled and assigned to interventions?	NI	NI
		Did baseline differences between intervention groups suggest a problem with the randomization process?	"No baseline group differences were observed for any variables aside from SB/day (t=2.624, p=0.012) and MVPA/day (t=-2.040, p=0.048). Specifically, the comparison group had higher MVPA/day versus the experimental group, but the experimental group demonstrated higher SB/day versus the comparison group."	Yes
	Risk-of- bias judgement			**High risk of bias**
	Risk of bias due to deviations from the intended interventions	Were participants aware of their assigned intervention during the trial?	"Given the real-world, behavioral nature of this study and the fact all study participants were at the same University, we were unable to ensure participants were completely blind to group allocation."	Yes
		Were carers and people delivering the interventions aware of participants' assigned intervention during the trial?	"The study was not blinded."	Yes

		Were there deviations from the intended intervention that arose because of the trial context?	NI	NI
		Were these deviations likely to have affected the outcome?	NA	NA
		Were these deviations from intended intervention balanced between groups?	NA	NA
		Was an appropriate analysis used to estimate the effect of assignment to intervention?	"Comparisons of the two non-completers' baseline measurements with that of the 42 completers revealed no significant differences — providing rationale for inclusion of all participant data." (44/44 analyzed)	Yes
		Was there potential for a substantial impact (on the result) of the failure to analyse participants in the group to which they were randomized?	NA	NA
	Risk-of- bias judgement			**Some concerns**
	Risk of bias due to missing outcome data	Were data for this outcome available for all, or nearly all, participants randomized?	"Two participants did not complete the 10-week trial (95.5% retention rate)."<br><br>"If the participant had less than 10 hours/day of valid wear time on any day, these data were excluded from the analyses. Notably, as the	PY

			most representative habitual PA assessment was desired, we requested that the participants wear the accelerometer for at least three weekdays and two weekend days, with individual data not meeting this standard excluded.”	
		Is there evidence that the result was not biased by missing outcome data?		No
		Could missingness in the outcome depend on its true value?		NA
		Is it likely that missingness in the outcome depended on its true value?		NA
	Risk-of- bias judgement			**Low risk of bias**
	Risk of bias in measurement of the outcome	Was the method of measuring the outcome inappropriate?	“Actigraph GT3X+ accelerometers have been observed valid for PA assessment among adults in free-living conditions.”	No
		Could measurement or ascertainment of the outcome have differed between intervention groups?	Same method of measurement in all conditions	No

		Were outcome assessors aware of the intervention received by study participants?	"The study was not blinded."	Yes
		Could assessment of the outcome have been influenced by knowledge of intervention received?	Assessment of outcome utilize objective measures, involving little to no judgement	No
		Is it likely that assessment of the outcome was influenced by knowledge of intervention received?	NA	NA
	Risk-of- bias judgement			**Low risk of bias**
	Risk of bias in selection of the reported result	Were the data that produced this result analysed in accordance with a pre-specified analysis plan that was finalized before unblinded outcome data were available for analysis?	Study protocol not available.	NI
		Is the numerical result being assessed likely to have been selected, on the basis of the results, from multiple eligible outcome measurements (e.g. scales, definitions, time points) within the outcome domain?	Mean (SD) for MVPA presented at all time-points of measurement. no effect size presented (no inferential statistics performed)	No
		Is the numerical result being assessed likely to have been selected, on the basis of the results,	All eligible reported results for the outcome measurement correspond to all intended analyses.	No

		from multiple eligible analyses of the data?		
	Risk-of- bias judgement			**Some concerns**
	**Overall risk of bias score**			**High risk of bias**
Recio-Rodriguez (2016)	Risk of bias arising from the randomization process	Was the allocation sequence random?	"The rest of the subjects will be randomised on a centralized basis from Salamanca using the Epidat 4.0 software package to intervention (IG) and control groups (CG) with a ratio of 1/1."	Yes
		Was the allocation sequence concealed until participants were enrolled and assigned to interventions?	"The rest of the subjects will be randomised on a centralized basis from Salamanca using the Epidat 4.0 software package to intervention (IG) and control groups (CG) with a ratio of 1/1."	Yes
		Did baseline differences between intervention groups suggest a problem with the randomization process?	"no differences were observed between the two groups"	No
	Risk-of- bias judgement			**Low risk of bias**
	Risk of bias due to deviations from the intended interventions	Were participants aware of their assigned intervention during the trial?	"The nature of the intervention precludes blinding of the participants"	Yes

		Were carers and people delivering the interventions aware of participants' assigned intervention during the trial?	"Masking: Single Investigator"	PY
		Were there deviations from the intended intervention that arose because of the trial context?	NI	NI
		Were these deviations likely to have affected the outcome?	NA	NA
		Were these deviations from intended intervention balanced between groups?	NA	NA
		Was an appropriate analysis used to estimate the effect of assignment to intervention?	"Analysis of the results was made on an intention-to-treat basis." (833/833 analyzed)	Yes
		Was there potential for a substantial impact (on the result) of the failure to analyse participants in the group to which they were randomized?	NA	NA
	Risk-of- bias judgement			**Some concerns**
	Risk of bias due to missing outcome data	Were data for this outcome available for all, or nearly all, participants randomized?	NI. But 68/833 subjects dropped out (8.2%)	NI

		Is there evidence that the result was not biased by missing outcome data?		No
		Could missingness in the outcome depend on its true value?	"Lastly, the recorded loss rate of close to 10% may have biased the study sample composition to some extent because certain populations may have experienced difficulties using the app and consequently decided to leave the study."	Yes
		Is it likely that missingness in the outcome depended on its true value?	From a total of 833 subjects, 8.2% (68/833) dropped out, 8.7% (36/415) in the IG group and 7.7% (32/418) in the CG group.<br><br>Differences between participant groups in the proportions of missing outcome data (based on drop outs only) is similar	PN
	Risk-of- bias judgement			**Some concerns**
	Risk of bias in measurement of the outcome	Was the method of measuring the outcome inappropriate?	ActiGraph GT3X accelerometer	No
		Could measurement or ascertainment of the outcome have differed between intervention groups?	Same method of measurement in all conditions	No

		Were outcome assessors aware of the intervention received by study participants?	“The investigator who performed the data analysis was blinded”	No
		Could assessment of the outcome have been influenced by knowledge of intervention received?	NA	NA
		Is it likely that assessment of the outcome was influenced by knowledge of intervention received?	NA	NA
	Risk-of- bias judgement			**Low risk of bias**
	Risk of bias in selection of the reported result	Were the data that produced this result analysed in accordance with a pre-specified analysis plan that was finalized before unblinded outcome data were available for analysis?	Study protocol available.	Yes
		Is the numerical result being assessed likely to have been selected, on the basis of the results, from multiple eligible outcome measurements (e.g. scales, definitions, time points) within the outcome domain?	Mean (SD) for MVPA only for baseline presented. Mean difference presented	No
		Is the numerical result being assessed likely to have been selected, on the basis of the results,	ITT + per protocol performed and reported	No

		from multiple eligible analyses of the data?		
	Risk-of- bias judgement			**Low risk of bias**
	**Overall risk of bias score**			**Some concerns**
Robertson (2018)	Risk of bias arising from the randomization process	Was the allocation sequence random?	"After schools agreed to take part in the study, they were randomised to treatment by a staff member of the Scottish Collaboration for Public Health Research and Policy who was not involved in the project, using a computerised random number generator"	Yes
		Was the allocation sequence concealed until participants were enrolled and assigned to interventions?	"After schools agreed to take part in the study, they were randomised to treatment by a staff member of the Scottish Collaboration for Public Health Research and Policy who was not involved in the project, using a computerised random number generator"	Yes
		Did baseline differences between intervention groups suggest a problem with the randomization process?	"Once again the pre-test step count and minutes are comparable between control and FitQuest groups"	No
	Risk-of- bias judgement			**Low risk of bias**

	Risk of bias due to deviations from the intended interventions	Were participants aware of their assigned intervention during the trial?	“It was not possible to blind the participants to the intervention. Children and teachers were informed of the aims of the study.”	Yes
		Were carers and people delivering the interventions aware of participants' assigned intervention during the trial?	“It was not possible to blind the participants to the intervention. Children and teachers were informed of the aims of the study.”	Yes
		Were there deviations from the intended intervention that arose because of the trial context?	“While all the intervention schools did use FitQuest on at least two sessions, on average the schools used FitQuest for only 35% of the recommended time (103 min over 5 weeks). The reasons for this included poor weather but also motivational and contextual factors. Therefore, it is not possible to draw conclusions from this study about the efficacy of the FitQuest intervention as originally designed.”<br><br>“However, due to the limitation in the number of mobile devices,only half of class could use FitQuest at any given time.The one-hour lessons were split into two 30-min play sessions.”<br><br>“The schools were invited to allow the children to play the game during play time and lunchtime but as this was at the discretion of the head teacher, only one school did so.”	Yes

		Were these deviations likely to have affected the outcome?	“Therefore, it is not possible to draw conclusions from this study about the efficacy of the FitQuest intervention as originally designed.”	Yes
		Were these deviations from intended intervention balanced between groups?	“The schools were invited to allow the children to play the game during play time and lunchtime but as this was at the discretion of the head teacher, only one school did so.”<br><br>“It is worth noting that there was very poor weather during the post-test week for two of the schools which meant that the children were unable to play outside during some of the lunch and playtime activities.”	No
		Was an appropriate analysis used to estimate the effect of assignment to intervention?	mITT<br><br>“Participants who were missing data from more than 2 of 4 days were excluded from step count and MVPA analysis.”<br>(157/215 analysed)	PY
		Was there potential for a substantial impact (on the result) of the failure to analyse participants in the group to which they were randomized?	NA	NA
	Risk-of- bias judgement			**High risk of bias**

	Risk of bias due to missing outcome data	Were data for this outcome available for all, or nearly all, participants randomized?	"Participants who were missing data from more than 2 of 4 days were excluded from step count and MVPA analysis."<br>157/215 analyzed (73.02%)	No
		Is there evidence that the result was not biased by missing outcome data?	"Using the methods in Kang's analysis of children's step counts during the school day (Kang & Brinthaupt, 2009), we used the following approach to replacing missing primary outcome data. Data was gathered from 4 full school days; participants who were missing data from more than 2 of these days were excluded from step count and MVPA analysis. The daily step count for remaining participants was screened to replace values lower than 500 steps and higher than 15000 steps with the mean step count for the remaining days from the same participant. MVPA values were also replaced with the participant mean for the other days in cases where the step counts were considered invalid, or the MVPA value was missing. A total of 12 cases of missing values were replaced."<br><br>"Undertaking likelihood ratio tests identified that accounting for	No

			school clustering improved the fit of the models for each outcome.”	
		Could missingness in the outcome depend on its true value?	The circumstances of the trial make it likely that missingness in the outcome depends on its true value.	PY
		Is it likely that missingness in the outcome depended on its true value?	The circumstances of the trial make it likely that missingness in the outcome depends on its true value.	PY
	Risk-of- bias judgement			**High risk of bias**
	Risk of bias in measurement of the outcome	Was the method of measuring the outcome inappropriate?	“The NL-1000 provides an inexpensive method for assessing PA and has demonstrated acceptable concurrent validity when assessing MVPA in children aged 10-13 years”	No
		Could measurement or ascertainment of the outcome have differed between intervention groups?	Same method of measurement in all conditions	No
		Were outcome assessors aware of the intervention received by study participants?	“Data collectors were not blinded to the study aims and hypotheses as they were core members of the research team who assisted in the design of the study.”	Yes
		Could assessment of the outcome have been influenced by knowledge of intervention received?	Assessment of outcome utilize objective measures, involving little to no judgement	No

		Is it likely that assessment of the outcome was influenced by knowledge of intervention received?	NA	NA
	Risk-of- bias judgement			**Low risk of bias**
	Risk of bias in selection of the reported result	Were the data that produced this result analysed in accordance with a pre-specified analysis plan that was finalized before unblinded outcome data were available for analysis?	No study protocol available.	NI
		Is the numerical result being assessed likely to have been selected, on the basis of the results, from multiple eligible outcome measurements (e.g. scales, definitions, time points) within the outcome domain?	Mean (SD) presented for MVPA/steps at all time-points of measurement (baseline / 3 months / 9 months) / effect size presented for MVPA/steps	No
		Is the numerical result being assessed likely to have been selected, on the basis of the results, from multiple eligible analyses of the data?	all eligible reported results for the outcome domain correspond to all intended outcome measurements.	No
	Risk-of- bias judgement			**Some concerns**
	**Overall risk of bias score**			**High risk of bias**

Schade (2020)	Risk of bias arising from the randomization process	Was the allocation sequence random?	NI. "The participants were randomly assigned to either the Pokémon group, who played PG for the entirety of the study, or the control group, who refrained from playing PG."	NI
		Was the allocation sequence concealed until participants were enrolled and assigned to interventions?	NI. "The participants were randomly assigned to either the Pokémon group, who played PG for the entirety of the study, or the control group, who refrained from playing PG."	NI
		Did baseline differences between intervention groups suggest a problem with the randomization process?	"The subjects' age, body mass index, and resting heart rate act as a proxy as to their level of physical fitness prior to the study. Across these dimensions, no statistical difference was found between the 2 groups. this is taken to mean that neither group should have been more likely than the other to be more physically active."	PN
	Risk-of- bias judgement			**Some concerns**
	Risk of bias due to deviations from the intended interventions	Were participants aware of their assigned intervention during the trial?	"The subjects were naïve to the purpose of the experiment and given no instructions about their physical activity during the data collection."	PY

		Were carers and people delivering the interventions aware of participants' assigned intervention during the trial?	NI	NI
		Were there deviations from the intended intervention that arose because of the trial context?	NI.	NI
		Were these deviations likely to have affected the outcome?		NA
		Were these deviations from intended intervention balanced between groups?		NA
		Was an appropriate analysis used to estimate the effect of assignment to intervention?	mITT "Any days the Fitbit failed to collect data were not considered in the calculation of averages. For some participants, no step and/or distance data were recorded during the experiment. these subjects were not included in the comparison, thus producing the different sample sizes for the comparisons."	Yes
		Was there potential for a substantial impact (on the result) of the failure to analyse participants in the group to which they were randomized?	NA	NA
	Risk-of- bias judgement			**Some concerns**

	Risk of bias due to missing outcome data	Were data for this outcome available for all, or nearly all, participants randomized?	“For some participants, no step and/or distance data were recorded during the experiment. These subjects were not included in the comparison, thus producing the different sample sizes for the comparisons.”	PN
		Is there evidence that the result was not biased by missing outcome data?		No
		Could missingness in the outcome depend on its true value?	“Any days the Fitbit failed to collect data were not considered in the calculation of averages.“ Not clear why fitbit failed to collect data (e.g. due to participants not wearing it (therefore possibly depending on true value) or simply technical issues (therefore missing by chance))	PY
		Is it likely that missingness in the outcome depended on its true value?	intervention group n=11<br>control group n= 16<br>Dropout was likely higher in the experimental group.	PY
	Risk-of- bias judgement			**High risk of bias**
	Risk of bias in measurement of the outcome	Was the method of measuring the outcome inappropriate?	Fitbit Charge Heart Rate FB405BKL.	PN

		Could measurement or ascertainment of the outcome have differed between intervention groups?	Same method of measurement in all conditions	No
		Were outcome assessors aware of the intervention received by study participants?	NI	NI
		Could assessment of the outcome have been influenced by knowledge of intervention received?	Assessment of outcome utilize objective measures, involving little to no judgement	No
		Is it likely that assessment of the outcome was influenced by knowledge of intervention received?	NA	NA
	Risk-of- bias judgement			**Low risk of bias**
	Risk of bias in selection of the reported result	Were the data that produced this result analysed in accordance with a pre-specified analysis plan that was finalized before unblinded outcome data were available for analysis?	no study protocol available.	NI
		Is the numerical result being assessed likely to have been selected, on the basis of the results, from multiple eligible outcome measurements (e.g. scales, definitions, time points) within the outcome domain?	Mean (SD) for steps not presented at pre/post (shown in Boxplot). Hedges' g effect size presented	No

		Is the numerical result being assessed likely to have been selected, on the basis of the results, from multiple eligible analyses of the data?	All eligible reported results for the outcome measurement correspond to all intended analyses.	No
	Risk-of- bias judgement			**Some concerns**
	**Overall risk of bias score**			**High risk of bias**
Simons (2018)	Risk of bias arising from the randomization process	Was the allocation sequence random?	"Allocation was based on clusters (workplaces), which were randomly assigned following block randomization (restricted randomization) to the intervention or the control group. Block sizes varied randomly (2, 4, or 6), and for each block of clusters, half (1, 2,or 3) would be allocated to each arm of the study (intervention or control group)."	Yes
		Was the allocation sequence concealed until participants were enrolled and assigned to interventions?	No information	NI
		Did baseline differences between intervention groups suggest a problem with the randomization process?	"There was a gender difference between the 2 groups, with more males in the intervention group than in the control group."	Yes
	Risk-of- bias judgement			**High risk of bias**

	Risk of bias due to deviations from the intended interventions	Were participants aware of their assigned intervention during the trial?	"Masking: None (Open Label)"	Yes
		"Masking: None (Open Label)"	"Masking: None (Open Label)"	Yes
		Were there deviations from the intended intervention that arose because of the trial context?	No information	NI
		Were these deviations likely to have affected the outcome?	NA	NA
		Were these deviations from intended intervention balanced between groups?	NA	NA
		Was an appropriate analysis used to estimate the effect of assignment to intervention?	mITT<br><br>"Only data of participants with at least 10 wearing hours for at least 3 days (as recommended to reliably predict physical activity behavior in young adults) were included in the analyses."<br>(post-test: 102/130 analyzed follow-up 84/130 analyzed)	PY
		Was there potential for a substantial impact (on the result) of the failure to analyse participants in the group to which they were randomized?	NA	NA

	Risk-of- bias judgement			**Some concerns**
	Risk of bias due to missing outcome data	Were data for this outcome available for all, or nearly all, participants randomized?	"In the intervention group, (59/60) participants had valid accelerometer data at baseline, (48/60) at posttest, and (41/60) at follow-up."<br><br>"In the control group, (63/70) participants had valid data at baseline, (54/70) at posttest, and (43/70) at follow-up."	No
		Is there evidence that the result was not biased by missing outcome data?	"Generalized linear mixed models analyses allowed us to include all available measurements, even if participants completed only 1 or 2 measurements. Mixed models have advantages over fixed effects models in the treatment of missing values of the dependent variable. Mixed models are capable of handling the imbalance caused by missing observations and yield valid inferences if the missing observations are missing at random."	PY
		Could missingness in the outcome depend on its true value?	NA	NA

		Is it likely that missingness in the outcome depended on its true value?	NA	NA
	Risk-of- bias judgement			**Low risk of bias**
	Risk of bias in measurement of the outcome	Was the method of measuring the outcome inappropriate?	"physical activity was assessed objectively using Actigraph GT3X+ accelerometers. Both reliability and validity of Actigraph accelerometers have been documented extensively"	No
		Could measurement or ascertainment of the outcome have differed between intervention groups?	Same method of measurement in all conditions	No
		Were outcome assessors aware of the intervention received by study participants?	"Masking: None (Open Label)"	Yes
		Could assessment of the outcome have been influenced by knowledge of intervention received?	Assessment of outcome utilize objective measures, involving little to no judgement	No
		Is it likely that assessment of the outcome was influenced by knowledge of intervention received?	NA	NA
	Risk-of- bias judgement			**Low risk of bias**

	Risk of bias in selection of the reported result	Were the data that produced this result analysed in accordance with a pre-specified analysis plan that was finalized before unblinded outcome data were available for analysis?	No study protocol available.	NI
		Is the numerical result being assessed likely to have been selected, on the basis of the results, from multiple eligible outcome measurements (e.g. scales, definitions, time points) within the outcome domain?	Mean (SD) for MVPA presented at all time-points of measurement. No significant intervention effects detected.	No
		Is the numerical result being assessed likely to have been selected, on the basis of the results, from multiple eligible analyses of the data?	All eligible reported results for the outcome measurement correspond to all intended analyses.	No
	Risk-of- bias judgement			**Some concerns**
	**Overall risk of bias score**			**High risk of bias**
Walsh (2016)	Risk of bias arising from the randomization process	Was the allocation sequence random?	"Participants were then assigned to either the control or experimental group via block randomization (which was used to guarantee similar numbers within each condition)"	NI

		Was the allocation sequence concealed until participants were enrolled and assigned to interventions?	"During Week 1, the mobile phone app display was not visible to either group and the investigators remained blinded. (...) Following the collection of baseline data, the randomization code was broken by the investigators."	Yes
		Did baseline differences between intervention groups suggest a problem with the randomization process?	"Results of an independent samples t-test showed that there was no significant difference between daily step counts of the control and experimental groups at baseline, ensuring that randomization was effective."	No
	Risk-of- bias judgement			**Low risk of bias**
	Risk of bias due to deviations from the intended interventions	Were participants aware of their assigned intervention during the trial?	"During Week 1, the mobile phone app display was not visible to either group and the investigators remained blinded. (...) Following the collection of baseline data, the randomization code was broken by the investigators."	PY
		Were carers and people delivering the interventions aware of participants' assigned intervention during the trial?	"During Week 1, the mobile phone app display was not visible to either group and the investigators remained blinded. (...) Following the collection of baseline data, the randomization code was broken by the investigators."	PY

		Were there deviations from the intended intervention that arose because of the trial context?	56/58 did receive assigned intervention (1 app malfunction / 1 touchscreen malfunction). No deviations that arose because of the trial context.	No
		Were these deviations likely to have affected the outcome?	2/58 (3.75%)	PN
		Were these deviations from intended intervention balanced between groups?	n=1 IG / n=1 CG	Yes
		Was an appropriate analysis used to estimate the effect of assignment to intervention?	mITT<br><br>"Participants were only excluded due to technological issues (ie, incompatibility of mobile phone with the app or phone malfunction), therefore strengthening external validity." (55/58 analyzed)	Yes
		Was there potential for a substantial impact (on the result) of the failure to analyse participants in the group to which they were randomized?	NA	NA
	Risk-of- bias judgement			**Low risk**
	Risk of bias due to missing outcome data	Were data for this outcome available for all, or nearly all, participants randomized?	No information	NI

		Is there evidence that the result was not biased by missing outcome data?	"Analysis of missing data suggested that information was missing at random, and was therefore accounted for with Expectation Maximization substitution in the mixed model; the validity of this assumption was investigated by examining the missing data patterns and by modelling the probability of missing data based on the explanatory variables available."	Yes
		Could missingness in the outcome depend on its true value?	NA	NA
		Is it likely that missingness in the outcome depended on its true value?	NA	NA
	Risk-of- bias judgement			**Low risk of bias**
	Risk of bias in measurement of the outcome	Was the method of measuring the outcome inappropriate?	"The Accupedo-Pro Pedometer app was selected for use in the current study. Notably, this app obtained the highest ratings in previous comparisons of pedometer apps."	PN
		Could measurement or ascertainment of the outcome have differed between intervention groups?	Same method of measurement in all conditions	No

		Were outcome assessors aware of the intervention received by study participants?	No information	NI
		Could assessment of the outcome have been influenced by knowledge of intervention received?	Assessment of outcome utilize objective measures, involving little to no judgement	No
		Is it likely that assessment of the outcome was influenced by knowledge of intervention received?	NA	NA
	Risk-of- bias judgement			**Low risk of bias**
	Risk of bias in selection of the reported result	Were the data that produced this result analysed in accordance with a pre-specified analysis plan that was finalized before unblinded outcome data were available for analysis?	No study protocol available.	NI
		Is the numerical result being assessed likely to have been selected, on the basis of the results, from multiple eligible outcome measurements (e.g. scales, definitions, time points) within the outcome domain?	Mean (SD) for steps presented at all time-points of measurement. Effect size presented	No
		Is the numerical result being assessed likely to have been selected, on the basis of the results,	All eligible reported results for the outcome measurement correspond to all intended analyses.	No

		from multiple eligible analyses of the data?		
	Risk-of- bias judgement			**Some concerns**
	**Overall risk of bias score**			**Some concerns**
Zhang (2019)	Risk of bias arising from the randomization process	Was the allocation sequence random?	“We continuously assembled cohorts of eight women as recruitment proceeded. Women in each cohort were electronically randomized in a 1:1 ratio to the online group condition or the individual control condition. The four women randomized to the group condition became connected in an online group through the app. Participants and researchers were blind to the randomization process.”	Yes
		Was the allocation sequence concealed until participants were enrolled and assigned to interventions?	“We continuously assembled cohorts of eight women as recruitment proceeded. Women in each cohort were electronically randomized in a 1:1 ratio to the online group condition or the individual control condition. The four women randomized to the group condition became connected in an online group through the app. Participants and researchers were blind to the randomization process.”	Yes

		Did baseline differences between intervention groups suggest a problem with the randomization process?	no useful baseline information available (study reported only baseline characteristics of participants in the final analysis)	No information
	Risk-of- bias judgement			**Low risk of bias**
	Risk of bias due to deviations from the intended interventions	Were participants aware of their assigned intervention during the trial?	"Participants were blind to intervention condition before enrollment, thus avoiding differential self-selection bias."<br><br>not blinded after enrollment	PY
		Were carers and people delivering the interventions aware of participants' assigned intervention during the trial?	"Masking: Triple (Participant, Investigator, Outcomes Assessor)"	PN
		Were there deviations from the intended intervention that arose because of the trial context?	No information	NI
		Were these deviations likely to have affected the outcome?	NA	NA
		Were these deviations from intended intervention balanced between groups?	NA	NA
		Was an appropriate analysis used to estimate the effect of assignment to intervention?	"We performed the analyses using an intention-to-treat mode with participants analyzed based on their intervention assignment, regardless	Yes

			of the number of days of wearing the Fitbit or using the app.” (91/91 analyzed)	
		Was there potential for a substantial impact (on the result) of the failure to analyse participants in the group to which they were randomized?	NA	NA
	Risk-of- bias judgement			**Some concerns**
	Risk of bias due to missing outcome data	Were data for this outcome available for all, or nearly all, participants randomized?	“The total percentage of participant days on which Fitbit-tracked behavior data were missing during the 90-day intervention was 16%. Specifically, of the potential 8190 records of Fitbit-tracked behavior data, 1310 were missing. Intervention condition and time did not affect individuals’ rate of missing Fitbit data.”	No
		Is there evidence that the result was not biased by missing outcome data?	“We used GEE models to handle missing data, models that use all available data for each participant.”	Yes
		Could missingness in the outcome depend on its true value?	NA	NA
		Is it likely that missingness in the outcome depended on its true value?	NA	NA

	Risk-of- bias judgement			**Low risk of bias**
	Risk of bias in measurement of the outcome	Was the method of measuring the outcome inappropriate?	"The step data for the Fitbit Zip correlated highly with the step data yielded by the ActiGraph (r's=.72, .92, .96), and the MVPA data for the Fitbit Zip correlated highly with the MVPA data from the ActiGraph (r's=.67, .79, .94). Our data suggest that the Fitbit Zip is a reasonable alternative to the ActiGraph for estimating activity among free-living adolescents." (Schneider & Chau, 2016)	PN
		Could measurement or ascertainment of the outcome have differed between intervention groups?	Same method of measurement in all conditions	No
		Were outcome assessors aware of the intervention received by study participants?	"Masking: Triple (Participant, Investigator, Outcomes Assessor)"	No
		Could assessment of the outcome have been influenced by knowledge of intervention received?	NA	NA
		Is it likely that assessment of the outcome was influenced by knowledge of intervention received?	NA	NA

	Risk-of- bias judgement			**Low risk of bias**
	Risk of bias in selection of the reported result	Were the data that produced this result analysed in accordance with a pre-specified analysis plan that was finalized before unblinded outcome data were available for analysis?	No study protocol available.	NI
		Is the numerical result being assessed likely to have been selected, on the basis of the results, from multiple eligible outcome measurements (e.g. scales, definitions, time points) within the outcome domain?	Mean (SD) presented for all three measure periods. No effect sizes presented (There were no significant effects of intervention condition or time on daily steps, minutes of light activity, and minutes of MVPA.)	No
		Is the numerical result being assessed likely to have been selected, on the basis of the results, from multiple eligible analyses of the data?	all eligible reported results for the outcome domain correspond to all intended outcome measurements.	No
	Risk-of- bias judgement			**Some concerns**
	**Overall risk of bias score**			**Some concerns**
Zhou (2018)	Risk of bias arising from the randomization process	Was the allocation sequence random?	"All 64 participants were randomized to one of the 2 groups with a one-to-one ratio by a computer-based random number generator using the simple	Yes

			randomization approach. A one-to-one ratio means that each participant had a 50% probability of being assigned to one of the 2 groups, and the number of participants in each group may differ due to chance. The randomization to groups was implemented by the CalFit app after the run-in period, and the participants were aware of the 2 groups."	
		Was the allocation sequence concealed until participants were enrolled and assigned to interventions?	"This is not applicable since the app performed the randomization" (CONSORT)	Yes
		Did baseline differences between intervention groups suggest a problem with the randomization process?	"No baseline characteristics differed between the control and intervention groups."	No
	Risk-of- bias judgement			**Low risk of bias**
	Risk of bias due to deviations from the intended interventions	Were participants aware of their assigned intervention during the trial?	"The randomization to groups was implemented by the CalFit app after the run-in period, and the participants were aware of the 2 groups."<br><br>"Participants were blinded to group assignment initially, but they could	PY

			figure out their group assignment by the step goals they received" (	
		Were carers and people delivering the interventions aware of participants' assigned intervention during the trial?	"Masking: Double (Participant, Investigator)"	PN
		Were there deviations from the intended intervention that arose because of the trial context?	"There were no changes to methods after trial commencement."	No
		Were these deviations likely to have affected the outcome?	NA	NA
		Were these deviations from intended intervention balanced between groups?	NA	NA
		Was an appropriate analysis used to estimate the effect of assignment to intervention?	"Intention-to-treat analysis was performed for the primary and secondary outcomes, and per-protocol analysis was performed only for the primary outcome." (64/64 analyzed)	Yes
		Was there potential for a substantial impact (on the result) of the failure to analyse participants in the group to which they were randomized?	NA	NA
	Risk-of- bias judgement			**Low risk of bias**

	Risk of bias due to missing outcome data	Were data for this outcome available for all, or nearly all, participants randomized?	No information	NI
		Is there evidence that the result was not biased by missing outcome data?	"Sensitivity analysis was performed to obtain adjusted estimates of the effect of the treatment with the missing data on primary outcome, evaluated at P<.05. The primary cause of missing step data was failure to turn on the app. LMM implicitly imputes missing data by interpolation and is a common approach to deal with missing data in physical activity interventions. (We did not use the common imputation method of "last observation carried forward" because it would increase bias in this context and lead to potentially false conclusions by inflating step counts at 10 weeks."	Yes
		Could missingness in the outcome depend on its true value?	NA	NA
		Is it likely that missingness in the outcome depended on its true value?	NA	NA
	Risk-of- bias judgement			**Low risk of bias**

	Risk of bias in measurement of the outcome	Was the method of measuring the outcome inappropriate?	"The built-in health chip in the iPhone collects the step data, and the accuracy of step counts collected by the iPhone health chip has been validated in a number of studies to have comparable accuracy to an ActiGraph."	No
		Could measurement or ascertainment of the outcome have differed between intervention groups?	Same method of measurement in all conditions	No
		Were outcome assessors aware of the intervention received by study participants?	"Masking: Double (Participant, Investigator)"	Yes
		Could assessment of the outcome have been influenced by knowledge of intervention received?	Assessment of outcome utilize objective measures, not involving judgement	No
		Is it likely that assessment of the outcome was influenced by knowledge of intervention received?	NA	NA
	Risk-of- bias judgement			**Low risk of bias**
	Risk of bias in selection of the reported result	Were the data that produced this result analysed in accordance with a pre-specified analysis plan that was finalized before unblinded outcome data were available for analysis?	No previously published study protocol available.<br><br>"The full trial protocol is described in the Methods section, and it has	NI

			not been published separately or previously.”	
		Is the numerical result being assessed likely to have been selected, on the basis of the results, from multiple eligible outcome measurements (e.g. scales, definitions, time points) within the outcome domain?	Mean for steps presented at all time-points of measurement / net difference (95% CI) presented	No
		Is the numerical result being assessed likely to have been selected, on the basis of the results, from multiple eligible analyses of the data?	ITT and per-protocol performed and presented	No
	Risk-of- bias judgement			**some concerns**
	**Overall risk of bias score**			**Some concerns**
