# Supplementary material for: Assessing the Pragmatic Nature of Mobile Health Interventions Promoting Physical Activity: Systematic Review and Meta-analysis
Source: JMIR Mhealth Uhealth. 2023 May 4;11:e43162. doi: 10.2196/43162 (PMC10196895; doi:10.2196/43162)
Supplement: Multimedia Appendix 6 [file mhealth_v11i1e43162_app6.docx]

# RE-AIM and PRECIS-2 Methods

Data Collection Process

The RE-AIM framework was used to assess the degree to which authors reported on program factors across five domains (ie, Reach, Effectiveness, Adoption, Implementation, and Maintenance), with the assumption that comprehensive reporting favors intervention replicability and translation. ^52,53^ The evaluation was assisted by a RE-AIM coding system used in previous research ^53-55^, containing 31 items related to the RE-AIM domains. Each item was initially assigned a score of 1 (*yes*) or 0 (*no*), indicating if the authors reported this category.

In contrast, the PRECIS-2 tool determined how applicable trial components of the included studies are, with less applicable trial designs being situated at the explanatory end of the PRECIS-2 continuum. ^28^ The tool is comprised of nine domains (ie, eligibility criteria, recruitment, setting, flexibility (delivery), flexibility (adherence), follow-up, primary outcome, and primary analysis), each of which is assigned a score from 1 to 5, where, according to Loudon et al. ^28^,1 is *very explanatory*, 2 is *rather explanatory*, 3 is *equally pragmatic and explanatory*, 4 is *rather pragmatic*, and 5 is *very pragmatic*.

To update the data extract procedure specific to this work, original combined RE-AIM and PRECIS (and then PRECIS-2) coding sheets ^56^ were adapted. In the initial piloting phase, two studies were independently coded by four authors (M.P.B., D.E., S.M.H., B.P.). Following, the results were discussed, and mHealth specific, as well as general adaptations to the combined coding sheets, were applied. In brief, a higher level of detail was added to account for data that was insufficiently or inadequately reported, and the type of source from which data was extracted, was recorded. In addition, we modified the PRECIS-2 *flexibility (delivery)* domain to fit app–based interventions better. That is, the original definition was only based on the degree to which the provider (ie, clinician) determined how the intervention was implemented. We also assigned an mHealth-specific score that defined flexibility of intervention delivery from the app itself (i.e., if the app provided adapted and/or personalized content based upon the participant, or if the clinician could discern adapted content delivered by the app, then it was coded as more pragmatic). The piloting procedure was repeated with two additional studies, utilizing the finalized coding sheet (Multimedia Appendix 4). The remaining studies were coded by one author (B.P.) and reviewed by three additional authors (M.P.B., D.E., S.M.H.). Discrepancies were addressed in team meetings. The coders consulted the PRECIS-2 toolkit and RE-AIM coding companion guide during the coding process when necessary.

Synthesis of Results

RE-AIM ratings were synthesized for each dimension, with the number and percentage of studies that reported on each of the 31 items listed. In addition, the overall mean and standard deviation (SD) for items reported per intervention was calculated. PRECIS-2 ratings were summarized for each domain (mean and SD), with a brief narrative description provided. In accordance with previous research ^53^, mean scores >3.50 were deemed *primarily pragmatic*. Values between 2.50 and 3.50 were considered to be *equally pragmatic and explanatory* and scores <2.50 were rated as *primarily explanatory*. Additionally, PRECIS-2 scoring is presented in table form, and PRECIS-2 domain wheels were created to visually display the results (Multimedia Appendix 6). The full rationale of assessments is stated elsewhere (Multimedia Appendix 7). The calculations are based on all available resources (ie, main trial report, additional information) unless otherwise stated.

# Overview of Key Study Characteristics

**Table 1**. Overview of key study characteristics of included interventions.

| **1^st^ author, year** | **Study design**  **Country** | **Sample size (analyzed)**  **Study duration** | **Study population** | **Key intervention** | **Key mobile app features** | **Device-derived PA measure**  **Measuring tool** |
| --- | --- | --- | --- | --- | --- | --- |
| Direito, 2015 | Three-arm randomized controlled trial  New Zealand | 51 (51)  8-weeks | Insufficiently active, healthy young people  Age: Mean 15.67 (SD 1.15)  Male: 43% | Use of commercial smartphone apps (ie, ‘Zombies, Run!’,  ‘Get Running - Couch to 5k’) consisting of an automated 8-week exercise program | Information on running and technique, instructions (audio), social interaction, behavior change techniques (ie, intention formation, goal setting, self-monitoring, feedback on performance, review of behavior goals) | Average daily time spent in light PA, moderate PA, vigorous PA, MVPA; average daily activity counts  ActiGraph GT1M |
| Edney, 2020 | Three-arm cluster randomized controlled trial  Australia | 444 (444)  100-days | Inactive adults  Age: Mean 41.3  (SD 11.6)  Male: 25.9% | Gamified app–based PA intervention (ie, Active Team app) and use of wrist-worn pedometer (Zencro TW64S), including daily push notifications and weekly email reminders | Self-monitoring, social interaction (ie, Facebook-style newsfeed), gamification (eg, mini-challenges) | Average daily minutes of MVPA  GENEActiv |
| Fanning, 2017 | Four-arm randomized factorial trial  United States | 116 (96)  12-weeks | Healthy low-active adults  Age: Mean 41.48 (SD 7.57)  Male: 20% | Use of a base-level smartphone-app combined with two theory–based app modules (ie, goal-setting, points–based feedback), including an initial in-person goal setting session, and email/text notifications | Activity tracking, guided goal setting, feedback (ie, instant, bi-weekly, points–based), educational content | Average daily minutes of MVPA  ActiGraph GT1M or newer |
| Fukuoka, 2019 | Three-arm randomized clinical trial  United States | 210 (210)  3-months  (6-month maintenance period) | Community-dwelling physically inactive women  Age: Mean 52.11 (SD 11.0)  Male: 0% | Smartphone and pedometer–based PA intervention (ie, mPED app, Omron activity monitor), including a brief interactive in-person session (eg, goal setting, health education), and daily automated text-reminders | Daily message/video clip (reinforcement of in-person session), activity tracking, self-monitoring, goal setting | Average daily time spent in moderate PA, vigorous PA, MVPA; Mean total daily/hourly steps  Omron Active Style Pro HJA-350IT |
| Garde, 2018 | Two-arm randomized controlled trial  Canada | 42 (37)  3-weeks  (1-week maintenance period) | Elementary school students  Age: Mean 10.6 (SD 0.51)  Male: 45.2% | Use of a mobile exergame (ie, MobileKids Monster Manor) on a Wi-Fi-enabled iPod touch | Gamification (eg, mini-challenges), social interaction, activity tracking | Total daily steps; Daily active minutes  Tractivity activity monitor |
| Glynn, 2014 | Two-arm randomized controlled trial  Ireland | 90 (77)  8-weeks | Primary care patients  Age: Mean 44.1 (SD 11.5)  Male: 36% | Smartphone app PA intervention (ie, Accupedo-Pro Pedometer app), including the issuance with the Irish Heart Foundation ‘Be Active’ brochure by email | Activity tracking, automatic feedback on activity and calories burned, self-monitoring | Mean daily step count  Accupedo-Pro Pedometer app |
| Gremaud, 2018 | Two-arm randomized controlled trial  United States | 146 (144)  10-weeks | Sedentary office workers  Age: Mean 40.45  Male: 23.6% | Use of a web-app (ie, MapTrek mHealth platform) that gamifies Fitbit Zip use, and incorporates tailored text messages | Gamification, goal setting, social interaction, activity tracking, self-monitoring, automated bidirectional messages | Average daily steps; average daily active minutes  Fitbit Zip |
| Harries, 2016 | Three-arm randomized controlled trial  England | 165 (152)  8-weeks | Healthy young to early-middle-aged men  Age: ? (18-40 years)  Male: 100% | Smartphone app (ie, bActive app), including weekly motivational text messages and regular email reminders | Self-monitoring, feedback on steps and calories burned, and distance travelled (individual and group averages) | Average daily number of steps  bActive app |
| Hurkmans, 2018 | Four-arm randomized clinical trial  Belgium | 102 (102)  12-weeks | overweight adults  Age: Mean 44.96  Male: 29.6% | Use of a mobile app (ie, b-SLIM), providing digital advice on PA and information on nutrition | Self-monitoring, educational content (ie, information on PA and nutrition), social interaction, digital advice for participants dietary pattern and PA | Weekly minutes of MVPA  ActiGraph wGT3X-BT |
| King, 2016 | Four-arm randomized controlled trial  United States | 95 (89)  8-weeks | Insufficiently active, community-dwelling adults  Age: Mean 60.0 (SD 9.3)  Male: 24.7% | Use of customized PA-sedentary behavior apps, based on distinct motivational frames | Customized just-in-time feedback, activity tracking, self-monitoring, social interaction, gamification, goal-setting, problem-solving strategies | Daily minutes of MVPA  smartphone-derived accelerometry |
| Kitagawa, 2020 | Three-arm randomized controlled pilot trial  Japan | 48 (48)  2-weeks | housewives  Age: Mean 38.0 (SD 4.5)  Male: 0% | Use of Jawbone UP app with access to Jawbone UP24 wristband, including a one-time only counseling session and health pamphlet | Activity tracking, self-monitoring | Mean daily total PA; mean steps per day  Jawbone UP24 |
| Leinonen, 2017 | Two-arm randomized controlled feasibility trial  Finland | 496 (167)  6-months | Conscription-aged young men  Age: Mean 17.85  Male: 100% | Use of an automated, gamified, tailored web–based mobile service (ie, MOPOrtal) with access to wrist-worn PA monitor (ie, Polar Active) | Automatically tailored health and exercise information, gamification (ie, conquering game), feedback, social interaction | Mean daily time spent in MVPA  Polar Active |
| Lyons, 2017 | Two-group randomized controlled pilot trial  United States | 40 (40)  12-weeks | Insufficiently active, mid-aged and older adults  Age: Mean 61.5 (SD 5.6)  Male: 15% | Use of Jawbone UP app on tablet device with access to Jawbone UP24 activity monitor. Additionally, initial goal-setting session, and weekly brief telephone counseling provided | Activity tracking, self-monitoring, social interaction | Mean steps per day; minutes of total daily PA  ActivPal |
| Martin, 2015 | Randomized clinical pilot trial  United States | 48 (48)  5-weeks | Insufficiently active outpatients at a cardiovascular disease prevention center  Age: Mean 58.0 (SD 8.0)  Male: 54% | Fully automated mHealth intervention (ie, Fitbug app, display-free Fitbug Orb, and smart texts) | Self-monitoring, motivational, and positive reinforcement messages | Mean change in accelerometer-measured daily step count; changes in total daily activity time and aerobic time.  Fitbug Orb |
| Pope, 2020 | Two-arm randomized controlled pilot trial  United States | 44 (44)  10-weeks | Insufficiently active college students  Age: Mean 21.6  Male: 27.27% | Social media-delivered mHealth intervention (ie, commercial MapMyFitness app, and twice-weekly health education tips provided in private Facebook page) | Activity tracking, social interaction | Average daily minutes of MVPA  ActiGraph GT3X+ |
| Recio-Rodriguez, 2016 /  Garcia-Ortiz, 2018 | Two-arm randomized controlled trial  Spain | 833 (833)  3-months  (9-month maintenance) | Inactive primary care patients  Age: Mean 51.85  Male: 37.9% | Smartphone app–based lifestyle intervention supporting an initial 30-min counseling session on PA and the Mediterranean diet, including printed support leaflets | Feedback (dietary behavior and PA), health behavior recommendations based on activity reports | Daily steps; weekly minutes of total PA, light PA, moderate PA, vigorous PA, MVPA, total PA  ActiGraph GT3X |
| Robertson, 2018 | Two-arm cluster randomized controlled trial  Scotland | 215 (157)  5-weeks | Primary school students  Age: ?  Male: 46.5% | Location–based mobile-exergame (ie, FitQuest app) implemented in weekly mandatory physical education classes | Gamification (ie, mini-games), social interaction, goal setting, self-monitoring, feedback | Average daily step count; average daily minutes of MVPA  NL 1000 piezoelectric |
| Schade, 2020 | Two-arm randomized controlled trial  United States | ? (27)  2-weeks | Healthy undergraduate students  Age: Mean 20.96  Male: 51.9% | Use of a commercial mobile game (ie, Pokémon Go app) with access to Fitbit Charge HR activity monitor | Gamification, social interaction | Mean number of daily steps  Fitbit Charge Heart Rate FB405BKL |
| Simons, 2018 | Two-arm cluster randomized controlled trial Belgium | 130 (102)  10-weeks  (3-month maintenance period) | Lower educated, insufficient active working adults  Age: Mean 25.0 (SD 3.0)  Male: 48.5% | Use of smartphone app (ie, Active Coach app) with access to Fitbit Charge activity monitor | Self-monitoring, goal-setting, feedback, educational content (ie, practical help, and scientific facts on PA) | Average daily time spent in light PA, moderate PA, vigorous PA, MVPA, total PA; average daily steps  ActiGraph GT3X+ |
| Walsh, 2016 | Two-arm randomized controlled pilot trial  Ireland | 58 (55)  5-weeks | Young adults  Age: Mean 20.55 (SD 2.07)  Male: 27.3% | Smartphone app–based PA intervention (ie, Accupedo-Pro Pedometer app), with initial information provided (ie, benefits of walking regularly and recommended PA levels) | Activity tracking, automatic feedback on activity and calories burned, self-monitoring | Average daily step count  Accupedo-Pro Pedometer app |
| Zhang, 2019 | Two-arm randomized controlled pilot trial  United States | 91 (91)  3-months | Young African American women  Age: Mean 26.8 (SD 5.1)  Male: 0% | Mobile app–based small-group PA intervention (ie, PennFit app) with access to Fitbit Zip activity monitor, including an initial brief training session on health benefits of PA | Activity tracking, social interaction, self-monitoring | Daily steps; daily minutes of light PA, MVPA  Fitbit Zip |
| Zhou, 2018 | Two-arm randomized controlled trial  United States | 64 (64)  10-weeks | Adult university staff employees  Age: Mean 41.1 (SD 11.3)  Male: 17% | Use of an automated personalized, adaptive goal-setting smartphone app (ie, CalFit iOs app) | Activity tracking, self-monitoring, push-notifications | Mean daily steps  iPhone health chip |

Notes: PA = physical activity; MVPA = moderate-to-vigorous physical activity; SD = standard deviation. The table formatting was adapted from Romeo et al. (2019).

# RE-AIM Scoring

**Table 2.** Inclusion of RE-AIM items across all interventions (N=22).

| **RE-AIM Dimension and Items** | **% (n)**  (adequate) | **Interventions** (adequately reported)) | **% (n)**  (inadequate) | **Interventions** (inadequately reported)) |
| --- | --- | --- | --- | --- |
| **Reach** | **44.3%** |  |  |  |
| 1. Exclusion criteria | o: 50.0 (11) | 1 3 5 6 7 8 9 13 15 21 22 | o: 9.1 (2) | 16 20 |
|  | a: 27.3 (6) | 2 4 10 14 16 20 | a: 0.0 (0) | - |
| 2. Participation rate | o: 72.7 (16) | Level 1: 12 19 22  Level 2: 1 2 3 4 6 7 10 14 15 16 18 20 21 | o: 9.1 (2) | Level 1: -  Level 2: 9 11 |
|  | a: 0.0 (0) | - | a: 0.0 (0) | - |
| 3. Representativeness | o: 27.2 (6) | Indicator 1: 12  Indicator 2: 2 4 11 15 20  Indicator 3: - | o: 22.7 (5) | Indicator 1: -  Indicator 2: 7 8 14 18 22  Indicator 3: - |
|  | a: 0.0 (0) | - | a: 0.0 (0) | - |
| 4. Use of qualitative methods to understand reach and/or recruitment | o: 0.0 (0) | - | o: 0.0 (0) | - |
|  | a: 0.0 (0) | - | a: 0.0 (0) | - |
| **Effectiveness** | **52.7%** |  |  |  |
| 5. Measure of primary outcome | o: 100.0 (22) | 1 2 3 4 5 6 7 8 9 10 11 12 13 14 15 16 17 18 19 20 21 22 | o: 0.0 (0) | - |
|  | a: 0.0 (0) | - | a: 0.0 (0) | - |
| 6. Measure of broader outcomes (i.e., QoL, negative outcomes) | o: 40.9 (9) | 1 2 4 6 10 11 12 13 17 | o: 0.0 (0) | - |
|  | a: 9.1 (2) | 14 22 | a: 0.0 (0) | - |
| 7. Measure of robustness across subgroups | o: 18.2 (4) | 4 8 14 16 | o: 0.0 (0) | - |
|  | a: 0.0 (0) | - | a: 0.0 (0) | - |
| 8. Measure of short-term attrition | o: 63.6 (14) | 1 2 3 6 7 9 11 13 14 15 16 17 20 22 | o: 18.2 (4) | 5 10 12 19 |
|  | a: 0.0 (0) | - | a: 0.0 (0) | - |
| 9. Use of qualitative methods/data to understand outcomes | o: 27.3 (6) | 1 3 8 15 17 19 | o: 9.1 (2) | 5 14 |
|  | a: 4.5 (1) | 12 | a: 4.5 (1) | 6 |
| **Adoption-Setting** | **3.4%** |  |  |  |
| 10. Setting exclusions | o: 4.5 (1) | 19 | o: 4.5 (1) | 17 |
|  | a: 4.5 (1) | 17 | a: 0.0 (0) | - |
| 11. Setting adoption rate | o: 4.5 (1) | 19 | o: 0.0 (0) | - |
|  | a: 0.0 (0) | - | a: 0.0 (0) | - |
| 12. Setting representativeness | o: 0.0 (0) | - | o: 0.0 (0) | - |
|  | a: 0.0 (0) | - | a: 0.0 (0) | - |
| 13. Use of qualitative methods to understand adoption at setting level | o: 0.0 (0) | - | o: 0.0 (0) | - |
|  | a: 0.0 (0) | - | a: 0.0 (0) | - |
| **Adoption-Staff** | **0.0%** |  |  |  |
| 14. Staff exclusions | o: 0.0 (0) | - | o: 0.0 (0) | - |
|  | a: 0.0 (0) | - | a: 0.0 (0) | - |
| 15. Staff participation rate | o: 0.0 (0) | - | o: 0.0 (0) | - |
|  | a: 0.0 (0) | - | a: 0.0 (0) | - |
| 16. Staff representativeness | o: 0.0 (0) | - | o: 0.0 (0) | - |
|  | a: 0.0 (0) | - | a: 0.0 (0) | - |
| 17. Use of qualitative methods to understand staff participation | o: 0.0 (0) | - | o: 0.0 (0) | - |
|  | a: 0.0 (0) | - | a: 0.0 (0) | - |
| **Implementation** | **10.0%** |  |  |  |
| 18. Delivered as intended | o: 13.6 (3) | 7 13 17 | o: 18.2 (4) | 1 2 4 6 |
|  | a: 9.1 (2) | 14 22 | a: 0.0 (0) | - |
| 19. Adaptations to intervention | o: 9.1 (2) | 13 17 | o: 0.0 (0) | - |
|  | a: 9.1 (2) | 14 22 | a: 0.0 (0) | - |
| 20. Cost of intervention (time or money) | o: 0.0 (0) | - | o: 18.2 (4) | 9 10 16 17 |
|  | a: 0.0 (0) | - | a: 9.1 (2) | 1 2 |
| 21. Consistency of implementation across staff/ time/settings subgroups | o: 4.5 (1) | 4 | o: 4.5 (1) | 16 |
|  | a: 4.5 (1) | 16 | a: 0.0 (0) | - |
| 22. Use of qualitative methods to understand implementation | o: 0.0 (0) | - | o: 0.0 (0) | - |
|  | a: 0.0 (0) | - | a: 4.5 (1) | 6 |
| **Maintenance-Individual** | **9.0%** |  |  |  |
| 23. Measure of primary outcome at ≥6-month follow-up | o: 13.6 (3) | 2 4 16 | o: 0.0 (0) | - |
|  | a: 0.0 (0) | - | a: 0.0 (0) | - |
| 24. Measure of broader outcomes (i.e., QoL, negative outcomes) at follow-up | o: 9.1 (2) | 2 4 | o: 0.0 (0) | - |
|  | a: 0.0 (0) | - | a: 0.0 (0) | - |
| 25. Measure of long-term robustness across subgroups | o: 9.1 (2) | 4 16 | o: 0.0 (0) | - |
|  | a: 0.0 (0) | - | a: 0.0 (0) | - |
| 26. Measure of long-term attrition | o: 13.6 (3) | 2 4 16 | o: 0.0 (0) | - |
|  | a: 0.0 (0) | - | a: 0.0 (0) | - |
| 27. Use of qualitative methods to understand long-term effects | o: 0.0 (0) | - | o: 0.0 (0) | - |
|  | a: 0.0 (0) | - | a: 0.0 (0) | - |
| **Maintenance-Setting** | **3.4%** |  |  |  |
| 28. Program ongoing (≥6-month post-study funding) | o: 0.0 (0) | - | o: 0.0 (0) | - |
|  | a: 4.5 (1) | 12 | a: 0.0 (0) | - |
| 29. Long-term program adaptations | o: 9.1 (2) | 5 14 | o: 0.0 (0) | - |
|  | a: 0.0 (0) | - | a: 0.0 (0) | - |
| 30. Some discussion of sustainability of business model | o: 0.0 (0) | - | o: 0.0 (0) | - |
|  | a: 0.0 (0) | - | a: 0.0 (0) | - |
| 31. Use of qualitative methods to understand setting-level institutionalization | o: 0.0 (0) | - | o: 0.0 (0) | - |
|  | a: 0.0 (0) | - | a: 0.0 (0) | - |
| **Overall RE-AIM** | **18.1%** |  |  |  |

Notes: 1 = Direito et al. (2015), 2 = Edney et al. (2020), 3 = Fanning et al. (2017), 4 = Fukuoka et al. (2019), 5 = Garde et al. (2018), 6 = Glynn et al. (2014), 7 = Gremaud et al. (2018), 8 = Harries et al. (2016), 9 = Hurkmans et al. (2018), 10 = King et al. (2016), 11 = Kitagawa et al. (2020), 12 = Leinonen et al. (2017), 13 = Lyons et al. (2017), 14 = Martin et al. (2015), 15 = Pope and Gao (2020), 16 = Recio-Rodriguez et al. (2016); Garcia-Ortiz et al. (2018), 17 = Robertson et al. (2018), 18 = Schade et al. (2020), 19 = Simons et al. (2018), 20 = Walsh et al. (2016), 21 = Zhang and Jemmott III (2019), 22 = Zhou et al. (2018).

RE-AIM = Reach, Effectiveness, Adoption, Implementation, Maintenance (Glasgow et al., 1999); QoL = quality of life; o = original research source (ie, main trial report); a = additional research sources. The table formatting was adapted from Burke et al. (2018).

# **PRECIS-2 Scoring**

**Table 3** PRECIS-2 scoring of included interventions.

| **1^st^ author, year** | **Eligibility** | **Recruitment** | **Setting** | **Organization** | **Flexibility:**  **delivery** | **Flexibility:**  **adherence** | **Follow-up** | **Primary Outcome** | **Primary analysis** | **Total score** | **Average score (SD)** |
| --- | --- | --- | --- | --- | --- | --- | --- | --- | --- | --- | --- |
| Direito, 2015 | 4 | 3 | 3 | 4 | 3 | 5 | 2 | 2 | 5 | 31 | 3.44 (1.13) |
| Edney, 2020 | 4 | 2 | 5 | 4 | 3 | 3 | 3 | 3 | 3 | 30 | 3.33 (O.87) |
| Fanning, 2017 | 3 | 2 | 4 | 2 | 3 | 3 | 2 | 2 | 3 | 24 | 2.67 (0.71) |
| Fukuoka, 2019 | 2 | 2 | 2 | 1 | 1 | 3 | 2 | 3 | 5 | 21 | 2.33 (1.23) |
| Garde, 2018 | 4 | 4 | 5 | 4 | 3 | 5 | 3 | 2 | 2 | 32 | 3.56 (1.13) |
| Glynn, 2014 | 4 | 5 | 4 | 2 | 2 | 4 | 1 | 4 | 2 | 28 | 3.11 (1.36) |
| Gremaud, 2018 | 3 | 3 | 3 | 2 | 2 | 4 | 2 | 3 | 2 | 24 | 2.67 (0.71) |
| Harries, 2016 | 2 | 1 | 5 | 3 | 3 | 2 | 3 | 4 | 3 | 26 | 2.89 (1.17) |
| Hurkmans, 2018 | 1 | 2 | 1 | 1 | 1 | 4 | 2 | 3 | 3 | 18 | 2.00 (1.12) |
| King, 2016 | 2 | 1 | 3 | 2 | 3 | 3 | 2 | 2 | 3 | 21 | 2.33 (0.71) |
| Kitagawa, 2020 | 3 | 2 | 3 | 1 | 2 | 4 | 2 | 4 | 4 | 25 | 2.78 (1.09) |
| Leinonen, 2017 | 5 | 5 | 4 | 4 | 3 | 4 | 2 | 3 | 3 | 33 | 3.67 (1.00) |
| Lyons, 2017 | 3 | 3 | 3 | 1 | 2 | 2 | 2 | 2 | 4 | 22 | 2.44 (0.88) |
| Martin, 2015 | 4 | 5 | 5 | 4 | 3 | 5 | 4 | 5 | 5 | 40 | 4.44 (0.73) |
| Pope, 2020 | 3 | 3 | 3 | 2 | 2 | 5 | 2 | 2 | 4 | 26 | 2.89 (1.05) |
| Recio-Rodriguez, 2016 /  Garcia-Ortiz, 2018 | 3 | 5 | 5 | 2 | 2 | 4 | 1 | 3 | 3 | 28 | 3.11 (1.36) |
| Robertson, 2018 | 5 | 4 | 5 | 2 | 4 | 2 | 2 | 2 | 3 | 29 | 3.22 (1.30) |
| Schade, 2020 | 5 | 4 | 3 | 3 | 2 | 4 | 3 | 3 | 1 | 28 | 3.11 (1.17) |
| Simons, 2018 | 2 | 2 | 5 | 1 | 3 | 4 | 1 | 2 | 2 | 22 | 2.44 (1.33) |
| Walsh, 2016 | 4 | 2 | 1 | 2 | 2 | 4 | 3 | 3 | 3 | 24 | 2.67 (1.00) |
| Zhang, 2019 | 3 | 2 | 1 | 2 | 2 | 4 | 2 | 3 | 4 | 23 | 2.56 (1.01) |
| Zhou, 2018 | 2 | 3 | 3 | 3 | 2 | 4 | 2 | 4 | 4 | 27 | 3.00 (0.87) |
| **Overall averages (SD)** | 3.23 (1.10) | 2.95  (1.29) | 3.45 (1.37) | 2.36  (1.09) | 2.41  (0.73) | 3.73  (0.93) | 2.18 (0.73) | 2.91  (0.86) | 3.23  (1.06) |  | **2.93 (0.54)** |

Notes: PRECIS-2 = PRagmatic– Explanatory Continuum Indicator Summary-2 (Loudon et al., 2015); SD = Standard Deviation. The table formatting was adapted from Fitzpatrick et al. (2020).

# PRECIS-2 Wheels




















.

# Meta-Analysis


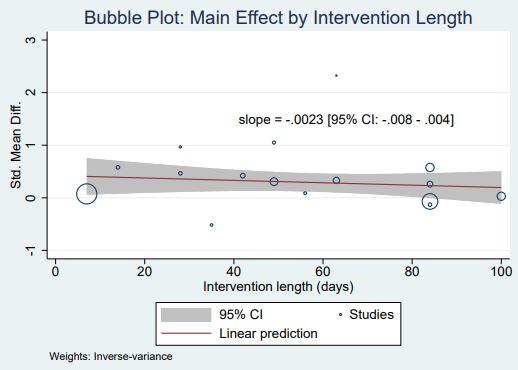


**Figure 24**. Bubble plot of treatment effects on intervention duration (a single outlier was removed).

Note: CI = Confidence Interval.


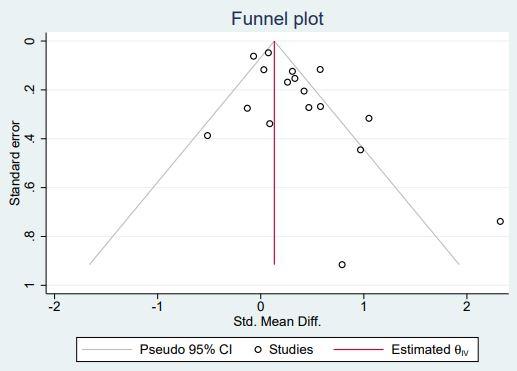


**Figure 25.** Funnel plot of included studies in this meta-analysis.

**Table 4.** Egger’s test specifications.

| **Egger’s test specification:** | **Z-score** | ***P* value** |
| --- | --- | --- |
| Restricted maximum likelihood | 2.46 | 0.014 |
| Empirical Bayes | 2.14 | 0.032 |
| DerSimonian-Laird | 2.52 | 0.012 |
| Hedges | 1.92 | 0.055 |
| Hunter-Schmidt | 2.75 | 0.006 |
